# S3-Leitlinie Sepsis – Prävention, Diagnose, Therapie und Nachsorge – Update 2025

**DOI:** 10.1007/s00063-025-01317-1

**Published:** 2025-08-18

**Authors:** Frank M. Brunkhorst, Michael Adamzik, Hubertus Axer, Michael Bauer, Christian Bode, Hans-Georg Bone, Thorsten Brenner, Michael Bucher, Sascha David, Maximilian Dietrich, Christian Eckmann, Gunnar Elke, Torben Esser, Thomas Felbinger, Christine Geffers, Herwig Gerlach, Béatrice Grabein, Matthias Gründling, Ulf Günther, Stefan Hagel, Andreas Hecker, Stefan Henkel, Babila Janusan, Stefan John, Achim Jörres, Achim Kaasch, Stefan Kluge, Matthias Kochanek, Agnieszka Lajca, Gernot Marx, Konstantin Mayer, Patrick Meybohm, Onnen Mörer, Michael Oppert, Vladimir Patchev, Mathias Pletz, Christian Putensen, Tim Rahmel, Jenny Rosendahl, Rolf Rossaint, Bernd Salzberger, Michael Sander, Stefan Schaller, Christina Scharf-Janssen, Felix Schmitt, Matthias Unterberg, Markus Weigand, Arved Weimann, Sebastian Weis, Björn Weiß, Alexander Wolf, Alexander Zarbock

**Affiliations:** 1 Klinik für Anästhesiologie und Intensivtherapie, Universitätsklinikum Jena, Am Klinikum 1, 07747 Jena, Deutschland; 2 Klinik für Anästhesiologie, Intensivmedizin und Schmerztherapie am Universitätsklinikum Knappschaftskrankenhaus Bochum, Bochum, Deutschland; 3 Klinik für Neurologie, Universitätsklinikum Jena, Jena, Deutschland; 4 Klinik für Anästhesiologie und Operative Intensivmedizin, Universitätsklinikum Bonn, Bonn, Deutschland; 5 Zentrum für Anästhesiologie, Intensivmedizin und Schmerztherapie, Knappschaft Kliniken, Recklinghausen, Deutschland; 6 Klinik für Anästhesiologie und Intensivmedizin, Universitätsmedizin Essen, Essen, Deutschland; 7 Klinik für Anästhesiologie und Intensivmedizin, Universitätsklinikum Halle, Halle (Saale), Deutschland; 8 Institut für Intensivmedizin des Universitätsspital Zürich, Zürich, Schweiz; 9 Klinik für Anästhesiologie, Universitätsklinikum Heidelberg, Heidelberg, Deutschland; 10 Klinik für Allgemein-, Viszeral- und Thoraxchirurgie am Klinikum Hann. Münden, Hann. Münden, Deutschland; 11 Klinik für Anästhesie, Intensivmedizin, Schmerztherapie & Notfallmedizin, St. Bernward Krankenhaus Hildesheim, Kiel, Deutschland; 12 Institut für Medizinische Mikrobiologie und Krankenhaushygiene, Otto-von-Guericke-Universität Magdeburg, Magdeburg, Deutschland; 13 Klinik für Anästhesiologie, Operative Intensivmedizin und Schmerztherapie, Klinikum Neuperlach, München, Deutschland; 14 Institut für Hygiene und Umweltmedizin der Charité, Universitätsmedizin Berlin, Berlin, Deutschland; 15 Deutsche Sepsis-Gesellschaft, Berlin, Berlin, Deutschland; 16 Klinische Mikrobiologie und Krankenhaushygiene, Ludwig-Maximilians-Universität München, München, Deutschland; 17 Klinik für Anästhesie, Intensiv‑, Notfall- und Schmerzmedizin, Universität Greifswald, Greifswald, Deutschland; 18 Universitätsklinik für Anästhesiologie Intensivmedizin/Notfallmedizin/Schmerztherapie, Klinikum Oldenburg, Oldenburg, Deutschland; 19 Institut für Infektionsmedizin und Krankenhaushygiene, Universitätsklinikum Jena, Jena, Deutschland; 20 Klinik für Allgemein‑, Viszeral‑, Thorax‑, Transplantations- und Kinderchirurgie, Universitätsklinikum Gießen, Gießen, Deutschland; 21 Klinik für Anästhesiologie, operative Intensivmedizin und Schmerztherapie, Universitätsklinikum Gießen, Gießen, Deutschland; 22 Zentrum für Intensivmedizin, Klinikum Nürnberg, Nürnberg, Deutschland; 23 Klinik für Nephrologie, Transplantationsmedizin und internistische Intensivmedizin, Kliniken Köln, Köln, Deutschland; 24 Klinik für Intensivmedizin, Universitätsklinikum Hamburg-Eppendorf, Hamburg, Deutschland; 25 Medizinische Klinik 1, Schwerpunkt Intensivmedizin, Uniklinik Köln, Köln, Deutschland; 26 Klinik für Operative Intensivmedizin und Intermediate Care, Universitätsklinikum Aachen, Aachen, Deutschland; 27 Klinik für Pneumologie und Schlafmedizin, St. Vincentius-Kliniken, Karlsruhe, Karlsruhe, Deutschland; 28 Klinik für Anästhesiologie, Intensivmedizin, Notfallmedizin und Schmerztherapie, Universitätsklinikum Würzburg, Würzburg, Deutschland; 29 Klinik für Anästhesiologie, Universitätsmedizin Göttingen, Göttingen, Deutschland; 30 Notfall- und Intensivmedizin, Klinikum Ernst von Bergmann, Potsdam, Potsdam, Deutschland; 31 Institut für Psychosoziale Medizin, Psychotherapie und Psychoonkologie, Universitätsklinikum Jena, Jena, Deutschland; 32 Klinik für Anästhesiologie, Uniklinik RWTH Aachen, Aachen, Deutschland; 33 Klinik und Poliklinik für Innere Medizin I, Universitätsklinikum Regensburg, Regensburg, Deutschland; 34 Klinik für Anästhesiologie, operative Intensivmedizin und Schmerztherapie, Universitätsklinikum Gießen, Gießen, Deutschland; 35 Klinische Abteilung für Allgemeine Anästhesie und Intensivmedizin, Universitätsklinik für Anästhesie, allgemeine Intensivmedizin und Schmerztherapie, Medizinische Universität Wien, Wien, Österreich; 36 Klinik für Anästhesiologie, LMU München, München, Deutschland; 37 Klinik für Allgemein‑, Viszeral- und Onkologische Chirurgie, Klinikum St. Georg, Leipzig, Deutschland; 38 Leibniz Institute for Natural Product Research and Infection Biology-HKI, Jena University Hospital, Jena, Deutschland; 39 Klinik für Anästhesiologie und Intensivmedizin, Charité – Universitätsmedizin Berlin, Berlin, Deutschland; 40 Klinik für Anästhesiologie, operative Intensivmedizin und Schmerztherapie, Universitätsklinikum Münster, Münster, Deutschland

**Keywords:** Sepsis, Septischer Schock, Diagnose, Therapie, Nachsorge, Sepsis, Septic shock, Diagnosis, Treatment, Aftercare

## Abstract

**Hintergrund:**

Sepsis ist eine akut lebensbedrohliche multiple Organdysfunktion, ausgelöst durch eine Infektion.

**Methodik:**

Bei der vorliegenden Leitlinie handelt es sich um ein Update der S3-Leitlinie „Sepsis – Prävention, Diagnose, Therapie und Nachsorge“ (AMWF-Register-Nr.: 079–001) der Deutschen Sepsis-Gesellschaft (DSG) vom 31.12.2018. Dabei wurde das Update der „Surviving sepsis campaign (SSC): international guidelines for management of sepsis and septic shock 2021“ vom 04.10.2021 als Referenzleitlinie zugrunde gelegt. Die DSG-Leitlinienkommission verglich jede Empfehlung zu den zugrunde liegenden PICO-Fragen der DSG-Leitlinie 2018 (Literaturrecherche bis 12/2018) mit denen der SSC-Leitlinie 2021 (Literaturrecherche bis 07/2019) und bewertete die in der Zwischenzeit neu verfügbare publizierte Datenlage (Literaturrecherche bis 12/2024) mittels systematischer Aktualisierungsrecherchen und Literaturbewertungen unter Befolgung des Regelwerkes des GRADE-Systems und der AWMF.

**Ergebnisse:**

Insgesamt wurden 88 PICO-Fragen u. a. zur Diagnose und Therapie der Infektion und des Organversagens adressiert. Davon wurden 2 als Statements, 29 als Expertenkonsens und 57 als evidenzbasierte Empfehlungen (26 mit starkem und 31 mit schwachem Empfehlungsgrad) konsentiert. Im Vergleich zur Vorgänger-Leitlinie 2018 wurden 43 Empfehlungen überprüft, aber beibehalten. 16 Empfehlungen wurden geändert, und 29 Empfehlungen wurden neu ausgesprochen.

**Schlussfolgerung:**

Angesichts fehlender Evidenz für zahlreiche Maßnahmen zur stationären Versorgung von Patienten mit Sepsis oder septischem Schock wurden alte und neue Wissenslücken offenbart. Bei den evidenzbasierten Empfehlungen war die zugrunde liegende Evidenzqualität nach GRADE nur bei 5 Empfehlungen hoch, bei 18 Empfehlungen moderat, bei 17 Empfehlungen niedrig und bei 16 sehr niedrig. Diese Evidenzlücken können nur durch zukünftige multizentrische, nichtkommerzielle klinische Prüfungen geschlossen werden. Das Update der S3-Leitlinie Sepsis beinhaltet einige Aktualisierungen zu Empfehlungen der Vorgängerleitlinie. Diese Aktualisierungen werden in einige der fall- und einrichtungsbezogenen QS-Indikatoren des QS-Verfahrens 2025 einfließen müssen. Beeinträchtigungen in der gesundheitsbezogenen Lebensqualität müssen bei Überlebenden mehr in den Fokus der ambulanten Versorgung gerückt werden.

## Abstract (en)

**Background:**

Sepsis is an acute, life-threatening multiple organ dysfunction triggered by an infection.

**Methods:**

This guideline is an update of the S3 guideline “Sepsis—prevention, diagnosis, therapy, and follow-up care” (Arbeitsgemeinschaft der Wissenschaftlichen Medizinischen Fachgesellschaft [AMWF] Registry No. 079–001) of the German Sepsis Society (DSG) dated 31 December 2018. The update of the “Surviving sepsis campaign (SSC): international guidelines for management of sepsis and septic shock 2021” dated 4 October 2021, was used as the reference guideline. The DSG Guideline Commission compared each recommendation on the underlying PICO questions of the DSG Guideline 2018 (literature search until December 2018) with those of the SSC Guideline 2021 (literature search until July 2019) and evaluated the newly available published data (literature search until December 2024) by means of systematic update searches and literature reviews in compliance with the rules of the Grading of Recommendations, Assessment, Development and Evaluation (GRADE) system and the AWMF.

**Results:**

A total of 88 PICO questions were addressed, including those related to the diagnosis and treatment of infection and organ failure. Of these, two were agreed upon as statements, 29 as expert consensus, and 57 as evidence-based recommendations (26 with a strong and 31 with a weak recommendation grade). Compared to the previous 2018 guideline, 43 recommendations were reviewed but retained, 16 recommendations were modified, and 29 recommendations were newly issued.

**Conclusion:**

Given the lack of evidence for numerous measures for the inpatient care of patients with sepsis or septic shock, old and new knowledge gaps were revealed. Among the evidence-based recommendations, the underlying GRADE quality of evidence was high for only 5 recommendations, moderate for 18 recommendations, low for 17 recommendations, and very low for 16. These evidence gaps can only be closed through future multicenter, noncommercial clinical trials. The update to the S3 guideline on sepsis includes some updates to the recommendations of the previous guideline. These updates will need to be incorporated into some of the case- and facility-specific quality assurance indicators of quality assurance (QA) procedure 2025. Impairments in health-related quality of life for survivors must be given greater focus in outpatient care.

## Infobox

Beteiligte Fachgesellschaften

- *Federführend*: Deutsche Sepsis-Gesellschaft e. V. (DSG)
- Deutsche Gesellschaft für Anästhesiologie und Intensivmedizin e. V. (DGAI)
- Deutsche Gesellschaft für Chirurgie (DGCH)
- Deutsche Gesellschaft für Internistische Intensivmedizin und Notfallmedizin (DGIIN)
- Deutsche Gesellschaft für Ernährungsmedizin e. V. (DGEM)
- Deutsche Gesellschaft für Neurologie (DGN)
- Deutsche Gesellschaft für Infektiologie (DGI)
- Deutsche Gesellschaft für Nephrologie e. V. (DGfN)
- Deutsche Interdisziplinäre Vereinigung für Intensiv- und Notfallmedizin (DIVI)
- Deutsche Gesellschaft für Pneumologie und Beatmungsmedizin (DGP)
- Deutsche Gesellschaft für Hygiene und Mikrobiologie (DGHM)
- Deutsche Gesellschaft für Hämatologie und. Medizinische Onkologie e. V (DGHO)
- Deutsche Gesellschaft für Medizinische Psychologie (DGMP)
- Deutsche Sepsis-Hilfe e. V. (DSH)
- Nationales Referenzzentrum für Surveillance von nosokomialen Infektionen (NRZ)
- Paul-Ehrlich-Gesellschaft für Infektionstherapie e. V. (PEG)

## Was gibt es Neues?

Bei der vorliegenden Leitlinie der Klassifikation S3 handelt es sich um ein Update der S3-Leitlinie „Sepsis – Prävention, Diagnose, Therapie und Nachsorge“ [1] (AMWF-Register-Nr.: 079–001) der Deutschen Sepsis-Gesellschaft (DSG) vom 31.12.2018. Dabei wurde das Update der *„Surviving sepsis campaign (SSC): international guidelines for management of sepsis and septic shock 2021“* [2] vom 04.10.2021 als Referenzleitlinie zugrunde gelegt. Die DSG-Leitlinienkommission verglich jede Empfehlung zu den zugrunde liegenden PICO-Fragen der DSG-Leitlinie 2018 (Literaturrecherche bis Dezember 2018) mit denen der SSC-Leitlinie 2021 (Literaturrecherche bis Juli 2019) und bewertete gegebenenfalls die in der Zwischenzeit neu verfügbare publizierte Datenlage mittels systematischer Aktualisierungsrecherchen und Literaturbewertungen unter Befolgung des Regelwerkes des GRADE-Systems [3]. Außerdem fanden aktuelle deutsche Leitlinien besondere Berücksichtigung, u. a. die S3-Leitlinie „Invasive Beatmung und Einsatz extrakorporaler Verfahren bei akuter respiratorischer Insuffizienz“ [4] (AWMF-Reg.-Nr.: 001–021), die S3-Leitlinie „Nierenersatztherapie in der Intensiv- und Notfallmedizin“ [5] (AWMF-Reg.-Nr.: 040–017), sowie der S3-Leitlinie „Lagerungstherapie und Mobilisation von kritisch Erkrankten auf Intensivstationen“ [6] (AWMF-Reg.-Nr.: 001–015).

In der Leitlinie wird mit aufgeführt, ob die jeweilige Kernaussage eine Adoption bzw. Adaptation der Empfehlungen der SSC-Leitlinie bzw. anderer Leitlinien darstellt, eine Änderung des Empfehlungsgrades, des Empfehlungstextes oder eine Aktualisierung der Evidenz im Vergleich zur SSC-Empfehlung bzw. anderer Leitlinien vorliegt oder eine zusätzliche DSG-Leitlinienempfehlung erstellt wurde. Die Kernaussagen der einzelnen Kapitel sind als Empfehlungen oder als Stellungnahme aufgezeigt.

Die Erweiterung des Kapitels „Nachsorge“ adressiert Aspekte der Langzeitfolgen nach überlebter Sepsis, welche als Teil des sogenannten PICS (Post Intensive Care Syndrome) zusammengefasst werden.

### Die wichtigsten Empfehlungen auf einen Blick

Die folgenden Schwerpunkte (Kap. 2–10 dieser Leitlinien-Langfassung) wurden systematisch behandelt: (Kap. 2) Screening und Erstmaßnahmen, (Kap. 3) Infektion und antimikrobielle Therapie, (Kap. 4) Kreislauftherapie, (Kap. 5) Beatmungstherapie, (Kap. 6) Nierenersatzverfahren, (Kap. 7) Ernährungstherapie, (Kap. 8) Weitere Maßnahmen, (Kap. 9) Behandlungsziele und Spätfolgen, (Kap. 10) Präventionsmaßnahmen und Impfungen. Insgesamt wurden 88 PICO-Fragen adressiert. Davon wurden 2 als Statements, 29 als Expertenkonsens und 57 als evidenzbasierte Empfehlungen (27 mit starkem und 30 mit schwachem Empfehlungsgrad) konsentiert. *Neue *(insgesamt 29) oder *geänderte* (insgesamt 16) Empfehlungen sind unten stehend aufgeführt und sind den Empfehlungen der DSG-2018-Leitlinie gegenübergestellt.

## Screening und Erstmaßnahmen

### Screening

**DSG 2025 *(Änderung):*** Wir empfehlen Krankenhäusern ein Qualitätssicherungsprogramm zum Thema Sepsis, einschließlich eines Sepsis-Screenings für akut erkrankte Hochrisikopatienten und Standardarbeitsanweisungen (SOPs) für die Behandlung (*Empfehlungsgrad: stark).*

**DSG 2018: **Wir empfehlen Krankenhäusern, Programme zur Implementierung der Leitlinie zu initiieren und umzusetzen, einschließlich eines Screenings für Risikopatienten (*Expertenkonsens).*

**DSG 2025 *(Änderung):*** Wir empfehlen bei Patienten außerhalb der Intensivstation, bei denen der Verdacht auf eine Infektion besteht, regelmäßig ein geeignetes Screeninginstrument anzuwenden, um eine Sepsis frühzeitig zu erkennen (*Empfehlungsgrad: stark).*

**DSG 2018: **Wir empfehlen, bei Patienten außerhalb von Intensivstationen, bei denen der Verdacht auf eine Infektion besteht, den Quick-Sequential-Organ-Failure-Assessment(qSOFA)-Score regelmäßig zu bestimmen, um Risikopatienten mit vitaler Bedrohung frühzeitig zu erkennen (*Expertenkonsens).*

**DSG 2025 *(NEU):*** Wir empfehlen bei Erwachsenen mit Verdacht auf Sepsis, eine Messung des Laktats im Blut durchzuführen (*Empfehlungsgrad: stark).*

**DSG 2018:** keine Empfehlung.

Initiale hämodynamische Stabilisierung

**DSG 2025 *(NEU):*** Wir schlagen vor, bei Patienten mit Sepsis oder septischem Schock, Vasopressoren auch periphervenös zu applizieren, um den MAP auf > 65 mm Hg anzuheben und nicht auf die Anlage eines ZVK zu warten (*Expertenkonsens).*

**DSG 2018:** keine Empfehlung.

### Flüssigkeitstherapie

**DSG 2025 *(NEU):*** Wir schlagen vor, bei Patienten mit septischem Schock die Rekapillarisierungszeit zur Beurteilung der Gewebeperfusion zu nutzen (*Empfehlungsgrad: schwach).*

**DSG 2018:** keine Empfehlung

## Infektion und antimikrobielle Therapie

### Beginn der antimikrobiellen Therapie

**DSG 2025 *(Änderung):*** Wir empfehlen, *bei Patienten mit Sepsis*, dass die Verabreichung von intravenösen Antiinfektiva so schnell wie möglich, *bei Patienten mit einem septischen Schock* idealerweise innerhalb einer Stunde erfolgt (*Empfehlungsgrad: stark).*

**DSG 2018: **Wir empfehlen, dass die Verabreichung von intravenösen Antiinfektiva so schnell wie möglich, idealerweise innerhalb einer Stunde, nach der Diagnose einer Sepsis oder eines septischen Schocks erfolgt (*Empfehlungsgrad: stark).*

**DSG 2025 *(NEU):*** Wir schlagen vor, bei einem Verdacht auf eine *Sepsis ohne Schocksymptomatik* zunächst eine zeitnahe weitere Diagnostik durchzuführen. Falls der Verdacht auf eine zugrunde liegende Infektion weiterbesteht, sollte die Verabreichung der antiinfektiven Therapie innerhalb von drei Stunden erfolgen (*Expertenkonsens).*

**DSG 2018:** keine Empfehlung.

### Wahl der antimikrobiellen Therapie

**DSG 2025 *(NEU):*** Wir empfehlen, bei Patienten mit Sepsis oder septischem Schock Beta-Laktam-Antibiotika als prolongierte oder kontinuierliche Infusion (nach initialem Bolus) zu verabreichen (*Empfehlungsgrad: stark).*

**DSG 2018: **keine Empfehlung.

**DSG 2025 *(NEU):*** Wir schlagen vor, bei Patienten mit Sepsis oder septischem Schock ohne erhöhtes Risiko für multiresistente Erreger KEINE routinemäßige, initiale Therapie mit zwei verschiedenen gegen gramnegative Erreger wirksamen Substanzen („double gram-negative coverage“) durchzuführen (*Empfehlungsgrad: schwach).*

**DSG 2028:** keine Empfehlung.

**DSG 2025 *(Änderung):*** Wir schlagen vor, bei Patienten mit Sepsis oder septischem Schock mit einem niedrigen Risiko für eine Pilzinfektion KEINE antimykotische Therapie durchzuführen. Wir schlagen vor, bei Patienten mit Sepsis oder septischem Schock mit einem hohen Risiko für eine Pilzinfektion eine empirische antimykotische Therapie durchzuführen (*Empfehlungsgrad: schwach).*

**DSG 2018:** Wir empfehlen, die Indikation einer zusätzlichen kalkulierten antimykotischen oder antiviralen Therapie bei Risikopatienten entsprechend der fokusbezogenen Leitlinie zu überprüfen (*Expertenkonsens).*

## Kreislauftherapie

### Flüssigkeitstherapie

**DSG 2025 *(NEU):*** Wir schlagen vor, bei Patienten mit Sepsis und septischem Schock Gelatine NICHT anzuwenden.^1^*(Empfehlungsgrad: schwach).*

**DSG 2018: **keine Empfehlung.

**DSG 2025 *(Änderung):*** Wir schlagen vor, bei Patienten mit Sepsis oder septischem Schock Albumin additiv zu balancierten Kristalloiden zu verabreichen, wenn große Mengen an Flüssigkeit benötigt werden, um eine hämodynamische Stabilität zu erreichen.^1^ *(Empfehlungsgrad: schwach).*

**DSG 2018: **Wir schlagen vor, dass von einer Verwendung von Albumin oder Gelatine bei der Behandlung von Patienten mit septischem Schock abgesehen wird, sofern eine adäquate Flüssigkeitstherapie mit Kristalloiden in der Lage ist, die hämodynamische Stabilität zu erreichen. Für den Fall, dass dies nicht möglich sein sollte, schlagen wir den ergänzenden Einsatz von Albumin oder Gelatine vor (*Empfehlungsgrad: schwach).*

### Vasoaktive Substanzen

**DSG 2025 *(Änderung):*** Wir schlagen vor, bei Patienten mit septischem Schock Vasopressin zu ergänzen, wenn mit Noradrenalin alleine kein ausreichender Blutdruck erzielt werden kann (*Empfehlungsgrad: schwach).*

**DSG 2018: **Wir schlagen vor, dass entweder Vasopressin oder Epinephrin zu Norepinephrin ergänzt wird, wenn mit Noradrenalin alleine kein ausreichender Blutdruck erzielt werden kann (*Empfehlungsgrad: schwach).*

**DSG 2025 *(NEU):*** Aufgrund der aktuellen Datenlage kann bei Patienten mit septischem Schock weder für noch gegen den Einsatz von Methylenblau eine Empfehlung ausgesprochen werden (*Expertenkonsens).*

**DSG 2018:** keine Empfehlung.

**DSG 2025 *(NEU):*** Wir schlagen vor, bei Patienten mit septischem Schock Terlipressin NICHT anzuwenden (*Empfehlungsgrad: schwach).*

**DSG 2018: **keine Empfehlung.

### Venoarterielle ECMO

**DSG 2025 *(NEU):*** Der Einsatz einer venoarteriellen ECMO bei Patienten mit einem therapierefraktären septischen Schock *ohne* einen zusätzlichen kardiogenen Schock kann NICHT empfohlen werden (*Expertenkonsens).*

Aufgrund der aktuellen Datenlage kann weder für noch gegen den Einsatz einer venoarteriellen ECMO bei Patienten mit einem therapierefraktären septischen *und* einem zusätzlichen kardiogenen Schock eine Empfehlung ausgesprochen werden (*Expertenkonsens).*

**DSG 2018: **keine Empfehlung.

## Beatmungstherapie

### Beatmungseinstellungen

**DSG 2025 *(Änderung):*** Wir empfehlen, Patienten mit Sepsis oder septischem Schock und ARDS mit einem Tidalvolumen um 6 ml/kg IBW (4–8 ml/kg IBW) zu beatmen (*Empfehlungsgrad: stark).*

**DSG 2018: **Wir empfehlen die Beatmung von Patienten mit ARDS mit einem VT ≤ 6 ml/kg Standard-Körpergewicht (KG) (*Empfehlungsgrad: stark).*

**DSG 2025 *(Änderung)*:** Wir schlagen vor, bei der invasiven Beatmung von Patienten mit Sepsis oder septischem Schock und ARDS eine inspiratorische Druckdifferenz von ≤ 14 cmH_2_O anzustreben (*Empfehlungsgrad: schwach).*

**DSG 2018:** Wir empfehlen, bei der invasiven Beatmung von Patienten mit ARDS den endinspiratorischen Atemwegsdruck (PEI) ≤ 30 cmH_2_O zu halten (*Empfehlungsgrad: stark).*

PEEP-Einstellung

**DSG 2025 *(NEU):*** Wir schlagen vor, bei Patienten mit Sepsis oder septischem Schock und moderatem oder schwerem ARDS die Einstellung des PEEP mit Hilfe einer in der Tabelle vorgeschlagenen bettseitigen Methoden zu individualisieren (*Empfehlungsgrad: schwach).*

**DSG 2018: **keine Empfehlung.

### Inspiratorische Sauerstoffkonzentration

**DSG 2025 *(NEU):*** Wir schlagen vor, bei invasiv beatmeten Patienten mit Sepsis oder septischem Schock eine SaO_2_ bzw. SpO_2_ von 92–96 % bzw. eine paO_2_ zwischen 70–90 mm Hg (9,3–12 kPa) anzustreben (*Empfehlungsgrad: schwach).*

**DSG 2018:** keine Empfehlung.

Rekrutierungsmanöver

**DSG 2025 *(Änderung):*** Wir empfehlen bei Patienten mit Sepsis oder septischem Schock KEINE Durchführung von prolongierten (> 60 s) Rekrutierungsmanövern (*Empfehlungsgrad: stark).*

**DSG 2025 *(Änderung):*** Wir schlagen vor, bei Patienten mit Sepsis oder septischem Schock KEINE routinemäßige Durchführung von kurzen (< 60 s) Rekrutierungsmanövern durchzuführen (*Empfehlungsgrad: schwach).*

**DSG 2018:** Wir schlagen vor, bei invasiv beatmeten Patienten mit ARDS keine Rekrutierungsmanöver durchzuführen (*Empfehlungsgrad: schwach).*

**DSG 2018:** Wir empfehlen bei invasiv beatmeten Patienten mit ARDS keine Durchführung von Rekrutierungsmanövern mit endinspiratorischen Drücken über 50 cmH_2_O (*Empfehlungsgrad: stark).*

### Nichtinvasive Atmungsunterstützung

**DSG 2025 *(Änderung):*** Wir schlagen vor, bei Patienten mit Sepsis oder septischem Schock und milder bis moderater akuter hypoxämischer respiratorischer Insuffizienz (PaO_2_/FiO_2_: 100–300) primär eine nichtinvasive Atmungsunterstützung (NIV, CPAP oder HFNO) anzuwenden (*Empfehlungsgrad: schwach).*

**DSG 2018: **Wir sprechen keine Empfehlung hinsichtlich der Verwendung einer nichtinvasiven Beatmung bei Patienten mit Sepsis-induziertem ARDS aus (Statement).

### Bauchlagerung

**DSG 2025 *(Änderung):*** Wir empfehlen, bei Patienten mit Sepsis oder septischem Schock eine Bauchlagerung über mindestens 12 h, vorzugsweise 16 h durchzuführen. Wir empfehlen, die Bauchlagerung frühzeitig zu erwägen und nach Indikationsstellung unverzüglich umzusetzen (*Empfehlungsgrad: stark)*

**DSG 2018:** Wir schlagen vor, dass ein Bauchlagerungsintervall von mindestens 16 h angestrebt werden soll. Die Bauchlagerung sollte frühzeitig erwogen und nach Indikationsstellung unverzüglich umgesetzt werden (*Empfehlungsgrad: schwach).*

### ECMO

**DSG 2025 *(NEU):*** Wir schlagen vor, den Einsatz einer venovenösen ECMO bei Patienten mit Sepsis oder septischem Schock und schwerem ARDS nur nach Ausschöpfen der konservativen Therapiemaßnahmen, einschließlich der Durchführung einer Bauchlagerung, und fortbestehender schwerer Gasaustauschstörung zu erwägen (*Empfehlungsgrad: schwach).*

**DSG 2018: **keine Empfehlung.

## Nierenersatztherapie

### Indizierung und Durchführung

**DSG 2025 *(NEU):*** Wir empfehlen, dass extrakorporale Nierenersatzverfahren auf Intensivstationen ausschließlich durch Fachärzte mit der Zusatzbezeichnung „Intensivmedizin“ und/oder Nephrologen indiziert und durchgeführt werden (*Expertenkonsens).*

**DSG 2018:** keine Empfehlung.

Start der Nierenersatztherapie

**DSG 2025 *(NEU):*** Wir empfehlen, bei Patienten mit Sepsis oder septischem Schock und lebensbedrohlichen Veränderungen des Flüssigkeits‑, Säure-Basen- oder Elektrolythaushaltes unverzüglich mit einer Nierenersatztherapie zu beginnen (*Expertenkonsens).*

**DSG 2018:** keine Empfehlung.

**DSG 2025 *(NEU):*** Wir schlagen vor, bei nichtlebensbedrohlichen Veränderungen und Unklarheit, ob eine Nierenersatztherapie zu erwarten ist, konservative Maßnahmen zur Vermeidung eines Nierenersatzverfahrens unter regelmäßiger Reevaluation durchzuführen (*Empfehlungsgrad: schwach).*

**DSG 2018:** keine Empfehlung.

### Diffusion versus Konvektion

**DSG 2025 *(NEU):*** Wir schlagen vor, bei Patienten mit einer Sepsis und Indikation zur Nierenersatztherapie diffusive Nierenersatzverfahren oder konvektive Verfahren bzw. eine Kombination aus diffusiven und konvektiven Verfahren gleichwertig einzusetzen (*Empfehlungsgrad: schwach).*

**DSG 2018***: *keine Empfehlung.

### Regionale und systemische Antikoagulation

**DSG 2025 *(NEU):*** Wir empfehlen, bei Patienten mit Sepsis oder septischem Schock eine regionale Citrat-Antikoagulation oder eine systemische Heparin-Antikoagulation des extrakorporalen Blutkreislaufs gleichwertig einzusetzen (*Empfehlungsgrad: stark).*

**DSG 2018:** keine Empfehlung.

### Adäquate Dosis

**DSG 2025 *(NEU):*** Wir empfehlen, bei Patienten mit Sepsis oder septischem Schock für ein kontinuierliches Nierenersatzverfahren eine mittlere Dosis von 20–25 ml/kg/h über die geplante Behandlungsdauer zu verabreichen (*Empfehlungsgrad: stark).*

**DSG 2025 *(NEU):*** Wir schlagen vor, bei Patienten mit Sepsis oder septischem Schock eine kontinuierliche hochvolumige („high-volume“) Hämofiltration NICHT anzuwenden (*Empfehlungsgrad: schwach).*

**DSG 2018:** keine Empfehlung.

## Weitere Maßnahmen

### Kortikosteroide

**DSG 2025 *(Änderung):*** Wir schlagen vor, Hydrocortison mit oder ohne Fludrocortison bei Patienten mit septischem Schock mit anhaltendem Vasopressorbedarf trotz ausreichender Volumengabe einzusetzen (*Empfehlungsgrad: schwach).*

*Bemerkungen:* Die empfohlene Dosierung für Hydrocortison beträgt 200 mg/Tag i.v., für Fludrocortison 1 × 50 µg/Tag enteral. Als „anhaltender Vasopressorbedarf“ wird die Gabe von Noradrenalin ≥ 0,25 µg ∙ kg^1^ min^−1^ über mindestens vier Stunden bezeichnet.

**DSG 2018: **Wir schlagen vor, dass von einer Verwendung von intravenösem Hydrocortison bei der Behandlung von Patienten mit septischem Schock abgesehen wird, sofern eine adäquate Flüssigkeitstherapie und hoch dosierte Vasopressor-Therapie in der Lage sind, die hämodynamische Stabilität wiederherzustellen. Für den Fall, dass dies nicht erreichbar sein sollte, schlagen wir den Einsatz von intravenösem Hydrocortison bei einer Dosis von 200 mg pro Tag vor (*Empfehlungsgrad: schwach).*

### Blutreinigung – unselektive Adsorptionsverfahren

**DSG 2025 *(NEU):*** Wir empfehlen, dass bei Patienten mit Sepsis oder septischem Schock unselektive Adsorptionsverfahren NICHT eingesetzt werden (*Empfehlungsgrad: stark).*

**DSG 2018: **keine Empfehlung.

### Blutreinigung – Polymyxin-B-Hämoperfusion

**DSG 2025 *(NEU):*** Wir können bei Patienten mit Sepsis oder septischem Schock weder für noch gegen den Einsatz einer Polymyxin-B-Hämoperfusion eine Empfehlung aussprechen (*Expertenkonsens).*

**DSG 2018: **keine Empfehlung.

### Blutreinigung – Plasmapherese

**DSG 2025 *(NEU):*** Wir können bei Patienten mit Sepsis oder septischem Schock weder für noch gegen den Einsatz einer Plasmapherese eine Empfehlung aussprechen (*Expertenkonsens).*

**DSG 2018: **Wir empfehlen, dass von Blutreinigungstechniken außerhalb von klinischen Studien abgesehen wird (*Expertenkonsens).*

### Antikoagulation und Thromboseprophylaxe

**DSG 2025 *(Änderung):*** Wir empfehlen bei Patienten mit Sepsis oder septischem Schock eine pharmakologische Prophylaxe einer venösen Thromboembolie (VTE) mittels niedermolekularen Heparins (NMH) (*Empfehlungsgrad: stark).*

**DSG 2018:** Wir empfehlen eine pharmakologische Prophylaxe einer venösen Thromboembolie (VTE) mittels unfraktioniertem Heparin (UFH) oder niedermolekularem Heparin (NMH), sofern keine Kontraindikationen in Bezug auf die Verwendung dieser Wirkstoffe vorliegen (*Empfehlungsgrad: stark)*

**DSG 2018:** Wir geben keine Empfehlung für die Bevorzugung von NMH gegenüber UFH zur VTE-Prophylaxe ab (*Statement).*

**DSG 2025 *(NEU):*** Wir schlagen vor, bei hochgradig eingeschränkter Nierenfunktion und/oder septischem Schock mit hohem Vasopressorbedarf entweder niedermolekulares Heparin (NMH) unter engmaschigen Anti-Xa-Kontrollen oder unfraktioniertes Heparin (UFH) einzusetzen (*Expertenkonsens).*

**DSG 2018: **keine Empfehlung.

### Glukosemanagement

**DSG 2025 *(Änderung):*** Wir empfehlen bei Patienten mit Sepsis oder septischem Schock den Beginn einer Insulintherapie bei einem oberen Blutzuckerspiegel von ≥ 180 mg/dl (≥ 10 mmol/l) (*Empfehlungsgrad: stark).*

**DSG 2018:** Wir empfehlen einen protokollierten Ansatz für das Blutzuckermanagement bei ITS-Patienten mit Sepsis, bei denen die Insulindosisgabe beginnt, wenn zwei aufeinanderfolgende Messungen einen Blutzuckerspiegel von > 180 mg/dl ergeben. Bei diesem Ansatz sollte bevorzugt ein oberer Blutzuckerspiegel von ≤ 180 mg/dl statt eines oberen Zielblutzuckerspiegels von ≤ 110 mg/dl angestrebt werden (*Empfehlungsgrad: stark).*

### Stressulkusprophylaxe

**DSG 2025 *(NEU):*** Wir empfehlen, dass Patienten mit Sepsis oder septischem Schock mit vorliegenden Risikofaktoren für gastrointestinale (GI-)Blutungen eine Stressulkusprophylaxe mit Protonenpumpeninhibitoren erhalten (*Empfehlungsgrad: stark).*

**DSG 2018: **Wir schlagen vor, dass entweder Protonenpumpenhemmer oder Histamin-2-Rezeptor-Antagonisten verwendet werden, wenn eine Indikation für eine Stressulkusprophylaxe vorliegt (*Empfehlungsgrad: schwach).*

### Vitamine

**DSG 2025 *(NEU):*** Wir empfehlen, bei Patienten mit Sepsis oder septischem Schock hochdosiertes Vitamin C – weder als Monotherapie noch als Kombinationstherapie mit Hydrocortison und hochdosiertem Vitamin B1 (Thiamin) – NICHT zu verwenden (*Empfehlungsgrad: schwach).*

**DSG 2018:** keine Empfehlung

Behandlungsziele und Spätfolgen

Behandlungsziele und Palliativversorgung

**DSG 2025 *(NEU):*** Wir schlagen vor, bei Patienten mit Sepsis oder septischem Schock, die Behandlungsziele möglichst früh (innerhalb von 72 h) festzulegen (*Expertenkonsens).*

**DSG 2018: **keine Empfehlung.

### Entlassmanagement

**DSG 2025 *(NEU):*** Wir empfehlen, dass Sepsis-Überlebende und deren Angehörige über das Beratungsangebot von Selbsthilfe-Gruppen informiert werden (*Expertenkonsens).*

**DSG 2018: **keine Empfehlung.

**DSG 2025 *(NEU):*** Wir empfehlen, dass bei Sepsis-Überlebenden im Entlassbericht auf die Möglichkeit der Entwicklung eines Post-Intensive-Care-Syndroms (PICS) hingewiesen wird (*Expertenkonsens).*

**DSG 2018: **keine Empfehlung.

## 1. Geltungsbereich und Zweck

### 1.1 Zielsetzung und Fragestellung

Das Institut für Qualitätssicherung und Transparenz im Gesundheitswesen (IQTIG) hat im Rahmen der Erstellung einer Konzeptstudie zu einem Qualitätssicherungsverfahren Sepsis umfangreiche empirische Prüfungen anhand von Sozialdaten der Krankenkassen (gemäß § 299 Abs. 1a SGB V) durchgeführt und 2020 publiziert [7]. Danach werden jährlich derzeit in Deutschland 91.000 Patienten mit Sepsis oder septischem Schock stationär behandelt, die mittlere Verweildauer beträgt im Mittel 25 (median 17 Tage), und die Krankenhaussterblichkeit 38,4 % (55,1 % bei septischem Schock). Aufgrund der demografischen Entwicklung und der zunehmenden Fortschritte der Hochleistungsmedizin ist von einer Zunahme der Sepsisfälle auszugehen. Im Rahmen des Innovationsfonds-geförderten Projektes OPTIMISE wurde zudem festgestellt, dass Sepsis nicht korrekt über ICD-Codes in Abrechnungsdaten abgebildet wird [8]. Insbesondere unterscheiden sich Krankenhäuser deutlich in der Validität der Sepsiskodierung. Hierdurch kommt es zu starken Verzerrungen bei der Berechnung von Qualitätsindikatoren der Sepsisversorgung auf Basis von Abrechnungsdaten. Um diesem Problem zu begegnen, wurde durch eine Expertengruppe des Deutschen Qualitätsbündnisses Sepsis (DQS) in Zusammenarbeit mit der Deutschen Sepsis-Gesellschaft ein umfänglicher Kodierleitfaden für die Sepsis entwickelt, welcher die aktuellen klinischen Definitionen, ICD-Codes sowie Kodierrichtlinien berücksichtigt [9].

Wie kaum eine andere Erkrankung stellt die Sepsis eine interdisziplinäre Herausforderung dar, da sie in allen Fachdisziplinen der Medizin – wenn auch mit unterschiedlicher Häufigkeit – prävalent ist und eine anhaltend hohe Sterblichkeit und Morbidität hat. Kenntnisse in der evidenzbasierten Diagnostik und Therapie der zugrunde liegenden Infektion und des Sepsis-assoziierten Organversagens sind elementarer Wissensbestandteil von Medizinern und Pflegepersonal. Die S3-Leitlinie Sepsis wird daher von Rezipienten fast aller medizinischen Fachbereiche mit hohem Interesse aufgenommen. Aufgrund von Erfahrungen der Vergangenheit ist davon auszugehen, dass das Update dieser Leitlinie auf Fort- und Weiterbildungsveranstaltungen fast aller Fachdisziplinen in Deutschland weite Verbreitung findet und Gegenstand zahlreicher Publikationen in den Organen der einzelnen wissenschaftlich-medizinischen Fachgesellschaften, aber auch in den Pflegewissenschaften sein wird.

Auch für das vom G‑BA beauftragte Qualitätssicherungsverfahren (QS-Verfahren) zur Diagnostik, Therapie und Nachsorge der Sepsis wird die Leitlinie wichtige Handlungsempfehlungen zur Verfügung stellen können. Sie liefert auch die Grundlage für die Lehre an deutschen Universitätsklinika und wird diesbezüglich in den Nationalen Kompetenzbasierten Lernzielkatalog NKLM einfließen.

Die Leitlinie bezieht sich auf erwachsene (> 18 Jahre) Patienten mit Sepsis oder septischem Schock. Sie bietet eine Informationsgrundlage zu angemessenen, wissenschaftlich begründeten und aktuellen Verfahren für Diagnostik, Therapie und Nachsorge und beinhaltet Empfehlungen zur Prävention der Sepsis.

### 1.2 Versorgungsbereich

Diese Leitlinie gilt für alle Versorgungsbereiche. Aspekte der Prävention, Früherkennung, Diagnostik und Nachsorge sind insbesondere für die Primärversorgung und Rehabilitation relevant. Aspekte der Therapie insbesondere für die Akutversorgung.

### 1.3 Patient*innenzielgruppe

Diese Leitlinie bezieht sich auf erwachsene Patientinnen und Patienten mit Sepsis oder septischem Schock.

### 1.4 Adressaten

Die interdisziplinäre Leitlinie der Qualität S3 zur Prävention, Diagnose, Therapie und Nachsorge der Sepsis richtet sich mit Fokus auf die Intensivmedizin und Infektiologie an Anästhesisten, Intensivmediziner, Notfallmediziner, Ernährungsmediziner, Neurologen, Kardiologen, Internisten, Infektiologen, Nephrologen, Pneumologen, Chirurgen, Hygieniker und Mikrobiologen sowie Patienten und Selbsthilfegruppen. Sie dient auch zur Information für die Pflegeberufe und übergeordnete Organisationen (z. B. Krankenkassen und Einrichtungen der ärztlichen Selbstverwaltung) und die interessierte Fachöffentlichkeit.

### 1.5 Weitere Dokumente zu dieser Leitlinie

Weitere Dokumente zur Langfassung der Leitlinie sind auf der Website der AWMF: https://register.awmf.org/de/leitlinien/detail/079-001 frei verfügbar:

- Kurzfassung – eine Übersicht aller abgestimmten Empfehlungen und Statements sowie der wesentlichen Tabellen der Langfassung,
- Update 2025: was ist neu? – eine verkürzte übersichtliche Darstellung aller Empfehlungsänderungen gegenüber der Vorgänger-Version der Leitlinie DSG 2018,
- Leitlinienreport – detaillierte Beschreibung des methodischen Vorgehens bei der Aktualisierung der Leitlinie, die Beschreibung des Umgangs mit Interessenkonflikten und Tabelle der Interessenerklärungen der Leitliniengruppe, Evidenzprofile (GRADE) zu allen evidenzbasierten Empfehlungen sowie Ergebnisse und Dokumentation der systematischen Recherche und methodischen Bewertung der Literatur.

## 2. Screening und Erstmaßnahmen

### 2.1 Einleitung

Bei einer Sepsis handelt es sich um eine lebensbedrohliche Organdysfunktion, ausgelöst durch eine Infektion, welche mit einer Regulationsstörung beim Wirt einhergeht [10]. Somit wird für die Diagnose einer Sepsis dezidiert das Vorliegen einer oder mehrerer Organdysfunktion(en) gefordert. Die Kodierung einer Sepsis im ICD-10 GM 2023 erfolgt mittels eines Codes für die Sepsis, z. B. A 41.– („Sonstige Sepsis“). Für die Kodierung der Organkomplikation(en) können z. B. eine oder mehrere der unten aufgeführten Schlüsselnummern verwendet werden [9]:

- R 57.2 – septischer Schock
- N 17. – akutes Nierenversagen
- J 96.0 – akutes respiratorisches Versagen
- D 65 – disseminierte intravasale Gerinnungsstörung
- G 93.4 – Enzephalopathie, nicht näher bezeichnet
- K 72.0 – Leberversagen

Das frühzeitige Erkennen einer Sepsis und eine frühzeitige evidenzbasierte Behandlung tragen entscheidend dazu bei, die Erkrankungsschwere und das Überleben zu verbessern.

Die Leitlinie fasst angemessene, wissenschaftlich begründete und aktuelle Verfahren für Diagnostik, Therapie und Nachsorge zusammen und ergänzt diese um Maßnahmen der Sepsis-Prävention. Die Empfehlungen der Leitlinie sollen als unterstützende Informations- und Entscheidungsgrundlage für den Kliniker zur Behandlung von erwachsenen Patienten mit Sepsis oder septischem Schock dienen. Sie ersetzen nicht die Fähigkeiten des Arztes, eine angemessene Entscheidung bei der individuellen Behandlung eines Patienten nach Maßgabe der verfügbaren klinischen Parameter zu treffen.

Die vorliegende interdisziplinäre Leitlinie der Klassifikation S3 ist ein evidenz- und konsensbasiertes Instrument zur Verbesserung und Qualitätssicherung von Prävention, Diagnostik, Therapie und Nachsorge der Sepsis. Sie richtet sich mit dem Fokus auf die Querschnittsfächer Intensiv- und Infektionsmedizin an Mitglieder unterschiedlicher Fachgesellschaften.

### 2.2 Definition der Sepsis

Die dieser Leitlinie zugrunde liegende Definition der Sepsis basiert auf den von der Sepsis-3-Task-Force veröffentlichten Definitionen [10]. Sie wird in der vorliegenden Leitlinie nicht als Empfehlung, sondern als Statement (Stellungnahme) beschrieben. Studien, die als Evidenz für die SSC-Leitlinie und die vorliegende Leitlinie herangezogen wurden, beziehen sich allerdings zu einem großen Teil auf Patientenpopulationen, die anhand überholter Definitionen der Sepsis, schweren Sepsis und des septischen Schocks identifiziert wurden.

#### Definition 1/Statement/Bestätigt (Stand 2025)

- Eine Sepsis ist eine akut lebensbedrohliche Organdysfunktion, hervorgerufen durch eine inadäquate Wirtsantwort auf eine Infektion. Für die Diagnose einer Sepsis-assoziierten Organdysfunktion ist ein Anstieg des Sequential Organ Failure Assessment (SOFA) Score um ≥ 2 Punkte zu verwenden.
- Zusätzliches DSG-Leitlinienstatement
- Literatur: [11]
- Konsensstärke: 100 %

Die überholten Sepsis-Definitionen (Sepsis‑1 von 1992 und Sepsis‑2 von 2001) beruhten auf dem SIRS-Konzept (Systemisches inflammatorisches Response-Syndrom) [12, 13]. Die SIRS-Kriterien, die eine Hypo- (≤ 36 °C) oder Hyperthermie (≥ 38 °C), Tachykardie (≥ 90/min), Tachypnoe (≥ 20/min) sowie eine Leukozytose ≥ 12.000/µl oder Leukopenie ≤ 4000/µl und/oder Linksverschiebung > 10 % umfassen, sind weder spezifisch noch besonders sensitiv für Infektionen. So berichteten Churpek et al. [14], dass 50 % der Krankenhauspatienten SIRS mindestens einmal während ihres Krankenhausaufenthaltes aufwiesen, auch wenn viele dieser Patienten keine Infektion hatten und keine antiinfektive Therapie benötigten [14]. Kaukonen et al. [15] haben gezeigt, dass einer von acht Intensivpatienten mit Infektionsverdacht und neu aufgetretenem Organversagen weniger als zwei SIRS-Kriterien erfüllt [15]. Diese Patientengruppe wurde gleichwohl als „septisch“ eingestuft und entsprechend behandelt. Epidemiologische Analysen von Krankenhausdaten, welche bei der Erfüllung von nur zwei SIRS-Kriterien bereits eine Kodierung als Sepsis gestatteten, haben somit zu einer „Verwässerung“ der realen Häufigkeit von und der Sterblichkeit durch Sepsis geführt. So konnten Gaieski et al. [16] für die USA zeigen, dass die relative Krankenhaussterblichkeit in den Jahren 2004–2009 zwar zurückging, die absolute Sterblichkeit aber kontinuierlich stieg [16].

Als Reaktion auf diese Inkonsistenzen erarbeitete die Sepsis-3-Task-Force, eine internationale Arbeitsgruppe der European Society of Intensive Care Medicine (ESICM) und der Society of Critical Care Medicine (SCCM), im Februar 2016 eine Neudefinition der Sepsis [10, 11].

Erstmals wurde die Sepsis im gesamten Krankenhaus, also auch auf Normalstationen und in Notaufnahmen, berücksichtigt. Sepsis wird demnach immer durch eine akut lebensbedrohliche, weil dysregulierte Wirtsreaktion (Organdysfunktion) auf eine Infektion verursacht. Der Begriff der „schweren“ Sepsis entfällt, weil es eine „leichte“ Sepsis in dem neuen Konzept nicht gibt. Die Autoren schlugen vor, stattdessen folgerichtig von einer „Infektion“ zu sprechen. Basierend auf routinemäßig erhobenen klinischen Daten identifizierten die Autoren robuste Risikofaktoren, welche über das Risiko zur Baseline hinaus den Übergang von einer lokal begrenzten Infektion mit geringem Sterberisiko zu einer Sepsis mit hohem Sterberisiko anzeigen. Hierzu waren geeignete statistische Verfahren erforderlich, da es gegenwärtig keinen Goldstandard für die Diagnose einer Sepsis gibt. Nur wenn ein Goldstandard existiert, ist die Berechnung von Sensitivität und Spezifität auf Grundlage folgender Zuordnungen möglich: richtig-positive (Fälle mit Sepsis), richtig-negative (Kontrollen ohne Sepsis) bzw. falsch-positive und falsch-negative. Stattdessen musste die Aussagekraft eines Tests unter verschiedenen Aspekten der Validität, Reliabilität und Nützlichkeit beurteilt werden.

Für die Feststellung der Sepsis-assoziierten Organdysfunktion wird eine Veränderung des SOFA-Scores um ≥ 2 Punkte vorgeschlagen. Ein SOFA-Score von ≥ 2 entspricht einem Mortalitätsrisiko von über 10 % bei stationären Patienten außerhalb der Intensivstation (ITS), die eine Infektion haben. Der SOFA-Score, der sechs Organsysteme nach vier Schweregraden der Organdysfunktion einstuft und 0 bis 24 Punkte umfasst, ist jedoch aufwendig zu ermitteln und daher in der klinischen Routine außerhalb der ITS zum bettseitigen Screening ungeeignet. Daher hat die Sepsis-3-Arbeitsgruppe den qSOFA entwickelt, um Patienten mit hohem Sepsis-Risiko schneller zu identifizieren.

#### Definition 2/Statement/Bestätigt (Stand 2025)

- Ein septischer Schock ist definiert als eine trotz adäquater Volumentherapie persistierende arterielle Hypotension mit der Notwendigkeit einer Therapie mit Vasopressoren, um einen mittleren arteriellen Blutdruck von ≥ 65 mm Hg zu erreichen. Gleichzeitig muss der Laktatwert im Blut > 2 mmol/l betragen.
- Zusätzliches DSG-Leitlinienstatement
- Literatur: [11]
- Konsensstärke: 100 %

Ein systematisches Review, das die Operationalisierung von gegenwärtigen Definitionen des septischen Schocks untersuchte, zeigte eine erhebliche Heterogenität der berichteten Sterblichkeitsraten auf [17]. Diese Heterogenität resultierte u. a. aus Unterschieden in den ausgewählten klinischen Variablen (unterschiedliche Cut-offs für den systolischen oder mittleren Blutdruck, unterschiedliche Level von Hyperlaktatämie und Vasopressor-Dosis). Unter Verwendung der o. g. Definition (mittlerer arterieller Blutdruck von ≥ 65 mm Hg UND Laktatwert > 2 mmol/l) wurden in großen klinischen Kohorten, Notaufnahmen und Normalstationen Krankenhaussterblichkeitsraten von mehr als 40 % festgestellt. Durch die Laktatmessung mittels Point-of-Care-Geräten kann Zeit eingespart werden, die Werte im Vollblut korrelieren sehr gut mit dem Serumlaktat [18].

### 2.3 Screening

#### 2.1/Empfehlung/Modifiziert (Stand 2025)

- Empfehlungsgrad: Stark
- Wir empfehlen Krankenhäusern ein Qualitätssicherungsprogramm zum Thema Sepsis, einschließlich eines Sepsis-Screenings für akut erkrankte Hochrisikopatienten und Standardarbeitsanweisungen (SOPs) für die Behandlung.
- SSC-Leitlinienadoption
- Qualität der Evidenz:
- Sepsis-Screening: Moderat ⊕⊕⊕⊝
- SOP: Sehr niedrig ⊕⊝⊝⊝
- Literatur: [19, 20]
- Konsensstärke: 100 %

Qualitätssicherungsprogramme können die Einhaltung von Sepsis-Protokollen optimieren und damit zu verbesserten Behandlungsergebnissen führen [21]. Diese Programme sollten Screening-Tools, Standardarbeitsanweisungen (SOPs) und Aufklärungsprogramme zur Sensibilisierung der Sepsis-Behandlung beinhalten. Die frühzeitige Identifizierung von Patienten mit Sepsis ist von hoher Bedeutung, da eine unverzügliche Therapie die Überlebenschancen wesentlich verbessern und Folgeschäden reduzieren kann [19, 22]. Strategien zur Qualitätssicherung fußen im Idealfall auf einer interdisziplinären Kooperation zwischen allen Fachgebieten.

Eine Metaanalyse von 50 Observationsstudien hat gezeigt, dass Programme zur Qualitätssicherung in der Versorgung von Sepsis-Patienten mit einer signifikant verbesserten Compliance mit den Leitlinienempfehlungen der SSC und einer geringeren Krankenhausmortalität verbunden waren (OR 0,66; 95 % CI 0,61 bis 0,72) [20].

Im Rahmen einer retrospektiven Kohortenstudie von 1.012.410 Sepsis-Aufnahmen in 509 US-Krankenhäusern wurde die Sterblichkeit, vor und nach Einführung der Sepsis-Vorschriften des Bundesstaates New York, mit einer Kontrollpopulation aus anderen Bundesstaaten untersucht [23]. Die Einführung von Sepsis-Vorschriften war dabei mit einem stärkeren Rückgang der Sepsis-Sterblichkeit assoziiert.

Die bisher größte Studie, welche die Beziehung zwischen der Compliance mit den SSC-Leitlinien und der Krankenhausmortalität untersuchte, beinhaltete 29.470 Patienten in 218 Krankenhäusern in den USA, Europa und Südamerika über einen Zeitraum von 7,5 Jahren [24]. Die Krankenhausmortalität verringerte sich um 0,7 % für alle 3 Monate, in denen ein Krankenhaus die SSC-Leitlinien umsetzte, was mit einem Rückgang der Aufenthaltsdauer auf der ITS um 4 % für jede 10 %ige Verbesserung der Compliance in Bezug auf die Empfehlungen verbunden war. Dieser Nutzen wurde zudem in einem breiten geografischen Spektrum nachgewiesen. Eine Studie an 1794 Patienten aus 62 Ländern mit Sepsis bzw. septischem Schock zeigte eine Reduzierung der Wahrscheinlichkeit, im Krankenhaus zu versterben, in Höhe von 36 % bis 40 % im Falle einer Compliance mit den SSC-Leitlinien auf [25].

Die spezifischen einzelnen Maßnahmen, die zu dieser Verbesserung beitrugen, variierten allerdings signifikant zwischen den verschiedenen Studien. Somit kann keine einzelne Maßnahme explizit empfohlen werden.

#### 2.2/Empfehlung/Modifiziert (Stand 2025)

- Empfehlungsgrad: Stark
- Wir empfehlen bei Patienten außerhalb der Intensivstation, bei denen der Verdacht auf eine Infektion besteht, regelmäßig ein geeignetes Screeninginstrument anzuwenden, um eine Sepsis frühzeitig zu erkennen.
- Zusätzliche DSG-Leitlinienempfehlung/Aktualisierung der Evidenz
- Qualität der Evidenz:
- Mortalität: Sehr niedrig ⊕⊝⊝⊝
- Literatur: [22]
- Konsensstärke: 100 %

Die frühzeitige Identifizierung von Patienten mit Sepsis ist von hoher Bedeutung, da eine frühzeitige Therapie das Überleben wesentlich verbessern kann. Allerdings ist die Symptomatik oft sehr unspezifisch und heterogen. Patienten haben oft allgemeine Anzeichen und Symptome einer Infektion (z. B. Fieber oder Hypothermie) und Symptome, die spezifisch für den Ort der Infektion sind (z. B. Husten, Dysurie oder Erythem), sowie Symptome einer akuten Organdysfunktion (z. B. Verwirrtheitszustände, Oligurie oder Dyspnoe) [26]. Im Gegensatz zu anderen akut lebensbedrohlichen Erkrankungen (wie z. B. dem Herzinfarkt) stehen jedoch derzeit keine diagnostischen bettseitigen Kriterien zur Verfügung, um Patienten mit Sepsis bzw. solche mit hohem Risiko für die Entwicklung einer Sepsis zuverlässig zu identifizieren. Sepsis-Screening-Tools dienen der Früherkennung einer Sepsis und der Identifikation von Patienten mit einem hohen Risiko, an einer Sepsis zu sterben.

In einer aktuellen Metaanalyse, die 22 Studien mit 19.580 Patienten analysierte, waren Sepsis-Screening-Tools in der Notaufnahme mit einer besseren Einhaltung der Sepsis-Bündel, einer geringeren Sterblichkeit und einem kürzeren Krankenhausaufenthalt verbunden [22]. Die bislang größte Studie zum Sepsis-Screening zeigte bei Krankenhauspatienten auf Normalstation eine signifikant reduzierte 90-Tage-Sterblichkeit durch ein regelmäßiges elektronisches Sepsis-Screening [27].

Es existieren verschiedene Instrumente, die bezüglich ihrer Eignung zum Erkennen einer Sepsis oder zur Vorhersage der Prognose untersucht wurden. Beispiele sind: die Systemic-Inflammatory-Response-Syndrome(SIRS)-Kriterien, der quick Sequential Organ Failure Assessment (qSOFA) Score, der Modified Early Warning Score (MEWS) oder der National Early Warning Score (NEWS). In Großbritannien wird aktuell schon der überarbeitete NEWS2 eingesetzt, welcher u. a. auch über einen eigenen Abschnitt (SpO_2_-Skala 2) für die Anwendung bei Patienten mit gesichertem hyperkapnischem Versagen mit entsprechend adaptierten SpO_2_-Werten verfügt (Tab. 1).

**Tab. 1:** Häufig verwendete Sepsis-Screening-Tools

	qSOFA	SIRS	MEWS	NEWS/NEWS2
Temperatur	–	*✓*	*✓*	*✓*
Herzfrequenz	–	*✓*	*✓*	*✓*
Blutdruck	*✓*	–	*✓*	*✓*
Atemfrequenz	*✓*	*✓*	*✓*	*✓*
GCS	*✓*	–	*✓*	*✓*
SpO_2_	–	–	–	*✓*
O_2_-Zufuhr	–	–	–	*✓*
Leukozyten	–	*✓*	–	–

*qSOFA* quick Sequential Organ Failure Assessment (qSOFA), *SIRS* Systemic Inflammatory Response Syndrome, *MEWS* Modified Early Warning Score, *NEWS* National Early Warning Score

Der SOFA-Score wird verwendet, um die für die Sepsis-Definition erforderliche Organdysfunktion (erforderlich ≥ 2 Punkte) zu quantifizieren. Der SOFA-Score, der sechs Organsysteme nach vier Schweregraden der Organdysfunktion einstuft, ist jedoch aufwendig zu ermitteln und daher in der klinischen Routine außerhalb der Intensivstation zum bettseitigen Screening ungeeignet. Daher wurde der qSOFA (eine „schnelle Version“ des SOFA-Scores) entwickelt und in der Vorversion dieser Leitlinie empfohlen, um Patienten mit hohem Sepsis-Risiko außerhalb der Intensivstation frühzeitig zu identifizieren [28]. Der qSOFA mit den drei Variablen veränderter mentaler Status, systolischer RR ≤ 100 mm Hg und Atemfrequenz ≥ 22/min (2 Kriterien = qSOFA positiv) ist einfach anzuwenden und erfordert keine Labortests. Nachfolgende Studien zeigten, dass der qSOFA eine höhere Spezifizität zur frühzeitigen Diagnose einer infektionsbedingten Organdysfunktion hat als die früher verwendeten (zwei der vier) SIRS Kriterien [29, 30]. So war in einer Metaanalyse der qSOFA spezifischer (Spezifität 74,58 %; 95 % CI 73,55 % bis 75,61 %) als die SIRS-Kriterien (Spezifität 35,24 %; 95 %-Kl 22,80 % bis 47,69 %), aber weniger sensitiv (56,39 %; 95 % CI 50,52 % bis 62,27 %) vs. (78,84 %; 95 % CI 74,48 % bis 83,19 %) [30]. In einer großen Validierungsstudie hatten nur 24 % der infizierten Patienten einen qSOFA-Wert von ≥ 2, allerdings machte diese Gruppe 70 % aller Todesfälle aus [10]. Ist der qSOFA-Score positiv (≥ 2), ist eine Sepsis wahrscheinlich – ein negativer qSOFA-Score schließt eine Sepsis jedoch nicht aus. Aufgrund der geringen Sensitivität wurde von der Surviving Sepsis Campaign zuletzt empfohlen, den qSOFA nicht mehr als alleinigen Screeningtest einzusetzen [2].

Early Warning Scores (NEWS, NEWS 2, MEWS) zeigen eine bessere Sensitivität beim Screening auf Sepsis, sind aufgrund der Vielzahl an Parametern allerdings auch etwas aufwendiger zu erheben [31–33].

Insgesamt unterscheiden sich die verfügbaren Screening-Tools durch eine unterschiedliche Sensitivität und Spezifität. Aktuell gibt es keine ausreichende Evidenz, ein einzelnes Screening-Tool aufgrund seiner Überlegenheit zu empfehlen.

Daher erscheint eine Kombination von mehreren Sepsis-Screening-Tools geeignet, um möglichst viele Patienten zu identifizieren [2]. Vorgeschlagen wurde beispielsweise für die Notaufnahme eine Kombination der sensitiven SIRS-Kriterien mit dem spezifischeren qSOFA [34]. Auch die Kombination von qSOFA mit verschiedenen Biomarkern, wie dem Laktatwert, Procalcitonin oder Adrenomedullin (ADM) kann die diagnostische Güte verbessern [35, 36]. Die zusätzliche Anwendung von Laborwerten verkompliziert allerdings den Screeningprozess, die klinischen Vorteile sind bisher nicht ausreichend belegt [37].

Wir schlagen vor, ein Screeninginstrument anzuwenden, dessen Limitationen (hinsichtlich Sensitivität und Spezifität) bekannt sind. Die Kombination von mehreren Sepsis-Screening-Tools oder die Ergänzung mit Laborwerten erscheint vorteilhaft.

Computer-basierte Entscheidungshilfen für frühe Sepsis-Erkennung und KI-Anwendungen scheinen in der diagnostischen Güte (AUROC) den traditionellen Prädiktionstools überlegen zu sein. Aktuell existieren allerdings nur sehr inhomogene Daten, und die klinische Verfügbarkeit der Anwendungen ist sehr gering [38–40].

#### 2.3/Empfehlung/NEU (Stand 2025)

- Empfehlungsgrad: Stark
- Wir empfehlen, bei Erwachsenen mit Verdacht auf Sepsis eine Messung des Laktats im Blut durchzuführen.
- SSC-Leitlinienadaptation/Änderung des Empfehlungsgrades
- Qualität der Evidenz:
- Diagnostische Genauigkeit: Sehr niedrig ⊕⊝⊝⊝
- Literatur: [41–43]
- Konsensstärke: 100 %

Der Zusammenhang zwischen dem Laktatspiegel und der Sterblichkeit bei Patienten mit Verdacht auf Infektion und Sepsis ist gut bekannt [44, 45]. Seine Verwendung wird derzeit als Teil des von der SSC als Teil des 1‑Stunden-Bündels für Patienten mit Sepsis empfohlen [46]; ein erhöhter Laktatwert gehört zudem zur Definition des septischen Schocks [17]. Zur Frage, ob Laktatbestimmungen im Blut auch zum Screening auf das Vorliegen einer Sepsis bei erwachsenen Patienten mit klinisch vermuteter (aber nicht bestätigter) Sepsis verwendet werden können, liegen mehrere Studien vor [41–43].

Die Laktat-Grenzwerte für erhöhte Werte lagen zwischen 1,6 und 2,5 mmol/l, obwohl die diagnostischen Merkmale unabhängig vom Grenzwert ähnlich waren. Die Sensitivität liegt zwischen 66 % und 83 %, die Spezifität zwischen 80 % und 85 %. Zusammenfassend lässt sich sagen, dass das Vorliegen eines erhöhten oder normalen Laktatspiegels die Wahrscheinlichkeit einer endgültigen Sepsis-Diagnose bei Patienten mit Sepsis-Verdacht deutlich erhöht bzw. verringert. Allerdings ist Laktat allein weder sensitiv noch spezifisch genug, um die Diagnose allein zu stellen bzw. auszuschließen. Durch die Laktatmessung mittels Point-of-Care-Geräten kann Zeit eingespart werden, die Werte korrelieren sehr gut mit dem Serumlaktat [18].

### 2.4 Initiale hämodynamische Stabilisierung

#### 2.4/Empfehlung/Bestätigt (Stand 2025)

EK

- Sepsis und septischer Schock sind medizinische Notfälle. Wir empfehlen, dass mit der Behandlung und der hämodynamischen Stabilisierung unverzüglich begonnen wird.
- SSC-Leitlinienadoption
- Konsensstärke: 100 %

Sepsis und septischer Schock sind mit einer hohen Sterblichkeit assoziiert. Neben der frühzeitigen antiinfektiven Therapie und möglichen Fokussanierung ist eine frühzeitige hämodynamische Stabilisierung bei Vorliegen einer Sepsis-induzierten Gewebehypoperfusion oder eines septischen Schocks entscheidend. Eine Sepsis-induzierte Hypoperfusion kann sich durch eine akute Organdysfunktion und/oder eine Verringerung des Blutdrucks sowie eine Erhöhung des Serumlaktats manifestieren. Klinische Hinweise für eine Hypoperfusion können u. a. eine Marmorierung der Extremitäten, Rückgang der Diurese oder eine verlängerte Rekapillarisierungszeit (> 3 s) sein [47, 48].

#### 2.5/Empfehlung/Modifiziert (Stand 2025)

- EK
- Wir schlagen vor, bei Patienten mit Sepsis-induzierter Hypoperfusion oder septischem Schock innerhalb der ersten 3 h als Richtwert 30 mL/kg intravenöse kristalloide Lösung zu verabreichen.
- SSC-Leitlinienadaptation/Änderung des Empfehlungsgrades
- Weiterführende Literatur: [49–51]
- Konsensstärke: 100 %

Als Teil dieser Notfallbehandlung empfehlen wir, bei der initialen Flüssigkeitstherapie mit 30 ml/kg (Standardkörpergewicht) kristalloider Lösung innerhalb der ersten 3 h zu beginnen. Am stärksten scheinen hiervon Patienten mit Hypotonie zu profitieren [52]. Dieses festgelegte Flüssigkeitsvolumen ermöglicht dem Kliniker, die Flüssigkeitstherapie zu initiieren, während er genauere Informationen über den Patienten einholt und auf präzisere Messergebnisse des hämodynamischen Status wartet. Auch bei bestehenden Komorbiditäten wie chronischer Herz- oder terminaler Niereninsuffizienz führte die empfohlene initiale Flüssigkeitsgabe nicht zu vermehrten Komplikationen [53, 54].

Allerdings zeigen Studien, dass die empfohlenen Maßnahmen in Notaufnahmen nur unzureichend erfolgen [55], daher sollten die erforderlichen Maßnahmen, unabhängig vom Behandlungsort, immer kritisch überprüft werden.

Obwohl es in der vorhandenen Literatur nur wenige kontrollierte Daten gibt, welche dieses Flüssigkeitsvolumen unterstützen, haben aktuelle interventionelle Studien dies als die übliche Praxis in den frühen Stadien der Flüssigkeitstherapie beschrieben. Die Evidenz aus Beobachtungsstudien unterstützt diese Praxis [56]. Das durchschnittliche Flüssigkeitsvolumen vor der Randomisierung, welches in der PROCESS-Studie [49], der ARISE-Studie [50] und der PROMISE-Studie [51] verabreicht wurde, lag ebenfalls im Bereich von 30 ml/kg.

Aktuelle RCTs und Metaanalysen weisen darauf hin, dass eine restriktive Flüssigkeitstherapie bei septischem Schock keine Nachteile mit sich bringt [57–59]. Allerdings ist zu beachten, dass dieses Konzept erst nach der initialen Stabilisierungsphase erfolgte. Gleichzeitig war der restriktive Ansatz auch nicht mit Vorteilen bezüglich der Überlebenswahrscheinlichkeit assoziiert. Einige Patienten werden im Verlauf mehr Flüssigkeit benötigen. Diese Flüssigkeitsgabe muss aber immer gegen das Risiko der Flüssigkeitsüberladung abgewogen werden. Eine nicht indizierte liberale Flüssigkeitstherapie kann zu verlängerter Beatmung, Fortschreiten einer akuten Nierenschädigung (AKI) und einer erhöhten Sterblichkeit führen [60]. Daher ist es von zentraler Bedeutung, den Zustand des Patienten mittels sorgfältiger Beurteilung des intravaskulären Volumenstatus und der Organperfusion kontinuierlich neu zu bewerten, um eine Unter- oder Übertherapie mit Flüssigkeit zu vermeiden.

#### 2.6/Empfehlung/Bestätigt (Stand 2025)

- Empfehlungsgrad: Stark
- Wir empfehlen bei Patienten mit septischem Schock, die Vasopressoren benötigen, für den mittleren arteriellen Druck (MAP) einen anfänglichen Zielwert in Höhe von 65 mm Hg (im Vergleich zu höheren Werten).
- SSC-Leitlinienadoption
- Qualität der Evidenz:
- Mortalität: Moderat ⊕⊕⊕⊝
- Literatur: [61]
- Konsensstärke: 100 %

Mittlerer arterieller Druck (Mean Arterial Pressure, MAP) ist der Druck, der die Gewebeperfusion steuert. Obwohl die Perfusion der wichtigsten Organe wie des Gehirns oder der Niere vor einer systemischen Hypotonie durch eine Autoregulation der regionalen Perfusion aufrechterhalten werden kann, wird die Gewebeperfusion unterhalb eines MAP-Schwellenwerts linear von dem arteriellen Druck abhängig.

Eine multizentrische Studie verglich eine Noradrenalin-Dosistitration zum Erreichen eines MAP von 65–70 mm Hg versus 80–85 mm Hg bei Patienten mit septischem Schock [62]. Es lag kein signifikanter Unterschied bezüglich der Mortalität nach Ablauf von 28 Tagen (36,6 % in der Gruppe mit hohem Zielwert und 34,0 % in der Gruppe mit niedrigem Zielwert) oder 90 Tagen (43,8 % in der Gruppe mit hohem Zielwert und 42,3 % in der Gruppe mit niedrigem Zielwert) vor. Die Anvisierung eines MAP von 85 mm Hg führte zu einem signifikant erhöhten Risiko für Arrhythmien, wobei in einer Untergruppe von Patienten mit vorbestehender arterieller Hypertonie ein reduzierter Bedarf für eine Nierenersatztherapie (Renal Replacement Therapy, RRT) bei diesem höheren MAP bestand. Ein kürzlich durchgeführtes RCT verglich bei 2600 Patienten (Alter ≥ 65 Jahre) mit vasodilatorischem Schock eine Gruppe mit „permissiver Hypotonie“ (MAP 60–65 mm Hg) versus Standardtherapie. Die Interventionsgruppe in dieser Studie erreichte einen mittleren MAP von 66,7 mm Hg, verglichen mit 72,6 mm Hg in der Gruppe mit Standardtherapie. In der Interventionsgruppe wurden signifikant weniger Vasopressoren eingesetzt. Die 90-Tage-Mortalität in der Gruppe mit permissiver Hypotonie und der Gruppe mit Standardtherapie war ähnlich (41,0 % versus 43,8 %) [63].

Auch in anderen RCTs wurden die angestrebten Zielblutdruckwerte nicht immer erreicht. So zielte die multizentrische Studie von Asfar et al. [62] darauf ab, Zielblutdruckwerte von 65–70 mm Hg bzw. 80–85 mm Hg zu vergleichen [62]. Tatsächlich verglich die Studie Zielblutdruckwerte von 75 mm Hg bzw. 85 mm Hg.

Eine aktuelle Metaanalyse schloss acht RCTs mit 4561 Patienten ein, in denen bei kritisch kranken Patienten ein hochnormaler oder niedrignormaler MAP-Schwellenwert verglichen wurde [64]. Es fanden sich keine Unterschiede in Bezug auf die Sterblichkeit, günstige neurologische Behandlungsergebnisse oder die Notwendigkeit einer Nierenersatztherapie bei kritisch kranken Patienten, die einem hochnormalen gegenüber einem niedrignormalen MAP-Zielwert zugeordnet wurden.

In Anbetracht der aktuellen Daten wird ein mittlerer arterieller Druck (MAP) von 65 mm Hg bei Patienten mit septischem Schock, die Vasopressoren benötigen, empfohlen. Für höhere MAP-Zielwerte ergeben sich keine klaren Vorteile.

#### 2.7/Empfehlung/NEU (Stand 2025)

- EK
- Wir schlagen vor, bei Patienten mit Sepsis oder septischem Schock Vasopressoren auch periphervenös zu applizieren, um den MAP auf ≥ 65 mm Hg anzuheben und nicht auf die Anlage eines ZVK zu warten.
- SSC-Leitlinienadaptation/Änderung des Empfehlungsgrades und des Empfehlungstextes
- Konsensstärke: 100 %

Die unverzügliche Verabreichung von Vasopressoren zur Wiederherstellung des Blutdrucks ist ein wesentlicher Bestandteil der Behandlung eines septischen Schocks. Vasopressoren werden traditionell über einen zentralvenösen Zugang verabreicht, da bei einer peripheren Verabreichung Paravasate oder eine lokale Ischämie auftreten können. Allerdings kann die Anlage eines zentralvenösen Zugangs zeitaufwendig sein, was zu einer verzögerten Gabe von Vasopressoren führen kann [65]. Große randomisierte Studien, die zentrale und periphere Katheter für die Erstinfusion von Vasopressoren vergleichen, fehlen.

Die Zeit bis zur Einleitung der Vasopressorentherapie kann kürzer sein, wenn ein peripherer Zugang verwendet wird. Eine Post-hoc-Analyse der ARISE-Studie zeigte, dass 42 % der Patienten Vasopressoren über einen peripheren Katheter bekommen hatten. Die Zeit bis zur Gabe der Vasopressoren war in dieser Gruppe signifikant kürzer (2,4 vs. 4,9 h) [65]. Die meisten Patienten, bei denen die Vasopressoren peripher gegeben wurden, hatten innerhalb von einer Stunde einen MAP > 65 mm Hg. Dabei ist eine Verzögerung der Einleitung einer Vasopressorentherapie und des Erreichens eines MAP von 65 mm Hg mit einer erhöhten Sterblichkeit verbunden [66].

Die periphervenöse Vasopressortherapie ist im Allgemeinen sicher. In einem systematischen Review lag die Inzidenz von Paravasaten bei 3,4 % (95 % CI 2,5 % bis 4,7 %), ohne dass Episoden von Gewebsnekrosen oder Extremitätenischämien berichtet wurden [67].

Allerdings sollte die periphervenöse Vasopressortherapie nur für einen kurzen Zeitraum erfolgen, da mit längerer Infusionsdauer Komplikationen zunehmen. Es wird ein Zugang der Größe 20 G oder größer empfohlen, zudem sollte der periphere Venenzugang so proximal wie möglich platziert werden [68].

Zusammenfassend sollte der Ziel-MAP von 65 mm Hg nur so kurz wie möglich unterschritten werden. Daher sollte eine möglichst frühzeitige Gabe von Noradrenalin auch außerhalb der Intensivstation angestrebt werden, um den angestrebten Mitteldruck zu erzielen. Noradrenalin kann in dieser Notfallsituation auch über einen periphervenösen Zugang appliziert werden. Eine invasive Blutdruckmessung sollte im septischen Schock frühzeitig etabliert werden.

### 2.5 Flüssigkeitstherapie

#### 2.8/Empfehlung/Modifiziert (Stand 2025)

- EK
- Wir empfehlen, bei Patienten mit Sepsis oder septischem Schock Flüssigkeitsgaben nach dem initialen Bolus durch wiederholte Kontrollen des hämodynamischen Status zu begleiten und nur fortzusetzen, wenn weiterhin Zeichen einer Hypoperfusion vorliegen.
- Zusätzliche DSG-Leitlinienempfehlung
- Konsensstärke: 100 %

#### 2.9/Empfehlung/NEU (Stand 2025)

- Empfehlungsgrad: Schwach
- Wir schlagen vor, bei Patienten mit Sepsis oder septischem Schock die Flüssigkeitstherapie mit dem Ziel zu steuern, eine erhöhte Laktatkonzentration im Blut zu senken.
- SSC-Leitlinienadoption
- Qualität der Evidenz:
- Mortalität: Niedrig ⊕⊕⊝⊝
- Literatur: [69, 70]
- Konsensstärke: 100 %

#### 2.10/Empfehlung/NEU (Stand 2025)

- Empfehlungsgrad: Schwach
- Wir schlagen vor, bei Patienten mit septischem Schock die Rekapillarisierungszeit zur Beurteilung der Gewebeperfusion zu nutzen.
- SSC-Leitlinienadoption
- Qualität der Evidenz:
- Mortalität: Sehr niedrig ⊕⊝⊝⊝
- Literatur: [70]
- Konsensstärke: 100 %

Die intravenöse Flüssigkeitstherapie ist ein Eckpfeiler der Therapie des septischen Schocks. Eine Minderperfusion der Organgewebe ist eine der pathophysiologischen Ursachen der septischen Organdysfunktion [71–73]. Ziel ist daher, eine Euvolämie mit intravenösen Flüssigkeiten wiederherzustellen, um die Gewebeperfusion in den Organen zu optimieren. Dies sollte in der Initialphase der Versorgung möglichst rasch und anschließend zurückhaltend erfolgen, sobald sich der Patient stabilisiert.

Nach der initialen Volumentherapie sollte die intravenöse Flüssigkeitsgabe nur weiter fortgeführt werden, wenn der Patient Zeichen einer Gewebeminderperfusion zeigt und davon auszugehen ist, dass der Patient von einer weiteren Volumengabe profitieren wird.

Das Serumlaktat ist ein wichtiger Marker für eine Gewebshypoxie, aber kein direkter Maßstab für die Gewebeperfusion. Da es sich bei Laktat um einen Standardlabortest bzw. sogar um einen im Rahmen der Point-of-Care-Blutgasanalyse verfügbaren Wert handelt, kann es als objektiveres Surrogat für die Gewebeperfusion im Vergleich zur körperlichen Untersuchung oder der Urinausscheidung dienen. Erhöhungen der Serumlaktatwerte können durch eine Gewebehypoxie, eine beschleunigte aerobe Glykolyse durch übermäßige betaadrenerge Stimulation oder andere Ursachen bedingt sein (z. B. Leberversagen). Der Serumlaktatspiegel sollte daher im klinischen Kontext und unter Berücksichtigung anderer Ursachen für ein erhöhtes Laktat berücksichtigt werden. Unabhängig von der Ursache sind jedoch erhöhte Laktatwerte mit einem schlechteren Outcome verbunden. Bei Patienten, bei denen erhöhte Laktatwerte infolge einer Gewebehypoperfusion vorliegen, sollte eine Normalisierung der Laktatwerte angestrebt werden [69].

Eine aktuelle Metaanalyse von Yumoto et al. [74] untersuchte verschiedene Perfusionsparameter bei septischem Schock. Dabei waren eine laktatgesteuerte Therapie und die Rekapillarisierungszeit – im Vergleich zur Standardtherapie und zur Therapiesteuerung mittels zentralvenöser Sauerstoffsättigung – signifikant mit einer niedrigeren Kurzzeitmortalität verbunden. Auch in anderen Analysen zeigte sich durch eine Laktat-gesteuerte Therapie eine Senkung der Mortalität und Verkürzung der Beatmungs- und Hospitalisierungsdauer [75, 76].

In der SSC-2021-Leitlinie [2] wurde neu vorgeschlagen, zur ergänzenden Beurteilung der Gewebeperfusion die Rekapillarisierungszeit heranzuziehen (schwache Empfehlung, Evidenz von geringer Qualität) [2]. Diese Empfehlung basiert auf den Ergebnissen einer multizentrischen randomisierten Studie von Hernández et al. 2019 [70], in der 424 Patienten mit septischem Schock mittels Laktatsteuerung oder Rekapillarisierungszeit therapiert wurden [70]. Bis zum Tag 28 starben 34,9 % in der peripheren Perfusionsgruppe und 43,4 % in der Laktatgruppe (Hazard Ratio 0,75; 95 % CI 0,55 bis 1,02; *P* = 0,06; Risikodifferenz −8,5 %; 95 % CI −18,2 % bis 1,2 %). Damit war das Ergebnis nicht statistisch signifikant. Allerdings gab es in der Gruppe mit Steuerung anhand der Rekapillarisierungszeit nach 72 h bessere Werte im SOFA-Score. Trotz fehlender Reduktion der Sterblichkeit wird die Messung der Rekapillarisierungszeit während der initialen Stabilisierung propagiert, da diese leicht durchführbar, nichtinvasiv und kostenfrei ist [70].

#### 2.11/Empfehlung/Bestätigt (Stand 2025)

- Empfehlungsgrad: Schwach
- Wir schlagen vor, bei Patienten mit Sepsis oder septischem Schock bevorzugt dynamische gegenüber statischen Variablen zu verwenden, um eine Flüssigkeitstherapie zu steuern.
- SSC-Leitlinienadoption
- Qualität der Evidenz:
- Mortalität: Sehr niedrig ⊕⊝⊝⊝
- Literatur: [70]
- Konsensstärke: 100 %

Eines der wichtigsten Prinzipien, das in Bezug auf die komplexe Behandlungsplanung dieser Patienten berücksichtigt werden muss, ist die Notwendigkeit einer detaillierten Beurteilung des intravasalen Flüssigkeitsstatus und der Organperfusion mit kontinuierlich zu wiederholenden Kontrollen hinsichtlich des Ansprechens auf die Behandlung.

Eine wiederholte Beurteilung sollte eine gründliche klinische Untersuchung und Bewertung der verfügbaren physiologischen Variablen (Herzfrequenz, Blutdruck, arterielle und zentralvenöse Sauerstoffsättigung, Atemfrequenz, Pulskonturanalyse, Temperatur, Urinausscheidung, Rekapillarisierungszeit und sonstige Variablen je nach Verfügbarkeit) beinhalten sowie weitere nichtinvasive und invasive Überwachungsmaßnahmen, sofern derartige Maßnahmen zur Verfügung stehen.

Frühere Versionen der DSG-Leitlinie enthielten die Empfehlung einer protokollbasierten quantitativen Flüssigkeitstherapie, die auch unter der Bezeichnung „frühzeitige zielgerichtete Therapie (Early Goal-Directed Therapy, EGDT)“ bekannt ist, basierend auf dem von Rivers et al. 2001 [77] publizierten Protokoll. Diese Empfehlung beschrieb die Nutzung einer Reihe von „Zielen“, zu denen u. a. der zentralvenöse Druck (Central Venous Pressure, CVP) und die zentralvenöse Sauerstoffsättigung (Central Venous Oxygen Saturation, ScvO_2_) gehörten. Nachfolgende große multizentrische randomisierte kontrollierte Studien konnten allerdings die positiven Ergebnisse nicht bestätigen [49, 50, 57, 78]. Daher wurden die Empfehlungen zur initialen Stabilisierung revidiert. Der zentrale Venendruck als statischer Vorlastparameter zur Steuerung der Flüssigkeitstherapie wurde aufgegeben, weil die Fähigkeit zur Prognose der Volumenreagibilität limitiert ist, wenn der zentrale Venendruck innerhalb eines relativ normalen Wertebereichs (8–12 mm Hg) liegt.

Dynamische Messungen haben im Vergleich zu statischen Techniken eine bessere diagnostische Genauigkeit bei der Vorhersage der Volumenreagibilität. Diese Techniken umfassen die Schlagvolumenvariation (SVV) oder die Pulsdruckkurvenvariation (PPV) in Reaktion auf Flüssigkeitsbolusgaben oder den passiven Beinhebeversuch (Passive Leg Raising; PLR). Bezüglich einer Reduktion der Mortalität durch dynamische Messungen gab es divergente Ergebnisse [79, 80]. Eine aktuelle Metaanalyse von 7 RCTs, zwei prospektiven und einer retrospektiven Observationsstudie mit insgesamt über 700 Patienten legt einen positiven Effekt des Monitorings der arteriellen Pulskonturanalyse auf Intensivaufenthalt (−3,04 Tage; CI −4,74 bis −1,34), Beatmungsdauer (−1,84 Tage; CI −2,80 bis −0,74) und 28-Tage-Mortalität (RR 0,67; CI 0,48 bis 0,94) nahe [81].

#### 2.12/Empfehlung/Modifiziert (Stand 2025)

- EK
- Wir schlagen vor, bei Patienten mit Sepsis oder septischem Schock eine Echokardiographie zur Beurteilung der Herzfunktion und des Volumenstatus durchzuführen.
- Zusätzliche DSG-Leitlinienempfehlung
- Konsensstärke: 100 %

Ergänzend zur SSC-Leitlinie wird (in Übereinstimmung mit der „S3-Leitlinie zur intravasalen Volumentherapie beim Erwachsenen“ [82]) die Echokardiographie als Methode zur Beurteilung der Herzfunktion explizit aufgeführt. Mittels der Echokardiographie kann beim Patienten mit unklarer hämodynamischer Instabilität eine Vielzahl von relevanten Differenzialdiagnosen (Perikarderguss und -tamponade, akute Rechtsherzbelastung als Hinweis für eine Lungenarterienembolie [LAE], eingeschränkte Pumpfunktion, Klappenvitium etc.) ausgeschlossen werden. Insbesondere bei kardialer Dysfunktion im septischen Schock sind transthorakale Echokardiographie (TTE) und transösophageale Echokardiographie (TEE) essenzieller Bestandteil der Diagnostik. Im Rahmen der Echokardiographie ist für entsprechend erfahrene Untersucher eine Beurteilung des Durchmessers und der Atemvariabilität der V. cava inferior geeignet, zusätzliche Hinweise auf einen Volumenmangel zu liefern [83]. Einschränkend ist darauf hinzuweisen, dass aufgrund der komplexen Kopplung von Makro- und Mikrozirkulation im septischen Schock [70] eine Verbesserung der nutritiven Perfusion klinisch hauptsächlich an der Laktatclearance abgeschätzt wird. Eine „Maximierung“ des Herzzeitvolumens anhand der Frank-Starling-Kurve als Variable (klassische „goal-directed therapy“) führt nicht notwendigerweise zur Normalisierung der Mikrozirkulation und kann zur Volumenüberladung beitragen [84]. Aktuell wird vor diesem Hintergrund eine kombinierte Betrachtung makrohämodynamischer Größen mit klinischen und laborchemischen Zeichen der Verbesserung der mikrovaskulären Perfusion favorisiert [85].

## 3. Infektion und antimikrobielle Therapie

### 3.1 Diagnose der Infektion

#### 3.1/Empfehlung/Bestätigt (Stand 2025)

- EK
- Wir empfehlen, bei Patienten mit Sepsis oder septischem Schock vor Beginn der Antibiotikatherapie mindestens 2 Blutkultur-Sets zu entnehmen. Dies gilt auch für Patienten, die bereits eine antibiotische Vorbehandlung erhalten haben.
- Wie empfehlen die Gewinnung von weiteren Probenmaterialien von möglichen Infektionsfoci (Abstrich, Punktat, Biopsie etc.). Diese darf zu keiner wesentlichen zeitlichen Verzögerung der antiinfektiven Therapie führen.
- Zusätzliche DGS-Leitlinienempfehlung
- Konsensstärke: 100 %

Mikrobiologische Untersuchungen bei Patienten mit Sepsis oder septischem Schock ermöglichen den Nachweis von ursächlichen Infektionserregern und deren Resistenztestung. Die Probennahme orientiert sich am vermuteten Infektionsherd. Bei allen Patienten mit Verdacht auf Sepsis werden *vor* Beginn der antiinfektiven Therapie Blutkulturen zum Nachweis von Bakterien oder Hefepilzen entnommen, da diese Substanzen das Wachstum von Mikroorganismen verlangsamen oder verhindern können [86].

Bei Patienten mit Verdacht auf Sepsis oder septischen Schock erhöht die Entnahme anderer Proben, neben den Blutkulturen, die Wahrscheinlichkeit, den ursächlichen Erreger zu detektieren. Die Entnahme dieser Proben soll ebenfalls vor Beginn der antiinfektiven Therapie erfolgen, sofern dies ohne wesentliche zeitliche Verzögerung möglich ist. Geeignetes Probenmaterial umfasst Urin, Atemwegssekret, Wundsekret, Eiter, Liquor und andere Körperflüssigkeiten. Wenn die Anamnese oder klinische Untersuchung eindeutig auf eine spezifische anatomische Lokalisation des Infektionsherdes hindeutet, sind Materialien von anderen Lokalisationen (mit Ausnahme der Blutkulturen) im Allgemeinen nicht notwendig [87].

Die Nachweisrate bei Blutkulturen steigt mit dem abgenommenen Blutvolumen [88, 89]. Um ein ausreichend großes Blutvolumen zu gewinnen, werden mindestens zwei Blutkultur-Sets benötigt (40–60 ml Blut).

Alle erforderlichen Blutkulturen können zeitgleich aus derselben Punktionsstelle entnommen werden [90–92]. Dies senkt die Kontaminationsgefahr [91]. Eine sequenzielle Abnahme, mehrfache Punktion oder die Abnahme im Temperaturanstieg führen nicht zu einer höheren Nachweisrate [93, 94].

Weitere Empfehlungen zur Präanalytik (Abnahmetechnik, Transportzeit, Lagerung etc.) finden sich in den Mikrobiologisch-Infektiologischen Qualitätsstandards (MIQ) [86].

Bei septischen Patienten mit Verdacht auf eine Katheter-assoziierte Infektion wird mindestens eines der Blutkultur-Sets aus dem Katheter und mindestens eines der Sets aus der peripheren Vene entnommen. Zur weiteren mikrobiologischen Untersuchung wird der Katheter entfernt und die Katheterspitze mikrobiologisch untersucht [95]. Die Abnahme der Blutkulturen aus Katheter und einer peripheren Vene erlaubt die Bestimmung der „Differential Time to Positivity“ (DTP). Diese kann die Diagnose einer Katheter-assoziierten Blutstrom-Infektion stützen, jedoch sind die Daten inkonsistent bezüglich der Vorhersage einer Katheter-assoziierten Infektion [96].

Die Kontamination von Blutkulturen bei der Abnahme ist häufig. Der Zielbereich für die Kontaminationsrate (über alle abgenommenen Blutkulturen hinweg) liegt bei < 3 % [97]. Die Kontaminationsrate sinkt, wenn das Blut bei einer einzelnen Punktion anstatt bei mehrfachen Punktionen entnommen wird [91]. In Studien zur Gewinnung von Blutkulturen konnte zudem gezeigt werden, dass unter Verwendung einer Vorrichtung zur Abzweigung und Verwerfen der ersten Blutportion das Kontaminationsrisiko signifikant reduziert werden konnte [98, 99]. Es ist zu erwarten, dass derselbe Effekt durch Abnahme der ersten Portion in ein anderes Blutröhrchen erzielt werden kann.

Molekularbiologische Nachweisverfahren (z. B. Multiplex-PCR, Next-Generation Sequencing) können die kulturelle Erregerdiagnostik (aus Blut und anderen Probenmaterialien) ergänzen. Der Stellenwert dieser Verfahren ist unklar, da bisher nur begrenzt Daten bezüglich des Einflusses auf das Überleben und die Morbidität vorliegen. Ein Ersatz der kulturellen Erregerdiagnostik durch molekularbiologische Nachweisverfahren ist derzeit nicht sinnvoll, da dadurch auf die Möglichkeit der Erreger-Anzucht und phänotypischer Resistenztestung verzichtet wird.

### 3.2 Beginn der antimikrobiellen Therapie

#### 3.2/Empfehlung/Bestätigt (Stand 2025)

- Empfehlungsgrad: Stark
- Wir empfehlen bei Patienten mit Sepsis, dass die Verabreichung von intravenösen Antiinfektiva so schnell wie möglich, bei Patienten mit einem septischen Schock idealerweise innerhalb einer Stunde erfolgt.
- SSC-Leitlinienadaptation/Änderung des Empfehlungstextes
- Qualität der Evidenz:
- Septischer Schock: Niedrig ⊕⊕⊝⊝
- Sepsis ohne Schock: Sehr niedrig ⊕⊝⊝⊝
- Literatur: [100–102]
- Konsensstärke: 100 %

#### 3.3/Empfehlung/Modifiziert (Stand 2025)

- EK
- Wir schlagen vor, bei einem Verdacht auf eine Sepsis ohne Schocksymptomatik zunächst eine zeitnahe weitere Diagnostik durchzuführen. Falls der Verdacht auf eine zugrunde liegende Infektion weiter besteht, sollte die Verabreichung der antiinfektiven Therapie innerhalb von drei Stunden erfolgen.
- SSC-Leitlinienadaptation/Änderung des Empfehlungsgrades
- Konsensstärke: 100 %

Die frühzeitige Verabreichung geeigneter antiinfektiven Substanzen ist eine der wirksamsten Maßnahmen zur Senkung der Sterblichkeit bei Patienten mit Sepsis [103–105]. Der septische Schock und die Sepsis sind als Notfall einzuschätzen, und die antiinfektive Therapie ist schnellstmöglich zu beginnen (idealerweise innerhalb einer Stunde).

Die Notwendigkeit, eine antiinfektive Therapie so früh wie möglich bereitzustellen, muss jedoch gegen die potenziellen Schäden abgewogen werden, die mit der Verabreichung von unnötigen Antibiotika bei Patienten ohne Infektion verbunden sind [106, 107]. Dazu gehören eine Reihe von unerwünschten Ereignissen wie Allergien oder Überempfindlichkeitsreaktionen, Nierenschäden, Thrombozytopenie, *Clostridioides-difficile*-Infektionen und das Auftreten antimikrobieller Resistenzen [108–113].

Die mit einer frühzeitigen antiinfektiven Behandlung verbundene Senkung der Sterblichkeitsrate scheint bei Patienten mit septischem Schock am ausgeprägtesten zu sein. In einer Reihe von Studien wurde ein starker Zusammenhang zwischen der Zeit bis zum Einsetzen der Antibiotikatherapie und dem Tod bei Patienten mit septischem Schock gefunden, aber ein schwächerer Zusammenhang bei Patienten ohne septischen Schock [100, 101, 114].

In einer Studie mit 49.331 Patienten, die in 149 New Yorker Krankenhäusern behandelt wurden, war jede zusätzliche Stunde, die vom Eintreffen in der Notaufnahme bis zur Verabreichung von Antibiotika verging, mit einer um 4 % erhöhten Wahrscheinlichkeit, im Krankenhaus zu versterben, verbunden (OR 1,04; 95 % CI 1,03 bis 1,06); bei Patienten, die Vasopressoren erhielten, lag die risikoadjustierte OR bei 1,07 (95 % CI 1,05 bis 1,09), bei Patienten, die keine Vasopressoren erhielten bei 1,01 (95 % CI 0,99 bis 1,04) [100].

Eine weitere Studie mit 35.000 Patienten zeigte, dass jede zusätzliche Stunde zwischen dem Eintreffen in der Notaufnahme und der Verabreichung der antiinfektiven Therapie mit einer um 9 % erhöhten Wahrscheinlichkeit, im Krankenhaus zu versterben, verbunden war. Bei Patienten mit „schwerer“ Sepsis (Laktat ≥ 2, mindestens eine Episode von Hypotonie, erforderliche nichtinvasive oder invasive mechanische Beatmung oder Organfunktionsstörungen) lag die Odds Ratio bei 1,07 und bei Patienten mit septischem Schock bei 1,14. Dies entsprach einem absoluten Anstieg der Sterblichkeit um 0,4 % für „schwere“ Sepsis und einem absoluten Anstieg um 1,8 % für septischen Schock [101].

In einer Studie mit 10.811 Patienten aus vier Krankenhäusern in Utah war jede Stunde Verzögerung zwischen dem Eintreffen in der Notaufnahme und der Verabreichung einer antiinfektiven Therapie mit einer um 16 % erhöhten Wahrscheinlichkeit für die Krankenhaus-Sterblichkeit und einer um 10 % erhöhten Wahrscheinlichkeit für die 1‑Jahres-Sterblichkeit verbunden [102]. In anderen Studien wurde jedoch kein Zusammenhang zwischen dem Zeitpunkt der Antiinfektiva-Gabe und der Sterblichkeit festgestellt [115–120].

Es ist zu beachten, dass die genannten Studien Beobachtungsstudien sind, die einem Risiko für Verzerrungen unterliegen. Hauptkritikpunkte sind die Fokussierung auf schwere Erkrankungen (septischer Schock oder intensivmedizinische Behandlung), eine unzureichende Risikoadjustierung bezüglich Komorbiditäten und der Schwere der Erkrankung sowie die unzureichend belegte Annahme, dass der Zusammenhang zwischen Verzögerung der antiinfektiven Therapie und Sterblichkeit linear verläuft und sich folglich eine stündliche Rate errechnen lässt [121]. Weiterhin ist bei Patienten mit Sepsis ohne Schock der Zusammenhang zwischen dem Beginn der antiinfektiven Behandlung innerhalb der ersten Stunden und der Sterblichkeit weniger konsistent [100, 101].

In einem cluster-randomisierten RCT zum Einfluss von Sepsis-Teams und Schulungen auf die Sterblichkeit zeigte sich kein Unterschied im Zeitpunkt der Initiierung der antiinfektiven Therapie zwischen den Studienarmen. Die Wahrscheinlichkeit, innerhalb von 28 Tagen zu versterben, stieg um 2 % je Stunde Zeitverzug in der Verabreichung der antiinfektiven Therapie [122]. Ein zweites RCT zur Gabe von Antibiotika im Rettungswagen ergab keinen signifikanten Unterschied in der Sterblichkeit, obwohl zwischen den Studienarmen ein deutlicher Unterschied von 90 min bei der medianen Zeit bis zur Verabreichung der antiinfektiven Therapie beobachtet wurde [123].

Beobachtungsstudien deuten jedoch darauf hin, dass die Sterblichkeit bei Verzögerung der antiinfektiven Therapie von mehr als 3–5 h nach dem Eintreffen im Krankenhaus und/oder dem Erkennen der Sepsis weiter steigt [100, 102]. In einer aktuellen Metaanalyse von Leung et al. [124] wurden 42 Studien mit 190.896 Sepsis-Patienten untersucht. Die gepoolten Daten zeigten, dass die Odds Ratio für Sterblichkeit von Patienten, die Antibiotika innerhalb von 1 h erhielten, bei 0,83 lag (95 % CI 0,67 bis 1,04) im Vergleich zu Patienten, die Antibiotika nach mehr als 1 h erhielten. Eine signifikante Verringerung des Sterberisikos bei Patienten mit früherer Antibiotikagabe wurde bei den Patientengruppen ≤ 3h gegenüber > 3 h (OR 0,80; 95 % CI 0,68 bis 0,94) und ≤ 6 h gegenüber > 6 h (OR 0,57; 95 % CI 0,39 bis 0,82) beobachtet. Diese Ergebnisse zeigen, dass Antibiotika spätestens innerhalb von 3 h nach Erkennen der Sepsis oder dem Eintreffen in der Notaufnahme verabreicht werden sollten, unabhängig davon, ob ein Schock vorliegt oder nicht [124].

Die Diagnose der Sepsis kann schwierig sein. Symptome, die zunächst wie eine Sepsis aussehen, können sich im weiteren Verlauf als nichtinfektiöse Erkrankungen herausstellen [125–127]. Bei systemischen entzündlichen Reaktionen ohne Infektion ist eine antiinfektive Therapie nicht indiziert. Dazu gehören z. B. eine schwere Pankreatitis oder großflächige Brandwunden. Obwohl die prophylaktische Verwendung systemischer Antibiotika bei einer schweren nekrotisierenden Pankreatitis in der Vergangenheit empfohlen wurde, spricht sich die aktuelle S3-Leitlinie Pankreatitis [128] gegen eine generelle prophylaktische Antibiotikatherapie aus. Bei einem septischen Krankheitsbild und Verdacht auf eine infizierte (peri)pankreatische Nekrose wird eine initiale antiinfektive Therapie mit einem Carbapenem empfohlen [128]. Bei Patienten mit schweren Verbrennungen wurde in der Vergangenheit ebenfalls eine längere systemische antimikrobielle Prophylaxe durchgeführt. Die aktuellen Leitlinien für die Behandlung thermischer Verletzungen des Erwachsenen [129] empfehlen jedoch keine antiinfektiven Prophylaxe bei Patienten mit Verbrennungstrauma, was durch ein Cochrane-Review [130] gestützt wird.

### 3.3 Wahl der antimikrobiellen Therapie

#### 3.4/Empfehlung/Modifiziert (Stand 2025)

- Empfehlungsgrad: Stark
- Wir empfehlen, bei Patienten mit Sepsis oder septischem Schock eine initiale antiinfektive Therapie mit einem Antibiotikum oder mehreren Antibiotika durchzuführen, um alle wesentlichen Bakterien zu erfassen.
- Bemerkung: Die antiinfektive Therapie sollte nach dem vermuteten Infektionsfokus, MRE-Besiedlung und Anamnese ausgerichtet werden.
- Zusätzliche DSG-Leitlinienempfehlung
- Qualität der Evidenz:
- Mortalität: Niedrig ⊕⊕⊝⊝
- Literatur: [131]
- Konsensstärke: 100 %

#### 3.5/Empfehlung/Modifiziert (Stand 2025)

- Empfehlungsgrad: Schwach
- Wir schlagen vor, bei Patienten mit Sepsis oder septischem Schock ohne erhöhtes Risiko für multiresistente Erreger KEINE routinemäßige, initiale Therapie mit zwei verschiedenen gegen gramnegative Erreger wirksamen Substanzen („double gram-negative coverage“) durchzuführen.
- SSC-Leitlinienadaptation/Änderung des Empfehlungstextes
- Qualität der Evidenz:
- Mortalität: Sehr niedrig ⊕⊝⊝⊝
- Literatur: [132]
- Konsensstärke: 100 %

Die Initiierung einer geeigneten antimikrobiellen Therapie, d. h. einer wirksamen Therapie gegen das bzw. die verursachende(n) Pathogen(e), ist einer der wichtigsten Aspekte des effektiven Managements von lebensbedrohlichen Infektionen. Eine unwirksame empirische Therapie bei Patienten mit Sepsis und septischem Schock ist mit einer beträchtlichen Zunahme der Morbidität und Sterblichkeit verbunden [133–135]. Dementsprechend muss die initiale Auswahl einer antiinfektiven Therapie breit genug angelegt sein, um die wahrscheinlichen Pathogene zu erfassen. Im weiteren Verlauf ist anhand von mikrobiologischen Untersuchungsergebnissen eine Umstellung auf eine gezielte antiinfektive Therapie durchzuführen [136].

Die Auswahl der initialen antiinfektiven Therapie hängt von der medizinischen Vorgeschichte, dem klinischen Zustand und lokalen epidemiologischen Faktoren ab. Zu den wichtigsten patientenbezogenen Faktoren gehören: die Lokalisation der Infektion, die zugrunde liegenden Begleiterkrankungen, das Vorliegen einer Immunsuppression, eine kürzlich bekannt gewordene Infektion oder Kolonisierung mit spezifischen Pathogenen sowie eine Behandlung mit Antiinfektiva innerhalb der letzten drei Monate. Außerdem müssen Faktoren wie die Dauer der Hospitalisierung zum Zeitpunkt der Infektion (z. B. ambulant erworben versus nosokomial), die lokale Pathogenprävalenz und die Resistenzmuster dieser Pathogene, sowohl auf regionaler Ebene als auch im Krankenhaus, berücksichtigt werden. Auch Medikamentenunverträglichkeiten, Interaktionen und Toxizität müssen berücksichtigt werden [137].

Die häufigsten Pathogene, die einen septischen Schock verursachen, sind gramnegative und grampositive Bakterien. Bei bestimmten klinischen Konstellationen sind eine invasive Candidiasis, ein toxisches Schocksyndrom und auch selten auftretende Pathogene in Betracht zu ziehen. Weiterhin sind bei Sepsis aufgrund einer ambulant erworbenen Pneumonie neben den typischen Erregern (z. B. Pneumokokken, *Haemophilus influenzae, S. aureus, Enterobacterales*) auch „atypische“ Erreger (Legionellen, Mykoplasmen, Chlamydien und Coxiellen) zu berücksichtigen [138]. Patienten mit Neutropenie haben ein Risiko für ein besonders breites Spektrum an potenziellen Pathogenen, zu denen auch resistente gramnegative Bakterien, Vancomycin-resistente Enterokokken (VRE) und *Candida *spp. gehören.

Angesichts der vielen Faktoren, die bei der Wahl eines geeigneten Antiinfektivums zu berücksichtigen sind, ist die Empfehlung einer universellen antiinfektiven Therapie bei einer Sepsis nicht möglich. In Tab. 2 sind Optionen für eine initiale antiinfektive Therapie aus der S2k-Leitlinie der Paul-Ehrlich-Gesellschaft (PEG) [139] dargestellt. Die vorgeschlagenen Antiinfektiva richten sich nach dem vermuteten Fokus, dem vermuteten Erregerspektrum, dem Ort des Erwerbs der Infektion und dem Schweregrad der Sepsis. Detaillierte Informationen zur Auswahl der Substanzen sowie Dosierungsempfehlungen sind in der Leitlinie der PEG [140] niedergelegt. Es ist zu beachten, dass diese Tabelle einen Expertenkonsens darstellt und nicht immer evidenzbasiert ist. Eine Anpassung an die lokalen Gegebenheiten (Erregerepidemiologie, Resistenzsituation, Medikamenten-Verfügbarkeit) ist notwendig.

**Tab. 2:** Empfehlungen zu Substanzen in der kalkulierten antimikrobiellen Initialtherapie der Sepsis

Fokus	Nosokomial erworben		Ambulant erworben	
	*Septischer Schock*	Sepsis	*Septischer Schock*	Sepsis
Pulmonal	Meropenem oder PipTaz* oder Cefepim ±^1^ Ciprofloxacin oder Levofloxacin^9^	PipTaz oder Cefepim	PipTaz oder Cefepim + Azithromycin oder Moxifloxacin oder Levofloxacin	Cefotaxim oder Ampicillin/Sulbactam + Azithromycin oder Moxifloxacin oder Levofloxacin
Urogenital	PipTaz oder Ciprofloxacin oder Meropenem oder Imipenem ±^1^ Levofloxacin	Cefotaxim oder Cefepim oder PipTaz	PipTaz oder Meropenem oder Imipenem	Cefotaxim oder Cefepim oder PipTaz
Abdominal	PipTaz oder Meropenem oder Imipenem + Linezolid	PipTaz ±^2^ Linezolid alternativ: Tigecyclin + Ceftazidim^8^	PipTaz ±^2, 3^ Linezolid	Cefotaxim + Metronidazol oder PipTaz
Haut/Weichgewebe^5^	PipTaz oder Meropenem + Clindamycin^6^ oder + Linezolid	PipTaz + Clindamycin^6^ oder + Linezolid	PipTaz oder Meropenem + Clindamycin^6^ oder + Linezolid	PipTaz + Clindamycin^6^
Katheter-assoziiert	Daptomycin^7^ + PipTaz oder + Cefepim oder + Meropenem	Daptomycin^7^ ±^4^ Cefepim oder Pip/Taz	–	–
Unbekannt	PipTaz oder Cefepim oder Meropenem^10^ + Linezolid oder + Daptomycin ±^1^ Ciprofloxacin oder Levofloxacin^9^	PipTaz ±^2^ Linezolid	PipTaz oder Cefepim oder Meropenem^10^ ±^2^ Linezolid ±^1^ Ciprofloxacin oder Levofloxacin^9^	PipTaz oder Cefotaxim

^1^: nur bei Risikofaktoren für MRGN addieren

^2^: nur bei Risikofaktoren für MRSA addieren

^3^: bei Risikofaktoren für VRE addieren

^4^: wenn gramnegative Erreger erfasst werden sollen

^5^: Kombinationstherapie insbesondere bei nekrotisierender Fasziitis

^6^: Zur Hemmung der Proteinbiosynthese sollte Linezolid statt Clindamycin bei MRSA-Risiko oder hoher lokaler Clindamycin-Resistenzrate bei A‑Streptokokken verwendet werden

^7^: Alternativ kann Vancomycin (mit Spiegelbestimmung) verwendet werden

^8^: Alternativ kann Eravacyclin + Ceftazidim verwendet werden

^9^: In Abhängigkeit von der lokalen epidemiologischen Situation kann Fosfomycin eine Alternative sein

^10^: Bei Risikofaktoren für 3 MRGN Meropenem bevorzugen

*PipTaz: Piperacillin-Tazobactam

Quelle: adaptiert aus der S2k Leitlinie „Kalkulierte parenterale Initialtherapie bakterieller Erkrankungen bei Erwachsenen“ AWMF Registernummer 082–006 [139] *

*Die Leitlinie war zum Zeitpunkt der Veröffentlichung noch nicht publiziert

Die Gabe eines zweiten Wirkstoff gegen gramnegative Erreger („double gram-negative coverage“) kann die Wahrscheinlichkeit erhöhen, dass zumindest ein aktiver Wirkstoff verabreicht wird [141]. Jedoch zeigte eine Metaanalyse von 10 RCTs keinen Unterschied in der Sterblichkeit zwischen einer Monotherapie und einer Kombination mit einem zweiten Wirkstoff [132]. Dies gilt allerdings nur bei einer niedrigen Prävalenz von multiresistenten Erregern.

Die Ergänzung eines pathogenspezifischen Wirkstoffs zur Erweiterung des Spektrums ist gerechtfertigt, wenn ein spezifisches Risiko besteht. Beispielsweise dient die Hinzugabe eines Makrolids bei Sepsis aufgrund einer ambulant erworbenen Pneumonie zur Erfassung atypischer Erreger. Bei einer nosokomialen Pneumonie hingegen spielen atypische Erreger keine Rolle [142]. Bei bekannter MRSA-Besiedlung oder Risikofaktoren für eine MRSA-Besiedlung ist die antiinfektive Therapie um Daptomycin (nicht bei Pneumonie), Linezolid, Vancomycin, Teicoplanin oder einen anderen Wirkstoff mit Aktivität gegen MRSA zu ergänzen [140].

#### 3.6/Empfehlung/Modifiziert (Stand 2025)

- Empfehlungsgrad: Schwach
- Wir schlagen vor, bei Patienten mit Sepsis oder septischem Schock mit einem niedrigen Risiko für eine Pilzinfektion KEINE antimykotische Therapie durchzuführen.
- Wir schlagen vor, bei Patienten mit Sepsis oder septischem Schock mit einem hohen Risiko für eine Pilzinfektion eine empirische antimykotische Therapie durchzuführen.
- SSC-Leitlinienadoption
- Qualität der Evidenz:
- Für empirische Therapie: Niedrig ⊕⊕⊝⊝
- Für keine empirische Therapie: Niedrig ⊕⊕⊝⊝
- Literatur: [143–149]
- Konsensstärke: 100 %

Durch Pilzinfektionen verursachte Sepsis und septischer Schock sind mit einem schlechten Behandlungsergebnis verbunden [150–153]. Bis zu 7 % der beatmeten kritisch kranken Patienten erhalten eine empirische antimykotische Therapie [154], ohne dass der Nutzen einer empirischen antimykotischen Therapie belegt wurde. In einer randomisierten kontrollierten Studie (EMPIRICUS) [143] wurde die empirische Gabe von Micafungin mit einer Placebo-Gabe bei nichtneutropenischen Patienten mit Sepsis verglichen, und es wurde kein Unterschied bezüglich des ereignisfreien Überlebens festgestellt [143].

Während Patienten mit Sepsis oder septischem Schock im Allgemeinen nicht von einer empirischen antimykotischen Therapie profitieren, könnte dies bei Patienten mit einem hohen Risiko für eine Pilzinfektion der Fall sein (beispielsweise Patienten mit Fieber in Neutropenie und fehlendem Ansprechen auf eine mehrtägige Antibiotikatherapie). Mögliche Risikofaktoren für eine invasive Pilzinfektion sind in Tab. 3 dargestellt, wobei es keinen Konsens gibt, bei welcher Kombination von Risikofaktoren ein hohes Risiko besteht [155]. Hier besteht weiterer Forschungsbedarf.

**Tab. 3:** Risikofaktoren, die Pilzinfektion begünstigen

Risikofaktoren für Candida-Sepsis	Risikofaktor für invasive Schimmelpilzinfektionen
Candida-Besiedlung an mehreren Stellen [163, 164]	Neutropenie [179, 180]
Surrogatmarker wie der Serum-Beta-D-Glucan-Assay [163]	Surrogatmarker wie Serum oder bronchoalveoläre Lavage: Galactomannan-Assay [181]
Neutropenie [165, 166]	Hämatopoetische Stammzelltransplantation [180]
Immunsuppression [167–169]	Organtransplantation [182, 183]
Schwere der Erkrankung (hoher APACHE-Score) [170, 171]	Hochdosierte Kortikosteroidtherapie [184]
Längere Verweildauer auf der Intensivstation [171]	Immun-Modifier [185, 186]
Zentrale Venenkatheter und andere intravaskuläre Devices [171, 172]	Influenza [174]
Drogenabusus (Injektion) [173]	COVID-19 [174]
Vollständige parenterale Ernährung [174]	Moderate bis schwere HIV-Infektion mit CD4-Zellen < 200/µl [174]
Breitbandantibiotika [164, 174]	Solide Tumoren [174]
Perforationen des Gastrointestinaltrakts und Anastomosenlecks [174, 175]	
Gastrointestinale oder hepatobiliäre Notoperationen [175]	
Akutes Nierenversagen und Hämodialyse [175]	
Schwere thermische Verletzungen [176, 177]	
Vorausgegangene Operationen [178]	

Der Beginn einer empirischen Therapie hängt von der Anzahl der Risikofaktoren und der lokalen Epidemiologie ab. Die Gewichtung der Faktoren in Bezug auf das Sepsis-Risiko ist unklar [155].

Quelle: modifiziert nach Evans et al. [2]

Eine von der European Society of Intensive Care Medicine (ESICM) und der European Society of Clinical Microbiology and Infectious Diseases (ESCMID) gebildete Task-Force schlägt eine empirische Therapie ausschließlich bei septischem Schock vor, sofern ein Nachweis einer Candida-Kolonisation an mehr als einer Lokalisation außerhalb des Verdauungstrakts (Urin, Mund, Rachen, unterer und oberer Respirationstrakt, Hautfalten, Drainagen, Operationswunde) vorliegt [156].

Zur Indikation einer empirischen antimykotischen Therapie bei hämatoonkologischen Patienten verweisen wir auf die Empfehlungen der Deutschen Gesellschaft für Hämatologie und Medizinische Onkologie (DGHO) [157]. Bei diesen Patienten ist auch an die Möglichkeit einer Aspergillus-Infektion zu denken. Bei Patienten, die aufgrund einer Influenza oder COVID-19 beatmet werden müssen, kann im Verlauf (auch bei fehlenden klassischen Kriterien für eine Immunsuppression) eine invasive pulmonale Schimmelpilzinfektion auftreten [158, 159].

Die Auswahl des spezifischen Wirkstoffs hat den Schweregrad der Erkrankung und eine eventuelle kürzliche Exposition gegenüber antimykotischen Medikamenten zu berücksichtigen. Bei der kalkulierten antimykotischen Therapie der Sepsis sind Echinocandine zu bevorzugen. Eine Ausnahme besteht bei einem pulmonalen Fokus, bei dem *Candid*a spp. typischerweise keine Rolle spielen und Schimmelpilze vorherrschen. Bei Unverträglichkeit, bekannter Vorbesiedlung mit resistenten Candida-Spezies oder Verdacht auf eine pulmonale Infektion mit „Nicht-Aspergillus-Spezies“ wird dringend ein infektiologisches oder mikrobiologisches Konsil empfohlen [153].

Andere Pilzinfektionen, z. B. durch *Cryptococcus* spp. oder *Pneumocystis jirovecii*, und endemische Mykosen durch dimorphe Pilze (*Histoplasma *spp., *Blastomyces* spp., *Coccidioides* spp., *Paracoccidioides *spp.) spielen bei der Sepsis eine untergeordnete Rolle.

In einer großen Punktprävalenzstudie waren virale Infektionen in unter 4 % Ursache einer Sepsis oder eines septischen Schocks [160]. Eine empirische antivirale Therapie wird aktuell nur bei Verdacht auf eine Influenza-Pneumonie in einer Pandemie-Situation oder bei einer hohen Aktivität der saisonalen Influenza empfohlen [138]. Zur Therapie kritisch kranker Patienten mit SARS-CoV‑2 oder mit Immunsuppression verweisen wir auf die entsprechenden Leitlinien [157, 161, 162].

#### 3.7/Empfehlung/NEU (Stand 2025)

- Empfehlungsgrad: Stark
- Wir empfehlen, bei Patienten mit Sepsis oder septischem Schock Beta-Laktam-Antibiotika als prolongierte oder kontinuierliche Infusion (nach initialem Bolus) zu verabreichen.
- Bemerkung: Bei einer kontinuierlichen Infusion ist auf die physikalisch-chemische Stabilität des Wirkstoffs zu achten.
- SSC-Leitlinienadaptation/Aktualisierung der Evidenz und Änderung des Empfehlungsgrades
- Qualität der Evidenz:
- 90-Tage Mortalität: Hoch ⊕⊕⊕⊕
- ICU Mortalität: Hoch ⊕⊕⊕⊕
- Literatur: [187]
- Konsensstärke: 100 %

Bei den zeitabhängig wirksamen Beta-Laktam-Antibiotika ist für den Therapieerfolg die Zeit entscheidend, in der die freie Konzentration des Antibiotikums (fT) oberhalb der minimalen Hemmkonzentration (MHK) eines Erregers liegt (fT > MHK). Für Penicilline, Cephalosporine und Carbapeneme wurden fT > MHK-Werte von mindestens 50 %, 60–70 % bzw. mindestens 40 % des Dosisintervalls ermittelt. Eine Möglichkeit, die Wirksamkeit von Beta-Laktam-Antibiotika zu verbessern, ist die Verlängerung der Infusionsdauer auf 3–4 h oder die kontinuierliche Infusion über 24 h [188].

Eine aktuelle internationale randomisierte multizentrische klinische Studie hat die Sterblichkeit innerhalb von 90 Tagen untersucht (BLING-III-Studie) [189]. Es wurden 7202 erwachsene Personen mit einer Sepsis unter einer Therapie mit Meropenem oder Piperacillin/Tazobactam in die Studie aufgenommen. Die Teilnehmenden erhielten die Antibiotika entweder als intermittierende Kurzinfusionen (30 min) oder als kontinuierliche Infusion. Der beobachtete Unterschied in der 90-Tage-Sterblichkeit der Patienten mit einer kontinuierlichen (24,9 %) und einer intermittierenden (26,8 %) Infusion der Beta-Laktam-Antibiotika erreichte in der primären Analyse keine statistische Signifikanz (absoluter Unterschied −1,9 %; 95 % CI −4,9 % bis 1,1 %; OR 0,91, 95 % CI 0,81 bis 1,01; *P* = 0,08). Die Rate der klinischen Heilung war bei Patienten mit kontinuierlicher Infusion signifikant besser (55,7 % vs. 50,0 %). Auch in der für Geschlecht und Schweregrad der Erkrankung adjustierten Analyse zeigte sich beim primären Endpunkt ein signifikanter Vorteil.

Eine begleitend publizierte aktualisierte Metaanalyse von 18 randomisierten kontrollierten Studien (17 Studien zu kontinuierlicher Infusion und 1 Studie zu prolongierter Gabe über 3–4 h) verglich eine verlängerte Infusion von Beta-Laktam-Antibiotika mit einer intermittierenden Gabe bei 9108 Patienten mit Sepsis oder septischem Schock. Die 90-Tage-Gesamtsterblichkeit in der Gruppe mit verlängerter Infusion war statistisch signifikant niedriger (RR 0,86; 95 % CI 0,72 bis 0,98 und die Heilungsrate erhöht) [187]. Eine weitere Metaanalyse bestätigte dieses Ergebnis [190].

Daher empfehlen wir, bei Patienten mit Sepsis oder septischem Schock eine verlängerte Infusion von β‑Laktam-Antibiotika bevorzugt durchzuführen. Die Gabe der ersten Dosis als Bolus („loading dose“) dient zum raschen Erreichen eines therapeutischen Wirkspiegels. Unmittelbar im Anschluss an diese Kurzinfusion kann dann eine prolongierte oder kontinuierliche Infusion über 3–4 bzw. 24 h durchgeführt werden. Eine kontinuierliche Applikation ohne regelmäßige und zeitnahe (d. h. Ergebnismitteilung ≤ 24 h) Kontrolle der Blutspiegel (TDM) ist vor allem bei hoher MHK des Erregers und/oder bei gesteigerter Antibiotika-Clearance („augmented renal clearance“ = GFR ≥ 130 ml/min) problematisch, da es eventuell zu einer Unterschreitung der PK/PD-Ziele führt. Inwiefern eine generelle individuelle Steuerung der Dosierung durch ein TDM einen Vorteil für Patienten mit Sepsis bringt, ist nicht geklärt [191]. Generell ist ein TDM jedoch für Substanzen mit einer geringen therapeutischen Breite (z. B. Vancomycin oder Aminoglykoside) und Voriconazol erforderlich.

### 3.4 Dauer der antimikrobiellen Therapie

#### 3.8/Empfehlung/Modifiziert (Stand 2025)

- EK
- Wir empfehlen, bei Patienten mit Sepsis oder septischem Schock die antiinfektive Therapie täglich zu evaluieren und die Therapie anzupassen, sobald ein plausibler Erregernachweis geführt werden konnte und die Antiinfektiva-Empfindlichkeit bekannt ist.
- Zusätzliche DSG-Leitlinienempfehlung
- Konsensstärke: 100 %

Angesichts des potenziellen Schadens, der mit unnötig langen und breit wirksamen antimikrobiellen Therapien verbunden sein kann [192], wird eine tägliche Beurteilung hinsichtlich einer Fokussierung bzw. Beendigung der antimikrobiellen Therapie bei Patienten mit Sepsis oder septischem Schock empfohlen.

Bei Patienten mit Sepsis oder septischem Schock ist initial eine breite antiinfektive Therapie gerechtfertigt, bis der verursachende Erreger und dessen Antiinfektiva-Empfindlichkeit bekannt sind. Sobald ein plausibler Erregernachweis vorliegt, empfehlen wir eine Fokussierung der antiinfektiven Therapie. Dies beinhaltet, alle nichterforderlichen antimikrobiellen Wirkstoffe abzusetzen, Wirkstoffe mit breitem antimikrobiellem Spektrum durch spezifischere Wirkstoffe (Schmalspektrum) und Kombinationstherapien, wenn möglich, durch Monotherapie zu ersetzen [193]. Erregerspezifika wie z. B. für *Staphylococcus-aureus*-Infektionen sind zu beachten [194, 195]. Falls die mikrobiologische Diagnostik keinen oder keinen plausiblen Erregernachweis erbringt, ist eine Fokussierung der Therapie auch basierend auf einem guten klinischen Ansprechen möglich. Grundsätzlich wird die Nutzung von Antimicrobial-Stewardship(AMS)-Programmen zur Optimierung der antiinfektiven Therapie befürwortet [136].

In 12 Beobachtungsstudien [196] und einem RCT [197] mit 1968 Patienten zeigte sich eine geringere Sterblichkeit bei Patienten mit Fokussierung der Therapie (RR 0,72; 95 % CI 0,57 bis 0,91) (eigene Metaanalyse der SSC-Leitlinie 2021 [2]). Es gab keinen Unterschied in Bezug auf das Langzeit-Überleben und die Dauer des Aufenthalts auf der Intensivstation. Die Metaanalyse muss mit Vorsicht interpretiert werden, da vielfältige Verzerrungen möglich sind [2].

Bei 20–30 % der Patienten mit einer Sepsis kann kein verursachendes Pathogen identifiziert werden [133]. Das ist überwiegend dann der Fall, wenn die Materialien für die mikrobiologische Diagnostik unter bereits laufender antimikrobieller Therapie gewonnen wurden. Es kann aber auch darauf zurückzuführen sein, dass keine Infektion vorlag. Falls ein anderer, plausibler und nichtinfektionsbedingter Grund für den klinischen Zustand des Patienten vorhanden ist und hinreichend wahrscheinlich ist, dass keine Infektion vorliegt, besteht keine Indikation, die antiinfektive Therapie fortzusetzen [198].

Eine Fokussierung der antiinfektiven Therapie ist in der Regel sicher (keine erhöhte Sterblichkeit), preiswerter (durch Einsparung unnötiger Antiinfektiva) und führt zu weniger Toxizität [199, 200]. Eine Fokussierung verringert auch das Auftreten von neuen Antibiotikaresistenzen [201].

Eine Anpassung der Antiinfektivatherapie ist nicht automatisch bei jeglichem Nachweis von Mikroorganismen notwendig, sondern nur bei *plausiblem Erregernachweis*. Bei Blutkulturbefunden ist die Abgrenzung einer Infektion von einer Kontamination wichtig, insbesondere bei einmaligem Nachweis von typischen Mikroorganismen des Hautmikrobioms mit geringer Virulenz (z. B. Koagulase-negative Staphylokokken, *Corynebacterium* spp., *Cutibacterium* spp.). Andere Erreger, die auch zum Haut- und Schleimhautmikrobiom gehören (z. B. Enterokokken oder *Candida* spp.), sind hingegen als plausible Erreger zu betrachten, wenn sie aus Blutkulturen oder aus eigentlich sterilen Körperregionen isoliert werden. Ein Nachweis in respiratorischem Material ist hingegen am ehesten Ausdruck einer Kolonisation und nicht einer Infektion. Erreger, die bei Nachweis in der Blutkultur nahezu immer plausible Erreger sind, sind *Staphylococcus aureus, Streptococcus pyogenes* und andere β‑hämolysierende Streptokokken, *Streptococcus pneumoniae*, Enterokokken, *Listeria monocytogenes, Neisseria meningitidis, Haemophilus influenzae, Enterobacterales, Pseudomonas aeruginosa, Acinetobacter baumannii* und *Bacteroides fragilis* [202]. Diese Auflistung erhebt keinen Anspruch auf Vollständigkeit und gibt lediglich eine Orientierung für besonders häufige Erreger.

#### 3.9/Empfehlung/Modifiziert (Stand 2025)

- EK
- Wir empfehlen, bei Patienten mit Sepsis oder septischem Schock eine Antibiotikatherapie in der Regel von 7 bis 10 Tagen durchzuführen. Bei nicht erfolgreicher Fokussanierung, bei bestimmten Erregern sowie speziellen Organinfektionen ist die Therapiedauer zu verlängern.
- Zusätzliche DSG-Leitlinienempfehlung
- Konsensstärke: 94 %

Eine Begrenzung der Therapie auf die kürzest mögliche Dauer ist ein wichtiger Teil des Antimikrobiellen Stewardship [203, 204]. Die optimale Therapiedauer für Patienten mit Sepsis oder septischem Schock hängt von vielen Faktoren ab, darunter Wirtsfaktoren, mikrobielle Faktoren, Eigenschaften der Antiinfektiva und anatomische Lokalisation.

In den letzten Jahren wurden RCTs bei spezifischen Infektionen durchgeführt, um kürzere Therapiedauern mit „traditionellen“ längeren Therapiedauern zu vergleichen, so bei Pneumonie [205], Pyelonephritis [206], Bakteriämie [207] und intraabdominellen Infektionen [208, 209]. In vielen Studien war die kürzere Therapiedauer genauso effektiv wie die längere Therapiedauer, aber mit weniger Nebenwirkungen und Komplikationen assoziiert [204]. Sehr wenige Studien haben sich jedoch ausschließlich auf Patienten mit Sepsis oder septischen Schock beschränkt.

Eine Behandlungsdauer von 7 bis 10 Tagen ist für die meisten schweren Infektionen angemessen. Die aktuelle S3-Leitlinie „Epidemiologie, Diagnostik und Therapie erwachsener Patienten mit nosokomialer Pneumonie“ [210] empfiehlt eine 7‑tägige Behandlungsdauer bei nosokomialer Pneumonie (sowohl bei einer im Krankenhaus erworbenen als auch bei einer beatmungsassoziierten Pneumonie) [210].

Bei der schwersten Form der Peritonitis, der postoperativen Peritonitis kritisch kranker Patienten auf der Intensivstation, wurde in einer multizentrischen, randomisierten, prospektiven Studie die Effektivität und Sicherheit einer 8‑tägigen Antibiotikatherapie mit einer 15-tägigen Therapie verglichen. 249 Patienten mit abgeschlossener Fokussanierung wurden an Tag 8 randomisiert, und es wurde entweder die Antibiotikatherapie beendet oder weitere 7 Tage Antibiotika verabreicht. Die Antibiotika-freien Tage (primärer Endpunkt) waren in der 8‑Tage-Gruppe signifikant höher als in der 15-Tage-Gruppe: 15 (6–20) vs. 12 (6–13) Tage, *P* < 0,0001. Die Sterblichkeit, die Dauer des Intensivaufenthaltes, das Auftreten multiresistenter Erreger und die Rate an Re-Operationen zeigten keinen signifikanten Unterschied [209].

Die Behandlungsdauer bei Blutstrominfektionen durch Enterobacterales wurde in einer Metaanalyse untersucht. Eine Therapiedauer von ≤ 10 Tagen zeigte keine signifikanten Unterschiede im Vergleich zu einer Therapiedauer von > 10 Tagen in Bezug auf 30- und 90-Tage-Sterblichkeit. Jedoch lagen vor allem Beobachtungsstudien zugrunde, mit dem entsprechenden Risiko für Verzerrungen [211].

Eine Metaanalyse von Kubo et al. [212] kommt zu dem Ergebnis, dass bei schweren Infektionen, insbesondere bei beatmungsassoziierter Pneumonie und intraabdomineller Infektion mit Sepsis, eine 7‑tägige Antibiotikagabe ausreichend sein kann. Allerdings wurde die Qualität der Evidenz als niedrig beurteilt [212].

Eine weitere Metaanalyse bei Patienten mit gramnegativer Bakteriämie zeigt für hämodynamisch stabile Patienten, die 48 h vor Beendigung der antiinfektiven Therapie fieberfrei waren, keinen Unterschied bezüglich Sterblichkeit, Hospitalisierungsdauer und Komplikationen zwischen den Gruppen mit 7 Tagen und 14 Tagen Antibiotikatherapie [213].

Eine Behandlungsdauer von < 7 Tagen war ebenso wirksam wie eine längere Behandlungsdauer in Studien zur akuten Pyelonephritis [206] und bei Haut-Weichgewebe-Infektionen [214–217].

Eine längere antimikrobielle Therapie ist erforderlich bei einem langsamen klinischen Ansprechen, nicht sanierbaren Infektionsherden, einer Bakteriämie mit *Staphylococcus aureus* einschließlich MRSA [218], einer Candidämie bzw. einer invasiven Candidiasis [153] und anderen Pilzinfektionen, einigen Virusinfektionen (z. B. durch Herpesviren) und bei Immundefizienz einschließlich Neutropenie [219].

Die mit einer verlängerten Antibiotikatherapie assoziierte Schäden (z. B. Toxizität, Resistenzentwicklung, Infektionen mit *Clostridioides difficile*) wurden in einem Umbrella-Review genauer analysiert [220]. Bei insgesamt 23.174 Patienten wurden bei 19,9 % unerwünschte Wirkungen, bei 4,8 % Superinfektionen und bei 10,6 % eine Resistenzentwicklung beobachtet. Jeder Tag einer Antibiotikatherapie war mit einem signifikanten Anstieg unerwünschter Wirkungen um 4 % und schwerer unerwünschter Wirkungen um 9 % assoziiert. Superinfektionen und antimikrobielle Resistenz waren nicht signifikant häufiger, wurden jedoch nur in wenigen Studien erfasst [220].

Daher sind Ärztinnen und Ärzte, auch bei schweren Infektionen, aufgefordert, täglich zu evaluieren, ob eine Fortführung, eine Änderung oder eine Beendigung einer antiinfektiven Therapie vorzunehmen ist. Für besondere Fragestellungen (z. B. *S.-aureus*-Bakteriämie) ist ein infektiologisches oder mikrobiologisches Konsil angeraten.

### 3.5 Deeskalation der antimikrobiellen Therapie

#### 3.10/Empfehlung/Modifiziert (Stand 2025)

- Empfehlungsgrad: Stark
- Wir empfehlen bei Patienten mit Sepsis oder septischem Schock die Messung von Serum-Procalcitonin, um die Dauer der antiinfektiven Therapie zu begrenzen, sofern eine adäquate Fokuskontrolle erreicht wurde.
- Bemerkung: Ausgenommen davon sind Patienten mit Infektionen, die eine längere Therapie erfordern (z. B. S. aureus-Bakteriämie, Endokarditis, Candidämie).
- SSC-Leitlinienadaptation/Aktualisierung der Evidenz, Änderung des Empfehlungstextes und Empfehlungsgrades
- Qualität der Evidenz:
- Therapiedauer (Tage): Moderat ⊕⊕⊕⊝
- Literatur: [221]
- Konsensstärke: 100 %

Bei Patienten mit Sepsis und septischem Schock wird die antiinfektive Therapie häufig länger durchgeführt als notwendig [222, 223]. Biomarker können neben der klinischen Bewertung zusätzliche Informationen zur Festlegung der Therapiedauer liefern. Procalcitonin wurde sowohl bei kritisch kranken Patienten als auch bei nichtkritisch kranken Patienten für den Therapiebeginn als auch für die Beendigung der Therapie am ausführlichsten untersucht [224].

In 12 RCTs wurde Procalcitonin zum Beenden der antiinfektiven Therapie bei Patienten mit Sepsis genutzt [225–236]. Eine Metaanalyse von Gutiérrez-Pizarraya et al. [237] ergab, dass die Sterblichkeit im Procalcitonin-Arm im Vergleich zur Kontrollgruppe niedriger war (RR 0,89; 95 % CI 0,79 bis 0,99). Die Dauer des Aufenthalts auf der Intensivstation war im Mittel um 1,21 Tage kürzer (95 % CI −4,16 bis 1,74), allerdings nicht signifikant. Die antiinfektive Therapie war bei den Patienten mit PCT-Steuerung um 1,98 Tage verkürzt (95 % CI −2,76 bis −1,21) [237]. Die Qualität der Evidenz wurde insgesamt als niedrig eingestuft, da es deutliche Unterschiede zwischen den Studien gab. Dies betraf u. a. die Häufigkeit der Procalcitonin-Bestimmung und die verwendeten Schwellenwerte (bzw. prozentuale Veränderung der Procalcitonin-Werte) für das Absetzen der Therapie.

Eine andere Metaanalyse mit 18 Studien und 5023 Patienten zeigte auf, dass die antiinfektive Behandlungsdauer bei Procalcitonin-gesteuerten Strategien (−1,89 Tage, 95 % CI −2,30 bis −1,47) und bei CRP-gesteuerten Strategien (−2,56 Tage, 95 % CI −4,21 bis −0,91) verkürzt war. Bei den Procalcitonin-gesteuerten Strategien war dieser Nutzen auch in Untergruppen mit kürzerer antimikrobieller Ausgangsdauer (7 bis 10 Tage) nachweisbar. Procalcitonin-gesteuerte Strategien senkten die Sterblichkeit (−27 pro 1000 Teilnehmer; 95 % CI −45 bis −7). CRP-gesteuerte Strategien führten jedoch zu keinem Unterschied in der Sterblichkeit [221]. Eine weitere Metaanalyse zeigte auf, dass eine Verkürzung der antiinfektiven Therapie durch PCT-Steuerung bei Patienten mit abdominalem Fokus nicht zuverlässig war [238].

In einem weiteren systematischen Review wurden unterschiedliche Procalcitonin-Protokolle, die in randomisierten Studien zur Beendigung der Antibiotikatherapie im Vergleich zur Standardtherapie verwendet wurden, verglichen [239]. Die Beendigung der Antibiotikatherapie, wenn Procalcitonin um mehr als 80 % des Spitzenwertes abfällt oder der Wert < 0,5 ng/ml beträgt, führte zu einer kürzeren Therapiedauer und geringeren Sterblichkeit, im Vergleich zu anderen Schwellenwerten (Abfall um mehr als 90 %, Werte < 0,1–0,25 ng/ml oder Werte < 1 ng/ml über 3 Tage).

Die Metaanalysen belegen, dass eine verkürzte antiinfektive Therapie durch Therapiesteuerung mittels Procalcitonin erreicht wird und diese mit einer geringeren Sterblichkeit einhergeht. Somit überwiegt der potenzielle Nutzen durch verminderte Toxizität [192]. Zur Kosteneffektivität liegen nur begrenzte Daten vor. In einer monozentrischen Studie wurde über geringere Krankenhauskosten im Zusammenhang mit einer PCT-gesteuerten Antibiotikatherapie bei Patienten auf der Intensivstation berichtet [240].

Zur Vermeidung von Therapieversagen ist bei *S.-aureus*-Blutstrominfektionen [218], Endokarditis [241] und Candidämie [153] eine längere Therapiedauer notwendig, die nicht durch Procalcitonin-Messungen verkürzt werden kann [153, 218, 241].

## 4. Kreislauftherapie

### 4.1 Flüssigkeitstherapie

#### 4.1/Empfehlung/Modifiziert (Stand 2025)

- Empfehlungsgrad: Stark
- Wir empfehlen bei Patienten mit Sepsis oder septischem Schock balancierte Kristalloide als Flüssigkeitstherapie der ersten Wahl.
- SSC-Leitlinienadaptation/Aktualisierung der Evidenz
- Qualität der Evidenz:
- 90-Tage-Mortalität: Hoch ⊕⊕⊕⊕
- Literatur: [242]
- Konsensstärke: 100 %

Aufgrund des Fehlens von eindeutigen Vorteilen einer Verabreichung von kolloidalen Volumenersatzlösungen im Vergleich zu kristalloiden Lösungen und in Anbetracht hoher Kosten für Albuminlösungen wird eine starke Empfehlung für die Verwendung von kristalloiden Volumenersatzlösungen, im Rahmen der initialen Flüssigkeitstherapie bei Patienten mit Sepsis und septischem Schock, ausgesprochen. Zur Fragestellung, welche kristalloide Lösung zur Flüssigkeitstherapie bei Patienten mit Sepsis oder septischem Schock besser geeignet ist, wurden in den letzten Jahren mehrere RCTs durchgeführt.

Ein RCT zum Vergleich von balancierten Lösungen (Ringer-Lösung oder Plasma-Lyte-A-Lösung) und 0,9 % NaCl an insgesamt 15.802 kritisch kranken Patienten zeigte bezüglich eines kombinierten Endpunktes (Sterblichkeit, neue Nierenersatztherapie und anhaltende Nierenfunktionsstörung) signifikante Vorteile für die balancierten Lösungen. Dies traf in besonderem Maße auf die Subgruppe der Patienten mit Sepsis oder septischem Schock (ca. 15 % aller Patienten) zu (OR 0,8; CI 0,67 bis 0,94, *P* = 0,01) [243].

In einer weiteren Studie (SPLIT-Trial) mit 2278 Intensivpatienten konnten keine positiven Effekte einer balancierten Lösung im Vergleich zu einer 0,9-%-NaCl-Lösung gefunden werden. Diese Studie ist bezüglich der Aussagefähigkeit bei Sepsis jedoch stark eingeschränkt, da lediglich 3,7 % (*n* = 84) der Patienten eine Sepsis aufwiesen [244]. In die abschließende Analyse des BaSICS-RCT wurden die Daten von 10.520 Intensivpatienten eingeschlossen. Der primäre Endpunkt, die Sterblichkeit nach 90 Tagen, unterschied sich nicht zwischen Patienten, die mit NaCl 0,9 % oder einer balancierten Vollelektrolytlösung behandelt wurden. Auch in der Subgruppe der Patienten mit Sepsis zeigte sich kein signifikanter Unterschied [245]. Das im Januar 2022 veröffentlichte PLUS-RCT verglich ebenfalls ein balanciertes Kristalloid mit einer 0,9 %igen NaCl-Lösung bei kritisch kranken Patienten, von denen ca. 43 % an einer Sepsis litten. Auch in dieser Studie zeigte sich weder im Gesamtkollektiv noch in den Subgruppen ein signifikanter Unterschied bezüglich der 90-Tage-Sterblichkeit [246].

Eine diese Studien einschließende Metaanalyse zeigte anhand der Daten von 34.450 kritisch kranken Patienten ein Relatives Risiko für die 90-Tage-Sterblichkeit von 0,96 mit einem 95 %-Konfidenzintervall von 0,91 bis 1,01 bei Verwendung balancierter Kristalloide im Vergleich zu NaCl 0,9 %. Eine Bayes’sche Analyse dieser Daten ergab eine Wahrscheinlichkeit von 89,5 %, dass der Einsatz von balancierten Kristalloiden bei kritisch kranken Patienten mit einer Reduktion der Sterblichkeit verbunden ist. Ein ähnlicher Trend zeigte sich in der Subgruppe der septischen Patienten mit einem Relativen Risiko von 0,93 (95 % CI 0,86 bis 1,01) [242].

Der Empfehlungsgrad „starke Empfehlung“ für balancierte Lösungen trägt dem Umstand Rechnung, dass im Hinblick auf den isolierten Endpunkt Sterblichkeit für Patienten mit Sepsis und septischem Schock zwar kein signifikanter positiver Effekt gezeigt werden konnte, jedoch eine hohe Wahrscheinlichkeit für eine geringere Sterblichkeit besteht. Zusätzlich zeigten sich im Hinblick auf kombinierte Studienendpunkte und die Vermeidung oder Verminderung von Organdysfunktionen (Niere) positive Effekte durch Anwendung balancierter kristalloider Lösungen.

#### 4.2/Empfehlung/Modifiziert (Stand 2025)

- Empfehlungsgrad: Schwach
- Wir schlagen vor, bei Patienten mit Sepsis oder septischem Schock Albumin additiv zu balancierten Kristalloiden zu verabreichen, wenn große Mengen an Flüssigkeit benötigt werden, um eine hämodynamische Stabilität zu erreichen.
- SSC-Leitlinienadaptation/Aktualisierung der Evidenz und Änderung des Empfehlungstextes
- Qualität der Evidenz:
- 28-Tage-Sterblichkeit: Moderat ⊕⊕⊕⊝
- Literatur: [247]
- Konsensstärke: 89 %

Zu der Fragestellung, ob kolloidale Lösungen in Kombination mit einer kristalloiden Lösung als Flüssigkeitstherapie bei Patienten mit Sepsis oder septischem Schock vorteilhaft sind, liegen nur wenige Daten aus klinischen Studien vor. Ein Cochrane-Review mit 12.492 kritisch kranken Patienten, in dem Albumin mit Kristalloiden verglichen wurde, zeigte keinen Unterschied in der 30-Tage-Sterblichkeit (RR 0,98; 95 % CI 0,92 bis 1,04), der 90-Tage-Sterblichkeit (RR 0,98; 95 % CI 0,92 bis 1,04) oder der Notwendigkeit für eine Nierenersatztherapie (RR 1,11; 95 % CI 0,96 bis 1,27) [248].

Die größte klinische Studie zur Sepsis, die ALBIOS-Studie, in der bei 1818 Patienten mit Sepsis oder septischem Schock eine Kombination von Albumin 20 % und Kristalloiden (Behandlungsziel: Serumalbuminkonzentration von 30 g/L) mit alleinigen Kristalloiden verglichen wurde, zeigte keinen Unterschied in der 28-Tage- (RR 1,0; 95 % CI 0,87 bis 1,14) oder der 90-Tage-Sterblichkeit (RR 0,94; 95 % CI 0,85 bis 1,05) [249]. Nur bei Patienten mit septischen Schock ließ sich in einer Post-hoc-Subgruppenanalyse für Albumin ein leichter Vorteil zeigen (RR 0,87; 95 % CI 0,77 bis 0,99). Eine Metaanalyse aus dem Jahr 2023 von Geng et al. [247], die die Daten für Subgruppen getrennt darstellte, ergab ebenfalls, dass Albumininfusionen das Mortalitätsrisiko bei Patienten mit septischem Schock signifikant reduzieren (OR 0,85; CI 0,74 bis 0,99). Dabei schien der Einsatz von 20 % Albumin (OR 0,81 [0,67, 0,98]; *P* = 0,03) einen größeren Effekt zu haben als eine Konzentration von 4 % oder 5 % (OR 0,89 [0,68, 1,08]; *P* = 0,15) [247].

Die Empfehlung, bei Patienten, die große Mengen an Kristalloiden für die hämodynamische Stabilisierung erhalten haben, Albumin in Betracht zu ziehen, stützt sich ebenso auf den in Studien beobachteten höheren Blutdruck und die geringere Nettoflüssigkeitsbilanz bei Albuminanwendung (kumulativ minus 900 ml in den ersten 7 Tagen) [2, 249, 250]. Aufgrund der begrenzten Datenlage kann kein Grenzwert für die kristalloide Infusion festgelegt werden, ab dem die additive Albumingabe in Betracht gezogen werden sollte. Auch ein erniedrigter Plasmaalbuminspiegel ohne anhaltenden Flüssigkeitsbedarf soll nicht als alleinige Indikation für die Albumingabe verwendet werden. Jedoch ist anzunehmen, dass Patienten mit niedrigen Plasmaalbuminspiegeln (< 30 g/l) mehr von einer Albumingabe profitieren als Patienten mit normwertigen Plasmaalbuminspiegeln. Zusätzlich wurde in der Studie von Caironi et al. [249] der Grenzwert von 30 g/l in der Interventionsgruppe angestrebt, weswegen der Plasmaalbuminspiegel bei der Therapieentscheidung berücksichtigt werden sollte [249].

Die Empfehlung wurde bei Vorlage der Konsultationsfassung durch die Deutsche Gesellschaft für Anästhesiologie und Intensivmedizin e. V. (DGAI) abgelehnt. Eine wissenschaftlich-fachliche Begründung befindet sich im Leitlinienreport (Kap. 5, Verabschiedung durch die Vorstände der herausgebenden Fachgesellschaften/Organisationen).

#### 4.3/Empfehlung/Bestätigt (Stand 2025)

- Empfehlungsgrad: Stark
- Wir empfehlen, bei Patienten mit Sepsis oder septischem Schock Hydroxyethylstärke zur Flüssigkeitstherapie NICHT anzuwenden.
- SSC-Leitlinienadoption
- Qualität der Evidenz:
- Mortalität (> 28 Tage): Hoch ⊕⊕⊕⊕
- Literatur: [251]
- Konsensstärke: 100 %

Hydroxyethylstärke-Lösungen (HES) sind Kolloide, bei denen Sicherheitsbedenken bei Patienten mit Sepsis bestehen [252]. Eine Metaanalyse von randomisierten kontrollierten Studien mit geringem Verzerrungspotenzial zeigte, dass die Nutzung von HES zu einem höheren Sterberisiko im Vergleich zu anderen Flüssigkeiten (RR 1,11; 95 % CI 1,01 bis 1,22; hohe Evidenzqualität) führte [251]. Dies entspricht 34 zusätzlichen Todesfällen pro 1000 Patienten. Des Weiteren führte die HES-Nutzung zu einem höheren Risiko für die Notwendigkeit einer Nierenersatztherapie (RR 1,36; 95 % CI 1,08 bis 1,72; hohe Evidenzqualität).

Eine anschließende Metaanalyse konzentrierte sich auf die akute Flüssigkeitstherapie bei Patienten mit Sepsis oder septischem Schock und ergab, dass HES zu einem höheren Sterberisiko (10 RCTs; OR 1,13; CrI 0,99–1,30; hohe Evidenzqualität) und einem höheren Bedarf einer Nierenersatztherapie (7 RCTs; OR 1,39; CrI 1,17–1,66; hohe Evidenzqualität) im Vergleich zu Kristalloiden führt. Beim Vergleich von Albumin mit HES führte Albumin zu einem geringeren Sterberisiko (OR 0,73; CrI 0,56–0,93; mittlere Evidenzqualität) und zeigte eine Tendenz zu einem geringeren Bedarf an Nierenersatztherapien (OR 0,74; CrI 0,53–1,04; geringe Evidenzqualität) [253]. Die unerwünschten Folgen der Verwendung von HES (erhöhtes Sterberisiko und vermehrter Bedarf für eine Nierenersatztherapie) und die mittlere bis hohe Qualität der entsprechenden Evidenz begründen die starke Empfehlung gegen die Nutzung von HES im Rahmen der Flüssigkeitstherapie von Patienten mit Sepsis oder septischem Schock.

#### 4.4/Empfehlung/Modifiziert (Stand 2025)

- Empfehlungsgrad: Schwach
- Wir schlagen vor, bei Patienten mit Sepsis und septischem Schock Gelatine NICHT anzuwenden.
- SSC-Leitlinienadoption
- Qualität der Evidenz:
- Anaphylaxie: Moderat ⊕⊕⊕⊝
- Literatur: [253–256]
- Konsensstärke: 100 %

Es ist kaum Evidenz für den Einsatz von Gelatine als synthetisches Kolloid für die Volumentherapie von Patienten mit Sepsis und septischem Schock vorhanden. Eine Metaanalyse bei Patienten mit Sepsis ergab keinen eindeutigen Effekt auf die Mortalität im Vergleich zu Kristalloiden (OR 1,24; 95 % CI 0,61 bis 2,55) [253]. Eine Metaanalyse deutete auf ein höheres, jedoch nicht signifikantes Risiko für die Notwendigkeit einer Nierenersatztherapie bei Gelatine im Vergleich zu NaCl 0,9 % (OR 1,27; 95 % CI 0,44 bis 3,64) und balancierten Kristalloiden (OR 1,50; 95 % CI 0,56 bis 3,96) hin, sodass die Frage eines tatsächlichen Risikos offen bleibt [255]. Eine weitere Metaanalyse, die die Anwendung von Gelatinelösungen bei Patienten im Rahmen einer Anästhesieeinleitung untersuchte, zeigte ein signifikant erhöhtes Risiko für anaphylaktische Reaktionen (RR 3,01; 95 % CI 1,27 bis 7,14) [256]. Einschränkend muss aber erwähnt werden, dass diese Aussage allein auf einer 30 Jahre alten Originalpublikation von Lorenz et al. [257] basiert, in der Patienten mit Haemaccel in 32 % und Patienten mit Placebo in 8 % eine anaphylaktische Reaktionen erlitten [257]. In Anbetracht der fehlenden Evidenz aus randomisierten kontrollierten Studien, nicht auszuschließenden Sicherheitsbedenken aus den bestehenden Metaanalysen und häufiger untersuchten Alternativen, wie Albumin, schlagen wir vor, von der Verwendung von Gelatine bei Patienten mit Sepsis oder septischem Schock abzusehen.

Die Empfehlung wurde bei Vorlage der Konsultationsfassung durch die Deutsche Gesellschaft für Anästhesiologie und Intensivmedizin e. V. (DGAI) abgelehnt. Eine wissenschaftlich-fachliche Begründung befindet sich im Leitlinienreport (Kap. 5, Verabschiedung durch die Vorstände der herausgebenden Fachgesellschaften/Organisationen).

### 4.2 Vasoaktive Substanzen

#### 4.5/Empfehlung/Modifiziert (Stand 2025)

- Empfehlungsgrad: Stark
- Wir empfehlen bei Patienten mit Sepsis oder septischem Schock Noradrenalin als Vasopressor erster Wahl.
- SSC-Leitlinienadoption
- Qualität der Evidenz:
- Im Vergleich mit folgenden Vasopressoren:
- Dopamin: Hoch ⊕⊕⊕⊕
- Vasopressin: Moderat ⊕⊕⊕⊝
- Epinephrin: Niedrig ⊕⊕⊝⊝
- Selepressin: Niedrig ⊕⊕⊝⊝
- Angiotensin II: Sehr niedrig ⊕⊝⊝⊝
- Literatur: [258, 259]
- Konsensstärke: 100 %

#### 4.6/Empfehlung/Modifiziert (Stand 2025)

- Empfehlungsgrad: Schwach
- Wir schlagen vor, bei Patienten mit septischem Schock Vasopressin zu ergänzen, wenn mit Noradrenalin alleine kein ausreichender Blutdruck erzielt werden kann.
- SSC-Leitlinienadaptation/Änderung des Empfehlungstextes
- Qualität der Evidenz:
- Mortalität: Moderat ⊕⊕⊕⊝
- Neuaufgetretene Organdysfunktion: Moderat ⊕⊕⊕⊝
- Literatur: [260–262]
- Konsensstärke: 100 %

Die physiologischen Effekte von Vasopressoren und einer Kombination von inotropen Medikamenten und Vasopressoren bei septischem Schock wurden in mehreren Reviews beschrieben [263–271].

Noradrenalin erhöht den MAP aufgrund seiner vasokonstriktiven Wirkungen, bei geringen Veränderungen der Herzfrequenz und einer geringeren Zunahme des Schlagvolumens im Vergleich zu Dopamin.

Ein aktuelles systematisches Review mit Metaanalyse, die 11 randomisierte Studien umfasste (*n* = 1710) und einen Vergleich von Noradrenalin mit Dopamin beinhaltete, befürwortete die Verwendung von Noradrenalin wegen verringerter Mortalität (RR 0,89; 95 % CI 0,81 bis 0,98, hohe Evidenzqualität) und eines geringeren Risikos für Arrhythmien (RR 0,48; 95 % CI 0,40 bis 0,58; hohe Evidenzqualität) [258]. Studien deuten darauf hin, dass eine Epinephrinapplikation schädliche Auswirkungen auf die Durchblutung des Splanchnicusgebietes hat und eine Hyperlaktatämie verursachen könnte.

Ein RCT, in dem Noradrenalin mit Epinephrin verglichen wurde, ergab keinen Unterschied bei der Mortalität, wies aber eine Zunahme der medikamentenbezogenen Nebenwirkungen bei der Nutzung von Epinephrin nach [259]. Auch eine Metaanalyse von vier randomisierten Studien (*n* = 540), in der Noradrenalin mit Epinephrin verglichen wurde, wies keinen signifikanten Unterschied in Bezug auf die Mortalität nach (RR 0,96; CI 0,77–1,21; niedrige Evidenzqualität) [258]. Epinephrin kann die aerobe Laktatproduktion erhöhen, indem es die adrenergen Skelettmuskel-Beta-2-Rezeptoren stimuliert, und könnte auf diese Weise die Nutzung der Laktat-Clearance beeinträchtigen, was die Steuerung der Flüssigkeitstherapie betrifft [272].

Studien zeigten, dass die Vasopressin-Plasmakonzentrationen im frühen septischen Schock erhöht sind, aber bei der Mehrheit der Patienten nach 24 bis 48 h in den normalen Wertebereich zurückgehen, während der Schockzustand andauert [273]. Dieses Ergebnis wird als relative Vasopressin-Defizienz bezeichnet, da bei Vorliegen einer Hypotonie eine erhöhte Vasopressin-Konzentration zu erwarten wäre. Niedrige Dosen Vasopressin können daher bei Patienten, welche einen refraktären Schock unter Nutzung anderer Vasopressoren aufweisen, den Blutdruck steigern.

In der VASST-Studie wurde Noradrenalin als Monosubstanz mit Noradrenalin plus Vasopressin in einer Dosierung von 0,03 U/min verglichen. Es ergab sich kein signifikanter Unterschied in Bezug auf die 28-Tage-Sterblichkeit in der Intent-to-treat-Population [262]. Eine geplante A‑priori-Subgruppenanalyse zeigte eine Verbesserung der Überlebensrate bei jenen Patienten, die bei Randomisierung mit Noradrenalin in einer Dosierung von < 15 µg/min zusätzlich Vasopressin erhielten. Die Rationale für diese Stratifizierung bei Studienplanung war jedoch auf einen potenziellen Nutzen bei denjenigen Patienten ausgelegt, die ≥ 15 µg/min Noradrenalin benötigten. Höhere Dosen von Vasopressin sind mit einem erhöhten Risiko für kardiale und Splanchnikus-Ischämien verbunden und sind daher Situationen vorbehalten, in denen alternative Vasopressoren versagt haben [260].

In der VANISH-Studie [261] wurden 409 Patienten mit septischem Schock im Rahmen eines 2 × 2-faktoriellen Designs randomisiert: der Behandlung mit Vasopressin in Kombination mit Placebo, oder Hydrocortison, oder der Behandlung mit Noradrenalin mit Placebo, oder Hydrocortison zugewiesen. Es wurde kein signifikanter Unterschied in Bezug auf die Tage ohne Nierenversagen oder Tod festgestellt. Allerdings wurde in der Vasopressin-Gruppe seltener eine Nierenersatztherapie durchgeführt [261].

In eine Metaanalyse, in die die Ergebnisse der VANISH-Studie mit einbezogen wurden, wurden Daten aus insgesamt neun Studien (*n* = 1324 Patienten mit septischem Schock) eingeschlossen, in denen Noradrenalin mit Vasopressin (oder Terlipressin) verglichen wurden. Diese demonstrierte keinen signifikanten Unterschied in Bezug auf die Mortalität (RR 0,89; 95 % CI 0,79 bis 1,00; mittlere Evidenzqualität). Die Ergebnisse fielen ähnlich aus, nachdem Studien ausgeschlossen wurden, die eine Kombination aus Noradrenalin und Vasopressin im Interventionsarm verwendet hatten (RR 0,89; 95 % CI 0,77 bis 1,02) [260, 274–278].

Es mangelt an großen Studien, in denen Vasopressin mit anderen Vasopressoren bei septischem Schock verglichen wurde. Die zu Vasopressin publizierten Daten unterstützen mehrheitlich einen Einspareffekt in Bezug auf die Noradrenalin-Dosis. Im Hinblick auf den Effekt von Vasopressin auf die Mortalität besteht allerdings Unsicherheit. Noradrenalin bleibt daher der Vasopressor der ersten Wahl bei der Behandlung von Patienten mit septischem Schock.

Wir empfehlen nicht die Nutzung von Vasopressin als Erstlinien-Vasopressor für die Unterstützung des MAP und würden zur Vorsicht raten, wenn es bei Patienten genutzt wird, die nicht euvolämisch sind oder höhere Dosen als 0,03 U/min erhalten. Aufgrund der oben beschriebenen Verbesserung der Überlebensrate, besonders bei Patienten, die einen geringeren Noradrenalin-Bedarf hatten, erachtet es die Leitliniengruppe als sinnvoll, ab einem Dosisbereich von 0,25–0,5 μg/kg/min für die Noradrenalinlaufrate Vasopressin zu ergänzen. Dieser entspricht den Empfehlungen der SSC-Leitlinie und entspricht der derzeitigen klinischen Praxis der Autoren [2]; belastbare Studiendaten dazu liegen bisher nicht vor.

Angiotensin II ist ein körpereigenes Hormon des Renin-Angiotensin-Aldosteron-Systems (RAAS) und führt vorwiegend über Stimulation von AT1-Rezeptoren direkt zu einer Vasokonstriktion sowie über Stimulation der Aldosteron- und ADH-Freisetzung zu einer Natrium- und Wasserretention. Als weiterer nicht katecholaminerger Vasopressor, neben Vasopressin und Methylenblau, stellt Angiotensin II damit einen vielversprechenden Therapieansatz des distributiven Schocks dar.

Die 2017 publizierte ATHOS-III-Studie [279] ist das bisher einzige größere RCT, die dessen Anwendung im septischen Schock an 321 Patienten untersucht hat. Im Vergleich zu Placebo konnte die Wirksamkeit im Sinne einer Steigerung des mittleren arteriellen Blutdrucks gezeigt werden. Eine statistisch signifikante Verbesserung der Sterblichkeit konnte als sekundärer Endpunkt nicht gezeigt werden [279]. Weitere Studien zur Nutzung von Angiotensin II sind notwendig, sodass auf Basis der aktuellen Studienlage die Verwendung außerhalb von Studien nicht empfohlen werden kann.

Anmerkung: Alle Patienten, die eine Vasopressor-Therapie benötigen, sollten einen arteriellen Katheter erhalten, sobald dies praktisch umsetzbar ist und die personellen Ressourcen vorhanden sind. In Schockzuständen kann die Abschätzung des Blutdrucks unter Verwendung einer konventionellen Blutdruckmanschette, insbesondere bei automatisierten Messsystemen, ungenau ausfallen. Die Nutzung eines arteriellen Zugangs ermöglicht eine genauere und besser reproduzierbare Messung des arteriellen Drucks und eine Schlag-zu-Schlag-Analyse, sodass Entscheidungen bezüglich der Therapie auf Grundlage von sofort verfügbaren und reproduzierbaren Blutdruckinformationen getroffen werden können [280, 281]. Die Nutzung von radial-arteriellen Kathetern ist im Allgemeinen sicher.

Eine systematische Überprüfung von Beobachtungsstudien ergab, dass die Inzidenz von Ischämien der Gliedmaßen und Blutungen bei weniger als 1 % liegt, wobei es sich bei der häufigsten Komplikation um lokalisierte Hämatome handelt (14 %) [282]. Die Komplikationsraten können geringer ausfallen, wenn eine ultraschallgesteuerte Punktionstechnik genutzt wird [283].

Ein systematisches Review zeigte im Vergleich zu radial-arteriellen Kathetern ein höheres Infektionsrisiko, wenn femoral-arterielle Katheter genutzt wurden (RR 1,93; 95 % CI 1,32 bis 2,84). Die gepoolte Gesamtinzidenz von Blutkreislaufinfektionen betrug 3,4 pro 1000 Kathetern [284]. Die arteriellen Katheter sollten entfernt werden, sobald keine hämodynamische Überwachung mehr benötigt wird, um das Komplikationsrisiko zu minimieren.

#### 4.7/Empfehlung/NEU, Stand:2025

- EK
- Aufgrund der aktuellen Datenlage kann bei Patienten mit septischem Schock weder für noch gegen den Einsatz von Methylenblau eine Empfehlung ausgesprochen werden.
- Zusätzliche DSG-Leitlinienempfehlung
- Konsensstärke: 100 %

Im septischen Schock ist die Aktivität der löslichen Guanylatzyklase (sGC) gesteigert, was zu einem Tonusverlust der Gefäßwandmuskulatur führt. Methylenblau bewirkt eine Hemmung der löslichen Guanylatzyklase und vermittelt dadurch seine Vasopressorwirkung [285].

Die 2023 veröffentlichte Studie von Ibarra-Estrada et al. [286] untersuchte die Gabe von Methylenblau in einer randomisierten, placebokontrollierten, doppelt verblindeten Studie bei 91 Patienten mit septischem Schock. Die Behandlung mit Methylenblau reduzierte die Zeit bis zur Beendigung der Vasopressor-Therapie und erhöhte die Anzahl der Tage ohne Vasopressoren über einen Zeitraum von 28 Tagen. Zudem verringerte sie die Aufenthaltsdauer auf der Intensivstation und im Krankenhaus, ohne dass unerwünschte Wirkungen auftraten [286]. Die Sterblichkeit lag in der Methylenblau-Gruppe bei 33 % verglichen mit 46 % in der Kontrollgruppe (RR 0,76; 95 % CI 0,55 bis 1,05; *P* = 0,23).

Eine Metaanalyse zu Methylenblau bei septischem Schock von Fernando et al. [287] zeigte eine Reduktion der Sterblichkeit, eine kürzere Dauer des Krankenhausaufenthalts und der Vasopressor-Therapie ohne Auftreten von unerwünschten Wirkungen [287].

Da die meisten der in die Metaanalyse eingeschlossenen Studien eine geringe Fallzahl aufwiesen und bisher nur ein größeres RCT zur Anwendung von Methylenblau im septischen Schock publiziert wurde, kann bis zur Überprüfung der Ergebnisse durch größere RCTs keine Empfehlung für oder gegen den Einsatz von Methylenblau bei Patienten mit septischem Schock gegeben werden.

#### 4.8/Empfehlung/NEU (Stand 2025)

- Empfehlungsgrad: Schwach
- Wir schlagen vor, bei Patienten mit septischem Schock Terlipressin NICHT anzuwenden.
- SSC-Leitlinienadoption
- Qualität der Evidenz:
- Mortalität: Niedrig ⊕⊕⊝⊝
- Literatur: [274, 277, 288–294]
- Konsensstärke: 100 %

Terlipressin ist ein synthetisches Analogon von Vasopressin, das durch enzymatische Spaltung zu einer prolongierten Freisetzung von Vasopressin und damit zu einer Verlängerung der Wirkdauer führt.

In einem RCT mit 617 Patienten wurde die Anwendung von Terlipressin als Vasopressor bei Patienten mit septischem Kreislaufversagen im Vergleich zu Noradrenalin untersucht. Es zeigte sich kein signifikanter Unterschied bezüglich des primären Endpunkts der 28-Tage-Sterblichkeit oder der Veränderung des SOFA-Scores im Verlauf. Jedoch war die Rate schwerer unerwünschter Ereignisse (zumeist digitale Ischämien) in der mit Terlipressin behandelten Gruppe erhöht [288]. Zusätzlich gibt es einen Rote-Hand-Brief zu Terlipressin, welcher bei Patienten mit hepatorenalem Syndrom Typ 1 auf ein erhöhtes Risiko für eine schwere oder tödliche Ateminsuffizienz sowie ein erhöhtes Risiko für Sepsis hinweist [295, 296].

### 4.3 Inotropika

#### 4.9/Empfehlung/Modifiziert (Stand 2025)

- Empfehlungsgrad: Schwach
- Wir schlagen vor, bei Patienten mit Sepsis oder septischem Schock, bei denen Hinweise für eine kardiale Dysfunktion mit persistierender Hypoperfusion trotz angemessenem Flüssigkeitsstatus und arteriellem Blutdruck bestehen, Dobutamin zu verwenden.
- SSC-Leitlinienadaptation/Änderung des Empfehlungstextes
- Qualität der Evidenz:
- Mortalität: Niedrig ⊕⊕⊝⊝
- Literatur: [297]
- Konsensstärke: 100 %

Eine myokardiale Dysfunktion kann bei einigen Patienten mit septischem Schock auftreten, wobei das HZV üblicherweise durch eine ventrikuläre Dilatation, Tachykardie und einen reduzierten vaskulären Widerstand aufrechterhalten wird [298]. Ein gewisser Anteil dieser Patienten kann allerdings über eine verringerte kardiale Reserve verfügen und daher nicht in der Lage sein, ein adäquates HZV zu erreichen, um ein angemessenes Sauerstoffangebot sicherzustellen. Die Erkennung einer derart reduzierten kardialen Reserve kann eine Herausforderung darstellen. Bildgebende Verfahren, die eine verringerte Ejektionsfraktion zeigen, müssen nicht notwendigerweise auch ein unzureichendes HZV bedeuten. Andererseits ist besonders im distributiven Schock mit geringer Nachlast die Ejektionsfraktion kein verlässlicher Marker, um eine eingeschränkte kardiale Funktion zu detektieren. Eine frühe und wiederholte echokardiographische Beurteilung von Patienten mit septischem Schock erscheint daher sinnvoll. Begleitende Messungen des HZV in Kombination mit der parallelen Beurteilung von Perfusionsparametern sind zu bevorzugen [299, 300].

Eine routinemäßige Erhöhung des Herzzeitvolumens auf im Vorfeld festgelegte „supranormale“ Werte führt eindeutig nicht zu einer Verbesserung der Behandlungsergebnisse, wie in zwei großen prospektiven klinischen Studien bei kritisch kranken Patienten mit Sepsis unter Behandlung mit Dobutamin gezeigt wurde [280, 301, 302]. Einige Patienten könnten jedoch von einer verbesserten Gewebeperfusion infolge einer inotropen Therapie, die auf eine Erhöhung des Sauerstoffangebots ausgerichtet ist, profitieren. In dieser Situation stellt Dobutamin für Patienten mit einem bestätigten oder vermuteten niedrigen HZV, bei denen ein angemessener linksventrikulärer Füllungsdruck (oder die klinische Beurteilung einer angemessenen Flüssigkeitstherapie) sowie ein angemessener MAP vorliegt, die inotrope Therapie der ersten Wahl dar. Die beste Möglichkeit, diese Art von Therapie zu steuern, besteht darin, die Reaktion von Perfusionsindizes auf den (gemessenen) Anstieg des HZV zu monitorisieren [280].

Die Daten, welche die Nutzung von Dobutamin unterstützen, sind hauptsächlich physiologischer Art und zeigen eine verbesserte Hämodynamik und einige Verbesserungen bei den Perfusionsindizes, welche eine klinische Verbesserung, abnehmende Laktatspiegel und eine ScvO_2_-Verbesserung umfassen können.

RCTs, die die Effekte von Dobutamin gegenüber einem Placebo im Hinblick auf die klinischen Behandlungsergebnisse verglichen haben, sind allerdings nicht bekannt. Die Mortalität von Patienten, die randomisiert einer Therapie mit Dobutamin zusätzlich zu Noradrenalin zugewiesen worden waren, wies keinen Unterschied im Vergleich zu einer Therapie mit Epinephrin auf, wobei diese Studie aufgrund einer unzureichenden Patientenzahl nicht über genügend Aussagekraft verfügt [280].

Dobutamin wurde ferner als inotrope Erstlinien-Therapie im Rahmen der Standardbehandlung in klinischen Early-Goal-Directed-Therapy-Studien verwendet, und negative Auswirkungen auf die Mortalität wurden bei dessen Verwendung nicht festgestellt [49–51, 77]. In einer 2019 publizierten Metaanalyse von insgesamt 43 RCTs zeigte sich die Kombination von Noradrenalin und Dobutamin mit einer reduzierten Sterblichkeit assoziiert [303].

Abweichend von den aktuellen SSC-Empfehlungen wird die alternative Anwendung von Epinephrin nicht empfohlen. Dies begründet sich darin, dass in Zusammenhang mit der Verwendung von Epinephrin eine erhöhte Sterblichkeit im kardiogenen Schock, ein vermehrtes Auftreten von Arrhythmien sowie eine gesteigerte Laktatbildung beschrieben worden ist und damit die Nutzbarkeit von Laktat zur Steuerung der hämodynamischen Therapie beeinträchtigt werden könnte [304–306].

#### 4.10/Empfehlung/Modifiziert (Stand 2025)

- Empfehlungsgrad: Schwach
- Wir schlagen vor, bei Patienten mit septischem Schock Levosimendan NICHT einzusetzen.
- SSC-Leitlinienadaptation/Änderung des Empfehlungstextes
- Qualität der Evidenz:
- Mortalität: Niedrig ⊕⊕⊝⊝
- Literatur: [307, 308]
- Konsensstärke: 95 %

Metaanalysen, die Levosimendan mit Dobutamin bei Patienten mit Sepsis und septischem Schock verglichen, zeigten keinen signifikanten Effekt auf die Mortalität [307, 309].

In einem RCT, in dem 516 Patienten mit septischem Schock randomisiert entweder der Behandlung mit Levosimendan oder einem Placebo zugeordnet wurden, zeigte sich kein Unterschied in Bezug auf die Mortalität. Levosimendan führte jedoch zu einem signifikant erhöhten Risiko einer supraventrikulären Tachyarrhythmie im Vergleich zum Placebo (absoluter Unterschied 2,7 %; 95 % CI 0,1 %–5,3 %). Zusätzlich war die Wahrscheinlichkeit, innerhalb von 28 Tagen erfolgreich von der Beatmung entwöhnt zu werden, in der Levosimendan-Gruppe geringer als in der Placebo-Gruppe (Hazard Ratio 0,77; 95 % CI 0,60 bis 0,97; *P* = 0,03) [308]. Die Ergebnisse dieser Studie stellen die routinemäßige Verwendung von Levosimendan bei Patienten mit septischem Schock in Frage. Zu beachten ist, dass myokardiale Funktionsparameter in dieser Studie nicht erhoben wurden und somit auch nicht die Frage beantwortet wurde, inwiefern Patienten mit einer Sepsis-bedingten Erniedrigung des HZV von der inotropen Stimulation profitieren könnten. Es bleibt somit unklar, ob Levosimendan bei Patienten mit septischer Kardiomyopathie vorteilhaft ist.

Die aktuell publizierten Metaanalysen zu dieser Fragestellung liefern aufgrund ihrer unzureichenden Qualität keine neuen Erkenntnisse, sodass bei fehlendem Nutzennachweis in großen RCTs, den in der LeoPARDS-Studie [308] beobachteten negativen Effekten und den höheren Kosten die Empfehlung gegen den Einsatz bei septischen Patienten bestehen bleibt.

### 4.4 Venoarterielle ECMO

#### 4.11/Empfehlung/NEU (Stand 2025)

- EK
- Der Einsatz einer venoarteriellen ECMO bei Patienten mit einem therapierefraktären septischen Schock ohne einen zusätzlichen kardiogenen Schock kann NICHT empfohlen werden.
- Aufgrund der aktuellen Datenlage kann weder für noch gegen den Einsatz einer venoarteriellen ECMO bei Patienten mit einem therapierefraktären septischen und einem zusätzlichen kardiogenen Schock eine Empfehlung ausgesprochen werden.
- Zusätzliche DSG-Leitlinienempfehlung
- Konsensstärke: 100 %

Patienten, die an einer septischen Kardiomyopathie leiden und dadurch ein kombiniertes Schocksyndrom mit distributiver und kardiogener Komponente entwickeln, haben häufig eine sehr ausgeprägte Gewebeminderperfusion und weisen eine hohe Sterblichkeit auf [310]. Der Einsatz einer extrakorporalen Kreislaufunterstützung in Form einer venoarteriellen ECMO könnte bei diesen Patienten theoretisch eine sinnvolle Therapieoption darstellen. Jedoch liegen dazu bisher keine randomisierten kontrollierten Studienergebnisse vor. In einer retrospektiven, Propensity-Score-gewichteten Analyse von 82 Patienten mit kardialer Dysfunktion und septischem Schock führte die Anwendung einer venoarteriellen ECMO zu einer signifikanten Verbesserung des Überlebens [311].

Zusätzlich untersuchten mehrere randomisierte kontrollierte Studien die Anwendung einer venoarteriellen ECMO zur hämodynamischen Stabilisierung bei Patienten mit kardiogenem Schock (zumeist im Rahmen eines Myokardinfarktes). Keine dieser Studien vermochte einen Überlebensvorteil für die venoarteriellen ECMO bei kardiogenem Schock zu zeigen [312, 313].

Eine aktuelle Metaanalyse von Beobachtungsstudien zeigte, dass bei Behandlung mit venoarterieller ECMO die meisten Patienten mit septischem Schock oder Sepsis-induzierter kardialer Dysfunktion überleben. Dagegen wurde bei Erwachsenen mit septischem Schock ohne schwere linksventrikuläre Funktionsstörung unter Behandlung mit venoarterieller ECMO eine hohe Sterblichkeit beobachtet [314].

Zusammenfassend ist die aktuelle Datenlage unzureichend, um eine Empfehlung zum Einsatz der venoarteriellen ECMO zur hämodynamischen Stabilisierung von Patienten mit therapierefraktärem septischem Schock und kardialer Dysfunktion abzugeben, sodass der Einsatz, auch unter Berücksichtigung der vorhandenen Erfahrung und Ressourcen, individuell abgewogen werden sollte.

#### 4.12/Empfehlung/Modifiziert (Stand 2025)

- EK
- Wir empfehlen, bei Patienten mit septischem Schock β‑Blocker zur Herzfrequenzsenkung NICHT routinemäßig anzuwenden.
- Zusätzliche DSG-Leitlinienempfehlung
- Konsensstärke: 100 %

Sepsis und septischer Schock sind unter anderem durch eine exzessive adrenerge Stimulation charakterisiert, die zu Tachykardie, Rhythmusstörungen sowie zu systolischen und diastolischen Funktionsstörungen des Myokards führen kann und mit einer erhöhten Mortalität assoziiert ist [315, 316].

Eine Applikation von β‑Blockern könnte theoretisch der adrenergen Stimulation entgegenwirken und die Herzfunktion optimieren (Senkung des myokardialen Sauerstoffbedarfs, Reduktion der Tachykardie, verbesserte diastolische Füllung/Relaxation, Rhythmusstabilisierung). Darüber hinaus gibt es Hinweise, dass β‑Blocker als Modulatoren des Immunsystems eine wichtige Rolle spielen könnten [317].

β‑Blocker werden bei Patienten mit Herzinsuffizienz und koronarer Herzkrankheit zur Verbesserung der myokardialen Funktion und zur Senkung des Reinfarktrisikos erfolgreich eingesetzt. Darüber hinaus können β‑Blocker über eine Senkung der Herzfrequenz den myokardialen Sauerstoffbedarf senken. Aus diesen Überlegungen heraus könnte der Einsatz von β‑1-selektiven Betablockern bei tachykarden Rhythmusstörungen auch in der Sepsis von Vorteil sein. Deshalb wurden insbesondere kurzwirksame β‑1-selektive Betablocker bisher für die Anwendung bei tachykarden Patienten mit septischem Schock in randomisierten kontrollierten Studien untersucht [318, 319].

Morelli et al. [318] konnten in einem RCT bei 77 Patienten mit septischem Schock durch eine kontinuierliche Esmololinfusion eine Reduktion der Herzfrequenz ohne negative Effekte auf die Hämodynamik zeigen. Als sekundärer Endpunkt ergab sich eine reduzierte Sterblichkeit in der mit Esmolol therapierten Patientengruppe [318]. Diese Studie ist jedoch mit erheblichen Einschränkungen assoziiert, sodass sie lediglich ein „Proof-of-Principle“ einer sicheren Herzfrequenzreduktion durch Esmolol darstellt.

Das 2023 publizierte STRESS-L-RCT bei 126 Patienten untersuchte die Anwendung von Landiolol bei Patienten mit septischem Schock und Tachykardie. Nur bei ca. 12 % der eingeschlossenen Patienten lag bei Randomisierung ein Vorhofflimmern vor, sodass davon auszugehen ist, dass die Mehrheit der Patienten eine Sinustachykardie zeigte. Die Studie wurde wegen einer möglichen Schädigung der Patienten durch die Intervention ohne signifikanten Benefit vorzeitig gestoppt [319]. Die mit Landiolol behandelte Gruppe zeigte im Durchschnitt einen höheren Noradrenalinbedarf, einen niedrigeren Blutdruck und ein höheres Laktat.

Rehberg et al. [320] untersuchten in einer randomisierten kontrollierten Studie an insgesamt 196 Patienten mit septischem Schock und persistierender Tachykardie den Einfluss einer Landiolol-Therapie auf das Erreichen einer Zielherzfrequenz von 80–94/min und deren Aufrechterhaltung ohne Erhöhung des Vasopressorbedarfs in den ersten 24 h nach Behandlungsbeginn. Sekundäre Endpunkte waren unter anderen die Sterblichkeit auf Intensivstation sowie nach 28 Tagen. Circa ein Viertel der randomisierten Patienten zeigte ein Vorhofflimmern als mögliche Ursache der Tachykardie. Ein signifikant größerer Anteil der Patienten erreichte den kombinierten primären Endpunkt aus Herzfrequenzkontrolle ohne Anstieg des Vasopressorbedarfs. Jedoch führte die Gabe des kurzwirksamen Betablockers nicht zu positiven Effekten auf die Mortalität oder sonstige sekundäre Endpunkte [320]. Eine Betablocker-Therapie bei Patienten mit septischem Schock scheint sowohl negative als auch positive hämodynamische Effekte haben zu können.

Aufgrund der negativen Ergebnisse im Hinblick auf relevante klinische Endpunkte, insbesondere der STRESS-L-Studie, bleibt die Empfehlung gegen einen routinemäßigen Einsatz von β‑Blockern zur Herzfrequenzkontrolle im Rahmen der hämodynamischen Stabilisierung septischer Patienten bestehen. Die Anwendung kurzwirksamer Betablocker zur Therapie von Tachyarrhythmien z. B. im Rahmen von Vorhofflimmern bei septischen Patienten sollte davon gesondert betrachtet werden. Zur Therapie von supraventrikulären Arrhythmien ist die Gabe kurzwirksamer Betablocker weiterhin eine Option.

Vor Einleitung einer Therapie mit β‑1-selektiven Betablockern bei tachykarden Herzrhythmusstörungen sollte jedoch ein adäquater Volumenstatus hergestellt und eine Blutdruckstabilisierung durch Vasopressoren erfolgt sein. Die Anwendung sollte unter entsprechendem hämodynamischem Monitoring titriert erfolgen und auf eine Verbesserung der hämodynamischen Situation und Gewebeperfusion abzielen [321, 322].

## 5. Beatmungstherapie

Das folgende Kapitel ist eine Adoption der aktuellen S3-Leitlinie „Invasive Beatmung und Einsatz extrakorporaler Verfahren bei akuter respiratorischer Insuffizienz“ [4] (AWMF-Reg.-Nr.: 001–021) sowie der S3-Leitlinie „Lagerungstherapie und Mobilisation von kritisch Erkrankten auf Intensivstationen“ [6] (AWMF-Reg.-Nr.: 001–015, Stand 25.07.2023).

Die in der S3-Leitlinie abgegebenen Empfehlungen zu Patienten mit ARDS umfassen in der Mehrheit Patienten mit Sepsis, septischem Schock und/oder einer Pneumonie und erfüllen somit die Kriterien der Sepsis-3-Definition. Allerdings ist in diesem Kontext keine Studie bekannt, die sich spezifisch mit septischen Patienten befasst hat.

Im folgenden Abschnitt werden die wesentlichen Empfehlungen der Leitlinienkommission zu den genannten Leitlinien zusammengefasst. Für eine detaillierte Bewertung der zugrunde liegenden Evidenz wird auf die Langfassungen der S3-Leitlinien verwiesen (https://register.awmf.org/de/leitlinien/detail/001-021).

### 5.1 Beatmungseinstellungen

#### 5.1/Empfehlung/Modifiziert (Stand 2025)

- Empfehlungsgrad: Stark
- Wir empfehlen, Patienten mit Sepsis oder septischem Schock und ARDS mit einem Tidalvolumen um 6 ml/kg IBW (4–8 ml/kg IBW) zu beatmen.
- Adoption der S3-Leitlinie Beatmung*
- Qualität der Evidenz:
- Mortalität: Moderat ⊕⊕⊕⊝
- Literatur: [323, 324]
- Konsensstärke: 100 %
*Die Leitlinie war zum Zeitpunkt der Veröffentlichung noch nicht publiziert.

Aktuelle Arbeiten untersuchten Tidalvolumen von 4–8 ml/kg IBW, dies spiegelt sich ebenfalls in anderen Leitlinien wider. Sowohl in der ESICM als auch der „ARDS Clinical Practice Guideline“ [325] wird eine Beatmung mit Tidalvolumen von 4–8 ml/kg IBW empfohlen, daher haben sich die Autoren entschlossen, bei gleicher zugrunde liegender Evidenz sich diesen Empfehlungen anzuschließen.

#### 5.2/Empfehlung/Bestätigt (Stand 2025)

- Empfehlungsgrad: Stark
- Wir empfehlen, Patienten mit Sepsis oder septischem Schock ohne ARDS mit einem mit VT von 6–8 ml/kg IBW zu beatmen.
- Adoption der S3-Leitlinie Beatmung*
- Qualität der Evidenz:
- Mortalität: Moderat ⊕⊕⊕⊝
- Literatur: [323, 324]
- Konsensstärke: 100 %
*Die Leitlinie war zum Zeitpunkt der Veröffentlichung noch nicht publiziert.

Für Patienten ohne ARDS wird die Empfehlung aus 2018 unverändert beibehalten. Einzig eine Arbeit von Simonis et al. [326] hat nachfolgend zur alten Leitlinienempfehlung explizit Patienten ohne ARDS mit invasiver Beatmung für mehr als 24 h untersucht. Hier konnte kein Unterschied in beatmungsfreien Tagen an Tag 21 (*P* = 0,71), Intensiv‑/Krankenhausverweildauer oder 28- und 90-Tage-Mortalität gezeigt werden [326].

#### 5.3/Empfehlung/Bestätigt (Stand 2025)

- Empfehlungsgrad: Schwach
- Wir schlagen vor, bei der invasiven Beatmung von Patienten mit Sepsis oder septischem Schock und akuter respiratorischer Insuffizienz den Plateaudruck (Pplat) ≤ 30 cmH_2_O zu halten.
- Adoption der S3-Leitlinie Beatmung*
- Qualität der Evidenz:
- Mortalität: Moderat ⊕⊕⊕⊝
- Literatur: [327–329]
- Konsensstärke: 100 %

#### 5.4/Empfehlung/Modifiziert (Stand 2025)

- Empfehlungsgrad: Schwach
- Wir schlagen vor, bei der invasiven Beatmung von Patienten mit Sepsis oder septischem Schock und ARDS eine inspiratorische Druckdifferenz von ≤ 14 cmH_2_O anzustreben.
- Adoption der S3-Leitlinie Beatmung*
- Qualität der Evidenz:
- Mortalität: Niedrig ⊕⊕⊝⊝
- Literatur: [327–329]
- Konsensstärke: 100 %
*Die Leitlinie war zum Zeitpunkt der Veröffentlichung noch nicht publiziert.

Die überwiegende untersuchte Literatur beschreibt einen „driving pressure“ von ≤ 14 cmH_2_O bzw. < 15 cmH_2_O, dementsprechend wurde die Formulierung angepasst.

Amato et al. [329] konnte in einer Metaanalyse von 9 RCTs mit 3562 Patienten zeigen, dass die Einhaltung des „driving pressure“ von ≤ 14 mit signifikant mit mehr Überleben assoziiert war (1-SD Anstieg ∆P: RR 1,41; 95 % CI 1,31 bis 1,51; *P* < 0,001) [329].

### 5.2 PEEP-Einstellung

#### 5.5/Empfehlung/Modifiziert (Stand 2025)

- Empfehlungsgrad: Schwach
- Wir schlagen vor, bei Patienten mit Sepsis oder septischem Schock und mildem ARDS eine orientierende Einstellung des PEEP mit Hilfe der FiO_2_/Niedrig-PEEP-Tabelle des ARDS-Netzwerks zu erwägen (Tab. 4).
- Adoption der S3-Leitlinie Beatmung*
- Qualität der Evidenz:
- Mortalität: Niedrig ⊕⊕⊝⊝
- Literatur: [330]
- Konsensstärke: 100 %

**Tab. 4:** ARDS-Netzwerk-Tabelle „lower PEEP/higher FiO_2_“

*FiO*_*2*_	0,3	0,4	0,4	0,5	0,5	0,6	0,7	0,7	0,7	0,8	0,9	0,9	0,9	1
*PEEP*	5	5	8	8	10	10	10	12	14	14	14	16	18	18–24

Quelle: DGAI et al. [4]

Die Niedrig-PEEP-FiO_2_-Tabelle führt bei Patienten mit mildem ARDS (*P*/F-Ratio 201–300) typischerweise zu PEEP-Werten zwischen 5 und 8 cmH_2_O, sofern ein PaO_2_ zwischen 60 und 90 mm Hg angestrebt wird. Im Vergleich zu einem höheren PEEP gab es nichtsignifikante Trends zu geringerer Mortalität (Relatives Risiko mit höherem PEEP 1,37; 95 % CI 0,98 bis 1,92). Der Nutzen einer Beatmung mit niedrigerem PEEP scheint also in dieser Patientengruppe gegenüber dem möglichen Schaden zu überwiegen.

#### 5.6/Empfehlung/Bestätigt (Stand 2025)

- Empfehlungsgrad: Stark
- Wir empfehlen bei Patienten mit Sepsis oder septischem Schock und moderatem oder schwerem ARDS die Anwendung eines höheren PEEP.
- Adoption der S3-Leitlinie Beatmung*
- Qualität der Evidenz:
- Mortalität: Hoch ⊕⊕⊕⊕
- Literatur: [331–333]
- Konsensstärke: 100 %
*Die Leitlinie war zum Zeitpunkt der Veröffentlichung noch nicht publiziert.

Hoher PEEP kann dazu beitragen, Atemwege offenzuhalten, Atelektasen wiederzueröffnen und dadurch Oxygenierung und Compliance zu verbessern, was potenziell eine bessere Lungenprotektion ermöglicht. Dem stehen Nebenwirkungen wie Kreislaufdepression, insbesondere bei Hypovolämie, und pulmonale Überdehnung gegenüber. Während die Anwendung eines höheren PEEP für Patienten mit mildem ARDS vermutlich nicht vorteilhaft ist (siehe vorherige Empfehlung), zeigt die Metaanalyse von Dianti et al. [331] für Patienten mit moderatem bis schwerem ARDS (*P*/F < 201 mm Hg) mit hoher Wahrscheinlichkeit, dass die Anwendung eines höheren PEEP ohne Rekrutierungsmanöver mit einer höheren Überlebenswahrscheinlichkeit einhergeht (Relatives Risiko für Mortalität mit höherem PEEP: 0,77; 95 % CI 0,60 bis 0,96). In der klinischen Praxis wird diese Erkenntnis aber oft nicht umgesetzt, was zu vermeidbarer Übersterblichkeit durch zu niedrig eingestellten PEEP führt [334]. Daher sprechen wir eine starke Empfehlung für Anwendung eines höheren PEEP bei moderatem bis schwerem ARDS aus.

#### 5.7/Empfehlung/Modifiziert (Stand 2025)

- Empfehlungsgrad: Schwach
- Wir schlagen vor, bei Patienten mit Sepsis oder septischem Schock und moderatem oder schwerem ARDS die Einstellung des PEEP mit Hilfe einer der in Tab. 5 vorgeschlagenen bettseitigen Methoden zu individualisieren.
- Adoption der S3-Leitlinie Beatmung
- Qualität der Evidenz:
- Mortalität: Sehr niedrig ⊕⊝⊝⊝
- Literatur: [330, 331, 335–348]
- Konsensstärke: 100 %
* Die Leitlinie war zum Zeitpunkt der Veröffentlichung noch nicht publiziert.

**Tab. 5:** Individualisierte Einstellung des PEEP mit bettseitigen Methoden

Verfahren zur PEEP-Einstellung	Zielparameter	Empfohlenes Vorgehen zur PEEP-Einstellung	Literatur
PEEP/FiO_2_-Tabelle	Oxygenierung (PaO_2_/FiO_2_-Verhältnis/ersatzweise SpO_2_/FiO_2_)	Ersteinstellung nach Niedrig-PEEP/FiO_2_-Tabelle, bei PaO_2_/FiO_2_-Verhältnis < 200 Wechsel auf Hoch-PEEP/FiO_2_-Tabelle. Bei klinischer Verbesserung (z. B. PaO_2_/FiO_2_-Anstieg > 25 mm Hg) Hoch-PEEP-Strategie beibehalten, andernfalls PEEP-Absenkung erwägen	Goligher et al. 2014 [335] Dianti et al. 2022 [331] Briel et al. 2010 [330]
Quasistatische Druck-Volumen-Kurve	Globale Compliance	Bestimmung der volumenabhängigen Compliance mittels quasistatischer Druck-Volumen-Kurve („Low-Flow-Kurve“); Beatmung im Bereich der besten Compliance (PEEP 2 mbar oberhalb des „unteren Inflektionspunktes“ des inspiratorischen Teils der Low-Flow-Kurve einstellen)	Amato et al. 1998 [336] Villar et al. 2006 [337] Ranieri et al. 1999 [345]
Dekrementelles PEEP-Trial	Globale Compliance	Schrittweise Absenkung des PEEP (dekrementelles PEEP-Trial) beginnend bei 24 mbar (soweit hämodynamisch toleriert) bis ca. 5 mbar unter Messung der globalen Compliance des respiratorischen Systems (CRS). Absenkungsschritte von 2–3 mbar, kleines VT und DeltaP, PEEP-Einstellung im Bereich der besten Compliance (niedrigster PEEP, der einen Abfall der CRS verhindert)	Kacmarek et al. 2016 [338] Gernoth et al. 2009 [339]
Dekrementelles PEEP-Trial	Regionale Complianceänderungen, ermittelt mit elektrischer Impedanz-tomographie (EIT): Überdehnung (regionaler Complianceverlust bei höherem PEEP) vs. Alveolarkollaps (regionaler Complianceverlust bei niedrigerem PEEP)	Dekrementelles PEEP-Trial unter druckkontrollierter Beatmung mit konstant niedrigem DeltaP (z. B. 10–12 mbar) unter Monitoring mit EIT, PEEP-Absenkungsschritte von 2–3 mbar; Auswertung von regionaler Überdehnung und Alveolarkollaps anhand regionaler Complianceänderungen. PEEP-Einstellung so, dass sowohl Überdehnung als auch Alveolarkollaps minimiert, z. B. auf den Kreuzungspunkt von Überdehnung und Kollaps	Zhao et al. 2019 [346] Hsu et al. 2021^347^ He et al. 2021 [340]
Inkrementelles PEEP-Trial	Globale Compliance	PEEP ausgehend von 5 mbar in Schritten von 2 mbar erhöhen, solange die Compliance zunimmt. PEEP-Einstellung auf den Wert mit der höchsten globalen Compliance	Pintado et al. 2013 [341]
Ösophagusdruckmessung	Endexspiratorischer transpulmonaler Druck	Bei negativen endexspiratorischen transpulmonalen Drücken (PTPe) PEEP-Erhöhung, bei positiven PTPe PEEP-Senkung, Ziel: PTPe = 0 (± 2)	Talmor et al. 2008 [342] Beitler et al. 2019 [343] Sarge et al. 2021 [344]
Lungen-Ultraschall	Reaeration Score	Summations-Score aus 12 Regionen der Lungen (6 pro Thoraxhälfte). Score: normaler Ultraschall-Muster = 0, gut abtrennbarer B‑Linien = 1, zusammenfließende B‑Linien = 2, Konsolidierungsmuster = 3	Salem et al. 2020 [348]

Quelle: DGAI et al. [4]*

*Die Leitlinie war zum Zeitpunkt der Veröffentlichung noch nicht publiziert.

Eine Individualisierung der PEEP-Einstellungen wird sowohl von der französischen [349] als auch von der britischen Leitlinie [350] empfohlen, die Leitlinie der Europäischen Gesellschaft für Intensivmedizin (ESICM) [323] äußert sich nicht zur Individualisierung des PEEP. Die aktualisierten Leitlinien der American Thoracic Society [351] geben zwar keine Empfehlung zur Individualisierung ab, betonen aber, dass die Einstellung von einer kontinuierlichen Überwachung der Atemmechanik und Hämodynamik und der Beurteilung der physiologischen Reaktion des Patienten auf PEEP begleitet und ggf. angepasst werden sollte. Ziel einer PEEP-Individualisierung ist es, eine Verbesserung des pulmonalen Gasaustausches und Vermeidung von Derekrutierung und Atelektrauma zu erreichen ohne ein Trauma durch Überdehnung von Lungenarealen und hämodynamische Kompromittierung. Es gibt zwar kein Verfahren zur PEEP-Einstellung, welches eine klare Überlegenheit hinsichtlich relevanter Outcomes gezeigt hat, allerdings konnten für die in der Tabelle vorgeschlagenen Methoden positive Effekte aufgezeigt werden, weshalb deren Anwendung mit einem schwachen Empfehlungsgrad vorgeschlagen wird.

### 5.3 Inspiratorische Sauerstoffkonzentration, FiO_2_

#### 5.8/Empfehlung/NEU (Stand 2025)

- Empfehlungsgrad: Schwach
- Wir schlagen vor, bei invasiv beatmeten Patienten mit Sepsis oder septischem Schock eine SaO_2_ bzw. SpO_2_ von 92–96 % bzw. eine paO_2_ zwischen 70–90 mm Hg (9,3–12 kPa) anzustreben.
- Adoption der S3-Leitlinie Beatmung*
- Qualität der Evidenz:
- Mortalität: Sehr niedrig ⊕⊝⊝⊝
- Literatur: [352, 353]
- Konsensstärke: 100 %
*Die Leitlinie war zum Zeitpunkt der Veröffentlichung noch nicht publiziert.

Wir übernehmen die Empfehlung der S3-Leitlinie „Invasive Beatmung und Einsatz extrakorporaler Verfahren bei akuter respiratorischer Insuffizienz“ [4] mit geändertem Wortlaut und abgeschwächtem Empfehlungsgrad: Hypoxie und Hyperoxie wurden durch Hypoxämie und Hyperoxämie ersetzt. Eine Hypoxämie ist aus rein physiologischen Gründen mit konsekutiver Sauerstoffminderversorgung der Körpergewebe mit Ischämie zu vermeiden. Hyperoxämie kann u. a. zur Bildung freier Sauerstoffradikale mit nachfolgender Zellschädigung beitragen.

Ein 2023 aktualisiertes Cochrane-Review [352] konnte keinen Unterschied bei der Mortalität zwischen einer hohen und niedrigen Oxygenierungsstrategie feststellen. Ebenso gibt es keinen Unterschied hinsichtlich schwerer unerwünschter Ereignisse oder der Lebensqualität. Lediglich bei der kumulierten Anzahl der schweren unerwünschten Ereignisse zeigt sich ein Vorteil für die restriktive Oxygenierung, allerdings mit einem klinisch sehr geringen Effekt (RR 1,04; 95 % CI 1,02 bis 1,07). Es gibt hierbei ein niedrige bzw. sehr niedrige Sicherheit, dass der wahre Effekt mit dem errechneten Effekt übereinstimmt [352].

### 5.4 Rekrutierungsmanöver

#### 5.9/Empfehlung/Modifiziert (Stand 2025)

- Empfehlungsgrad: Stark
- Wir empfehlen bei Patienten mit Sepsis oder septischem Schock KEINE Durchführung von prolongierten (> 60 s) Rekrutierungsmanövern.
- Adoption der S3-Leitlinie Beatmung*
- Qualität der Evidenz:
- Mortalität: Moderat ⊕⊕⊕⊝
- Literatur: [331, 354–361]
- Konsensstärke: 100 %
*Die Leitlinie war zum Zeitpunkt der Veröffentlichung noch nicht publiziert.

In der aktuellen S3-Leitlinie „Invasive Beatmung und Einsatz extrakorporaler Verfahren bei akuter respiratorischer Insuffizienz“ [4] wird eine starke Empfehlung gegen die Durchführung eines prolongierten Rekrutierungsmanövers ausgesprochen. Diese basiert auf mehreren Metaanalysen. Die Rekrutierung vermag zwar kurzfristig die Oxygenierung zu verbessern, jedoch ohne Reduktion von Mortalität, Intensiv- oder Krankenhausverweildauer. Durch Rekrutierungsmanöver besteht zudem die Gefahr einer akuten hämodynamischen Beeinträchtigung [331].

#### 5.10/Empfehlung/Modifiziert (Stand 2025)

- Empfehlungsgrad: Schwach
- Wir schlagen vor, bei Patienten mit Sepsis oder septischem Schock KEINE routinemäßige Durchführung von kurzen (< 60 s) Rekrutierungsmanövern durchzuführen.
- Adoption der S3-Leitlinie Beatmung*
- Qualität der Evidenz:
- Mortalität: Niedrig ⊕⊕⊝⊝
- Literatur: [331, 354–361]
- Konsensstärke: 100 %
*Die Leitlinie war zum Zeitpunkt der Veröffentlichung noch nicht publiziert.

Wir schließen uns der Empfehlung der S3-Leitlinie „Invasive Beatmung und Einsatz extrakorporaler Verfahren bei akuter respiratorischer Insuffizienz“ [4] an und empfehlen keine regelmäßige Durchführung von kurzen Rekrutierungsmanövern aus oben genannten Gründen. Dennoch kann in Einzelfällen unter individueller Nutzen-Risiko-Abwägung ein solches Manöver durchgeführt werden. Beispielhaft ist hier ein akuter Druckverlust durch akzidentielle Diskonnektion von Tubus und Respirator bei entsprechend hohem positivem endexspiratorischem Druck zu nennen, bei der der Patient eine akute und lebensbedrohliche Hypoxämie entwickelt. In diesen Fällen könnte durch ein kurzes Rekrutierungsmanöver (unter 60 s Dauer) der Gasaustausch zumindest kurzfristig verbessert werden.

### 5.5 Spontanatmung

#### 5.11/Empfehlung/Modifiziert (Stand 2025)

- Empfehlungsgrad: Schwach
- Wir schlagen vor, bei Patienten mit Sepsis oder septischem Schock frühzeitig (innerhalb der ersten 48 h nach Intubation) eine unterstützende Beatmung zur Ermöglichung von Spontanatmung unabhängig von Ursache und Art der zugrunde liegenden respiratorischen Insuffizienz einzusetzen.
- Adoption der S3-Leitlinie Beatmung*
- Qualität der Evidenz:
- Mortalität: Sehr niedrig ⊕⊝⊝⊝
- Literatur: [6, 362–367]
- Konsensstärke: 100 %
*Die Leitlinie war zum Zeitpunkt der Veröffentlichung noch nicht publiziert.

Wir schließen uns der Empfehlung der S3-Leitlinie „Invasive Beatmung und Einsatz extrakorporaler Verfahren bei akuter respiratorischer Insuffizienz“ [4] an und schlagen eine unterstützende Beatmung zur Ermöglichung von Spontanatmung vor. Bisher wurde das schwere ARDS von dieser Empfehlung ausgenommen. Neuere Studien, wie das ROSE-Trial [363], relativierten die strikte Vermeidung von Spontanatmung durch Relaxierung und tiefe Sedierung in der frühen Phase des ARDS. Es existieren mehrere Metaanalysen [364–367] zu Studien, welche Beatmungsmodi, die eine Spontanatmung in In- und Exspiration zulassen (vornehmlich APRV – Airway Pressure Release Ventilation). Diese zeigen für das ARDS, dass diese Form der Beatmung im Vergleich zu einer mandatorischen Beatmung die Mortalität um 5–15 % statistisch signifikant senken kann.

### 5.6 Nichtinvasive Atmungsunterstützung

#### 5.12/Empfehlung/Modifiziert (Stand 2025)

- Empfehlungsgrad: Schwach
- Wir schlagen vor, bei Patienten mit Sepsis oder septischem Schock und milder bis moderater akuter hypoxämischer respiratorischer Insuffizienz (PaO_2_/FiO_2_: 100–300) primär eine nichtinvasive Atmungsunterstützung (NIV, CPAP oder HFNO) anzuwenden.
- Adoption der S3-Leitlinie Beatmung*
- Qualität der Evidenz:
- Mortalität: Niedrig ⊕⊕⊝⊝
- Intensiv-Liegedauer: Niedrig ⊕⊕⊝⊝
- Literatur: [325, 368–371]
- Konsensstärke: 100 %
*Die Leitlinie war zum Zeitpunkt der Veröffentlichung noch nicht publiziert.

Wir schließen uns der Empfehlung der S3-Leitlinie „Invasive Beatmung und Einsatz extrakorporaler Verfahren bei akuter respiratorischer Insuffizienz“ [4] an. Das systematische Review mit Netzwerk-Metaanalyse von Pitre und Kollegen [368] ist hier eine zentrale Arbeit. Es wurde die nichtinvasive Beatmung mit Helm, nichtinvasive Beatmung mit Maske und HFNO-Therapie im Vergleich zur Standard-Sauerstofftherapie untersucht. CPAP-Therapie mit Helm, Bilevel-Therapie mit Helm und HFNO reduzieren das absolute Mortalitätsrisiko jeweils um 23,1 % (hohe Sicherheit, dass der wahre Effekt mit dem errechneten Effekt übereinstimmt), 12,9 % (moderate Sicherheit) und 6,3 % (moderate Sicherheit). Das absolute Risiko für eine invasive Beatmung sinkt unter HFNO um 10,3 % (hohe Sicherheit), unter Masken-Bilevel-Therapie um 9,9 % (hohe Sicherheit) und unter Helm-CPAP bzw. Helm-Bilevel um jeweils 30,6 % und 35,1 % bei moderater Sicherheit für den wahren Behandlungseffekt [368]. Diese Empfehlung ist in Übereinstimmung mit der japanischen ARDS-Leitlinie [325] und der deutschen S2k-Leitlinie „Nichtinvasive Beatmung als Therapie der akuten respiratorischen Insuffizienz“ [371].

### 5.7 Weaning

#### 5.13/Empfehlung/Bestätigt (Stand 2025)

- Empfehlungsgrad: Stark
- Wir empfehlen bei Patienten mit Sepsis oder septischem Schock, die länger als 24 h invasiv beatmet wurden, ein Protokoll zur Entwöhnung von der invasiven Beatmung (Weaning-Protokoll) anzuwenden, um standardisiert die Bewertung der Entwöhnungsbereitschaft (Readiness to Wean) zu evaluieren, die Spontanatmungsversuche (Spontaneous Breathing Trial) durchzuführen und die Kriterien zur Beendigung der invasiven Beatmung bzw. Extubation/Dekanülierung zu überprüfen.
- Adoption der S3-Leitlinie Beatmung*
- Qualität der Evidenz:
- Beatmungsdauer: Moderat ⊕⊕⊕⊝
- Literatur: [325]
- Konsensstärke: 100 %
*Die Leitlinie war zum Zeitpunkt der Veröffentlichung noch nicht publiziert.

Diese Empfehlung wurde sowohl von uns als auch von der S3-Leitlinie „Invasive Beatmung und Einsatz extrakorporaler Verfahren bei akuter respiratorischer Insuffizienz“ [4] nicht abgeändert, da es keine neuen hochwertigen Studien gibt. Die japanische Leitlinie „ARDS Clinical Practice Guideline 2021“ [325] hat diesbezüglich die vorliegende Evidenz in einer Metaanalyse untersucht. Die Krankenhaussterblichkeit und die Krankenhausmortalität bleiben von einem Weaning-Protokoll unbeeinflusst. Allerdings verkürzt ein Weaning-Protokoll die Beatmungsdauer um mehr als einen Tag im Mittel (MD −29,33; CI −47,98 bis −10,69) und zeigt einen Trend ohne statistische Signifikanz für ein vermindertes Mortalitätsrisiko (RR 0,73; CI 0,54 bis 1,01) [325].

### 5.8 Bauchlagerung

#### 5.14/Empfehlung/Bestätigt (Stand 2025)

- Empfehlungsgrad: Stark
- Wir empfehlen, bei invasiv beatmeten Patienten mit Sepsis oder septischem Schock und ARDS sowie Einschränkung der arteriellen Oxygenierung (PaO_2_/FiO_2_ < 150 mm Hg) eine Bauchlagerung durchzuführen.
- Adoption der S3-Lagerung und Mobilisation von kritisch Erkrankter auf Intensivstation
- Evidenzgrad: 1
- Literatur: [372–374]
- Konsensstärke: 100 %

Analog zur S3-Leitlinie „Invasive Beatmung und Einsatz extrakorporaler Verfahren bei akuter respiratorischer Insuffizienz“ [4] schließen wir uns der Empfehlung der aktuellen S3-Leitlinie „Lagerungstherapie und Mobilisation von kritisch Erkrankten auf Intensivstationen“ [6] an. Bei der Bauchlage wird der Patient um 180 ° in seiner Längsachse gedreht. Ziel der Bauchlagerung ist es, das Ventilations-Perfusions-Verhältnis zu verbessern und somit den Gasaustausch. Eine der zentralen Arbeiten ist die PROSEVA-Studie [375], die bei einem Oxygenierungs- bzw. Horovitz-Index von kleiner 150 mm Hg durch die Bauchlage eine Halbierung der Mortalität beim schweren ARDS erreichen konnte. Die Evidenz der S3-Leitlinie „Lagerungstherapie und Mobilisation von kritisch Erkrankten auf Intensivstationen“ [6] wird durch eine aktuelle Metaanalyse randomisierter kontrollierter Studien gestützt, die eine statistisch signifikante Mortalitätsreduktion für die Bauchlage zeigen, bei allerdings erhöhtem Risiko für Druckulzerationen [376].

#### 5.15/Empfehlung/Modifiziert (Stand 2025)

- Empfehlungsgrad: Stark
- Wir empfehlen, bei Patienten mit Sepsis oder septischem Schock eine Bauchlagerung über mindestens 12 h, vorzugsweise 16 h durchzuführen. Wir empfehlen, die Bauchlagerung frühzeitig zu erwägen und nach Indikationsstellung unverzüglich umzusetzen.
- Adoption der S3-Lagerung und Mobilisation von kritisch Erkrankter auf Intensivstation
- Evidenzgrad: 1
- Literatur: [374]
- Konsensstärke: 100 %

Analog zur S3-Leitlinie „Invasive Beatmung und Einsatz extrakorporaler Verfahren bei akuter respiratorischer Insuffizienz“ [4] schließen wir uns der Empfehlung der aktuellen S3-Leitlinie „Lagerungstherapie und Mobilisation von kritisch Erkrankten auf Intensivstationen“ [6] an. Die Dauer der Maßnahme beeinflusst maßgeblich deren Effektivität. Die Metaanalyse von Moran and Graham 2021 [374] arbeitet dies mittels Metaregression heraus. Die Arbeit zeigt einen Überlebensvorteil ab 12 h kontinuierlicher Bauchlagerung mit einer 2–4 % relativen Risikoreduktion der Mortalität für jede zusätzliche kontinuierliche Stunde in Bauchlage [374].

### 5.9 ECMO

#### 5.16/Empfehlung/NEU (Stand 2025)

- Empfehlungsgrad: Schwach
- Wir schlagen vor, den Einsatz einer venovenösen ECMO bei Patienten mit Sepsis oder septischem Schock und schwerem ARDS nur nach Ausschöpfen der konservativen Therapiemaßnahmen, einschließlich der Durchführung einer Bauchlagerung, und fortbestehender schwerer Gasaustauschstörung zu erwägen.
- Adoption der S3-Leitlinie Beatmung^2^
- Qualität der Evidenz:
- Mortalität: Sehr niedrig ⊕⊝⊝⊝
- Literatur: [323, 351, 377–382]
- Konsensstärke: 100 %

Wir schließen uns der Empfehlung der S3-Leitlinie „Invasive Beatmung und Einsatz extrakorporaler Verfahren bei akuter respiratorischer Insuffizienz“ [4] und der klinischen Praxisleitlinie der American Thoracic Society [351] an. Die VV-ECMO-Therapie ist eine Ressourcen-intensive Therapie. Für die Analyse von Nutzen und Schaden stehen nur das EOLIA-Trial [378] und das CESAR-Trial [381] zur Verfügung, aufgrund der vorhandenen, aber nicht sicher abschätzbaren Heterogenität [380] wurde auf die Nutzung von Metaanalysen verzichtet und nur die einzelnen Studien bewertet. Der differenzierte Einsatz im schweren ARDS kann die Mortalität senken, jedoch bleibt bisher unklar, bei welcher Subgruppe der Patienten mit schwerem ARDS der positive Effekt auftritt. In der EOLIA-Studie wies die ECMO-Kohorte ein höheres Risiko für Blutungen, Transfusionsbedarf und Thrombozytopenie auf [381, 382].

## 6. Nierenersatztherapie

Das folgende Kapitel ist eine Adoption der aktuellen S3-Leitlinie „Nierenersatztherapie in der Intensiv- und Notfallmedizin“ (AWMF-Reg.-Nr.: 040–017) [5].

Die in der S3-Leitlinie abgegebenen Empfehlungen zu Patienten in der Intensivmedizin betreffen erwachsene Patienten mit AKI mit und ohne Nierenersatztherapie (NET). Patienten mit akuter Nierenschädigung (Acute Kidney Injury – AKI) umfassen häufig auch Patienten mit Sepsis, septischem Schock und damit einem septischem AKI. Häufig befassen sich Studien auch ausschließlich mit septischem AKI als häufigster Ursache eines AKI.

Im folgenden Abschnitt werden einige wesentlichen Empfehlungen der Leitlinienkommission, die v. a. für Patienten mit Sepsis oder septischem Schock wichtig erscheinen, zusammengefasst. Für eine detaillierte Bewertung der zugrunde liegenden Evidenz sowie weitere Empfehlungen wird auf die Langfassung der S3-Leitlinie „Nierenersatztherapie in der Intensiv- und Notfallmedizin“ (https://register.awmf.org/de/leitlinien/detail/040-017) verwiesen.

### 6.1 Indizierung und Durchführung einer extrakorporalen Nierenersatztherapie bei Intensivpatienten

Die Indizierung und Durchführung einer extrakorporalen Nierenersatztherapie bei Intensivpatienten ist eine aufwendige und invasive Prozedur in einem komplexen Behandlungsumfeld. Aufgrund der großen Zahl an betroffenen Patienten ist sie eine der Kernaufgaben moderner Intensivmedizin. Zusätzlich zur extrakorporalen Nierenersatztherapie sind regelhaft weitere apparative und/oder medikamentöse Verfahren zur Unterstützung anderer Organsysteme notwendig, da eine AKI bei Intensivpatienten meist durch andere Erkrankungen hervorgerufen wird und häufig Bestandteil eines Mehrorganversagens ist. Daher muss die Nierenersatztherapie in das ganzheitliche Behandlungskonzept der Intensivpatienten integriert werden. Hierbei ist auch darauf zu achten, dass die Nierenersatztherapie kompatibel zu anderen apparativen Organunterstützungssystemen angewendet wird. Basierend auf den oben aufgeführten Punkten und der Musterweiterbildungsordnung können daher Intensivmediziner und/oder Nephrologen ein extrakorporales Nierenersatzverfahren bei Intensivpatienten indizieren und durchführen. Es gibt keine Studien, die untersucht haben, ob eine fachspezifische Indizierung und Durchführung eines extrakorporalen Nierenersatzverfahrens bei Intensivpatienten das Outcome beeinflusst.

#### 6.1/Empfehlung/NEU (Stand 2025)

- EK
- Wir empfehlen, dass extrakorporale Nierenersatzverfahren auf Intensivstationen ausschließlich durch Fachärzte mit der Zusatzbezeichnung „Intensivmedizin“ und/oder Nephrologen indiziert und durchgeführt werden.
- Konsensstärke: 100 %

### 6.2 Start der Nierenersatztherapie bei AKI in der Intensivmedizin

Der Beginn einer Nierenersatztherapie bei lebensbedrohlichen Komplikationen ist unumstritten. Unklar ist, ob durch frühen Behandlungsbeginn – mit dem Ziel einer Vermeidung von lebensbedrohlichen Komplikationen und der Gewährleistung einer stabileren metabolischen Stoffwechsellage – eine Reduktion der Sterblichkeit erreicht werden kann.

*Absolute Indikationen:* In der Tab. 6 werden lebensbedrohliche Veränderungen als absolute Indikationen für eine Nierenersatztherapie (NET) aufgeführt, die auf klinischem Konsens basieren.

**Tab. 6:** Lebensbedrohliche Veränderungen als absolute Indikationen für eine Nierenersatztherapie (NET)

Absolute Indikationen	Richtwerte	Anmerkungen
Hyperkaliämie	> 6,0 mmol/l	Therapieresistent (ohne NET)
Azidose	pH < 7,2	Therapieresistent (ohne NET) und durch AKI bedingt
Volumenüberladung	Ödeme, pulmonalvenöse Stauung	Therapieresistent (ohne NET)
Urämie	Klinik	u. a. Bewusstseinsstörungen

#### 6.2/Empfehlung/NEU (Stand 2025)

- EK
- Wir empfehlen, bei Patienten mit Sepsis oder septischem Schock und lebensbedrohlichen Veränderungen des Flüssigkeits‑, Säure-Basen- oder Elektrolythaushaltes unverzüglich mit einer Nierenersatztherapie zu beginnen.
- Adoption der S3-Leitlinie Nierenersatztherapie
- Konsensstärke: 100 %

#### 6.3/Empfehlung/NEU (Stand 2025)

- Empfehlungsgrad: Schwach
- Wir schlagen vor, bei Patienten mit Sepsis oder septischem Schock ein Nierenersatzverfahren ohne weiteres Zuwarten zu beginnen, wenn aufgrund der klinischen Situation, des Krankheitsverlaufes und/oder der Vorerkrankungen eine Nierenersatztherapie bei AKI zu erwarten ist.
- Adaptation der S3-Leitlinie Nierenersatztherapie/Erstellung von Evidenzprofilen (GRADE)
- Qualität der Evidenz:
- Mortalität (nach 30 Tagen): Moderat ⊕⊕⊕⊝
- Renale Erholung: Niedrig ⊕⊕⊝⊝
- Literatur: Fayad et al. 2022 [383]
- Konsensstärke: 100 %

#### 6.4/Empfehlung/NEU (Stand 2025)

- Empfehlungsgrad: Schwach
- Wir schlagen vor, bei nichtlebensbedrohlichen Veränderungen und Unklarheit, ob eine Nierenersatztherapie zu erwarten ist, konservative Maßnahmen zur Vermeidung eines Nierenersatzverfahrens unter regelmäßiger Reevaluation durchzuführen.
- Adaptation der S3-Leitlinie Nierenersatztherapie/Abweichung des Empfehlungsgrades und Erstellung von Evidenzprofilen (GRADE)
- Qualität der Evidenz:
- Mortalität: Moderat ⊕⊕⊕⊝
- Renale Erholung: Niedrig ⊕⊕⊝⊝
- Literatur: Fayad et al. 2022 [1]
- Konsensstärke: 100 %

Zum Thema Start eines Nierenersatzes wurden 22 systematische Übersichtsarbeiten (Systematic Reviews, davon 2 Cochrane-Reviews), 19 randomisierte klinische Studien (Randomized Controlled Trials – RCTs) sowie 63 Observations- und retrospektive Studien identifiziert (s. Evidenzreport der S3-Leitlinie Nierenersatztherapie). In allen großen RCTs der letzten Jahre waren Patienten mit Sepsis oder septischem Schock, in allerdings unterschiedlichem Ausmaß, eingeschlossen (Gaudry et al. 2016 [384] 80 %, Barbar et al. 2018 [385] 100 %, Bagshaw et al. 2020 [386] 58 %). Die Ergebnisse erscheinen damit gut auf Patienten mit septischem AKI und Nierenersatzpflichtigkeit übertragbar zu sein. Ein direkter Vergleich dieser Studien, insbesondere der randomisierten kontrollierten Studien, ist vor allem wegen der uneinheitlichen Definition von „früh“ und „spät“ erschwert.

*Definition von früh und spät:* Bei den seit 2012 durchgeführten RCTs wurden vornehmlich die KDIGO-Kriterien für die Einteilung von früh und spät verwendet, dies jedoch sehr unterschiedlich. Die Definition von „früh“ umfasste das KDIGO-Stadium 2 oder 3. Im engeren Sinne wurde damit aber primär nicht die Frage beantwortet, wann in der zeitlichen Dynamik einer schweren Intensiverkrankung bzw. Sepsis, sondern ab welchem Grad der Nierenfunktionseinschränkung ein Nierenersatzverfahren gestartet werden sollte.

*Sterblichkeit und Verfahrenswahl:* Aktuell ist noch nicht sicher bekannt, ob die unterschiedlichen Nierenersatzverfahren (CRRT, SLEDD, IHD) einen Einfluss auf die Sterblichkeit der Patienten haben. Insbesondere in den großen RCTs (Bagshaw et al. 2020 [386]; Barbar et al. 2018 [385]; Gaudry et al. 2021 [387]; Gaudry et al. 2016 [384]) wurden unterschiedliche Nierenersatzverfahren durchgeführt. Eine erst kürzlich publizierte Sekundäranalyse der STARRT-AKI-Studie (Wald et al. 2023 [388]) zeigte, dass die IHD mit einer höheren Mortalität assoziiert war.

*KDIGO-Stadium:* Das KDIGO-Stadium (Kreatinin, Diuresemenge) allein scheint keine valide Entscheidungsgrundlage für den Beginn eines Nierenersatzverfahrens zu sein. Aus diesem Grunde haben die aktuellen großen RCTs neben dem KDIGO-Stadium weitere klinische Faktoren hinzugezogen, um die Entscheidung für den Beginn eines Nierenersatzverfahrens zu treffen. Dies gilt umso mehr, da weitere klinische Kontextfaktoren das Behandlungsergebnis der Patienten beeinträchtigen können. So war der sehr frühe Beginn eines Nierenersatzverfahrens bei Patienten mit hohem Schweregrad der Erkrankung von Überlebensvorteil (Zarbock et al. 2016 [389]), während ein sehr später Beginn mit einer Übersterblichkeit einherging (Hazard Ratio, 1,65; 95 % CI 1,09 bis 2,50; *P* = 0,0018) (Gaudry et al. 2021 [387]).

*Systematic Review: *Ein Vergleich der Sterblichkeit zwischen frühem und spätem Beginn der Nierenersatztherapie erfolgte in einer Cochrane-Analyse (Fayad et al. 2022 [383]). Das RCT von Gaudry et al. 2021 [387] wurde nicht in die letzte Cochrane-Analyse eingeschlossen, da sich die Zeitpunkte der Randomisierung erheblich von den anderen RCTs unterschieden. Es wurde dort ein später mit einem sehr späten Zeitpunkt verglichen.

Neben der Cochrane-Analyse wurden zusätzlich 16 systematische Übersichtsarbeiten und 4 Metaanalysen betrachtet. Davon zeigten die systematischen Übersichtsarbeiten (Karvellas et al. 2011 [390]; Liu et al. 2014 [391] und eingeschränkt auch Besen et al. 2019 [392]) einen Überlebensvorteil bei frühem Einsatz einer Nierenersatztherapie. Diese Schlussfolgerung beruhte vornehmlich auf den Ergebnissen von Observations- und retrospektiven Studien (AMSTAR-Qualitätsbewertung: „critically low“). Im Gegensatz dazu zeigen alle anderen systematischen Übersichtsarbeiten keinen Überlebensvorteil: Risiko, zu versterben an Tag 30: RR 0,97; 95 % CI 0,87 bis 1,09, I^2^ = 29 %, geringe Evidenz (Fayad et al. 2022 [383]); Risiko, zu versterben nach Tag 30: RR 0,99, 95 % CI 0,92 bis 1,07, I^2^ = 6 %, moderate Evidenz (Fayad et al. 2022 [383]). Jedoch ist die Aussagekraft der Studien eingeschränkt, da die Heterogenität sehr hoch ist. Lediglich zwei systematische Übersichtsarbeiten (Gaudry et al. 2020 [393]; Zhang et al. 2020 [394]) besaßen eine Heterogenität von nur 0–2 %, diese sind jedoch als AMSTAR „critically low“ (3 systematische Übersichtsarbeiten) oder „low“ (1 systematische Übersichtsarbeit) bewertet.

Bei der Bewertung der neueren systematischen Übersichtsarbeiten, welche die RCTs inkludierten, ist zudem kritisch anzumerken, dass viele Patienten der Spätgruppe gar keine Nierenersatztherapie erhielten. Insbesondere in dem RCT von Bagshaw et al. 2020 [386] haben nur 61,8 % der Patienten in der Spätgruppe ein Nierenersatzverfahren erhalten. Ein direkter Vergleich zu Patienten mit durchgeführter Nierenersatztherapie ist methodisch zwar zulässig, beantwortet jedoch nicht die eigentliche Fragestellung.

*Weitere Studien und offene Fragen: *Es kann zusammengefasst werden, dass die KDIGO-Kriterien allein nicht für die Indikationsstellung einer Nierenersatztherapie geeignet sind.

In weiteren Studien sind deshalb folgende Fragen zu beantworten:

1. Wie können aus der Gruppe aller AKI-Patienten diejenigen identifiziert werden, die im Verlauf tatsächlich eine Nierenersatztherapie (mit absoluter Indikation) benötigen?
2. Profitieren die Patienten, die eine absolute Indikation im Verlauf entwickeln werden, von einem frühzeitigen Beginn einer Nierenersatztherapie?

Zur Beantwortung der o. g. Fragen gibt es bereits Studien, die sich mit der Etablierung neuer strategischer Konzepte beschäftigen. So wurden zum Beispiel unterschiedliche Biomarker und deren Nutzen für die Indikationsstellung für den Beginn einer Nierenersatztherapie oder die Kombination eines Biomarkers mit einem funktionellen Test (Furosemid-Stresstest) untersucht (siehe Tab. 1.16 des Evidenzreportes der S3-Leitlinie „Nierenersatz in der Intensiv- und Notfallmedizin“). Praxisrelevante Ergebnisse liegen jedoch aufgrund bisher geringer Evidenz noch nicht vor.

### 6.3 Diffusion versus Konvektion

Prinzipiell sind Dialyseverfahren von Filtrationsverfahren zu unterscheiden. Verwenden Dialyseverfahren die Diffusion als treibende Kraft zur Entfernung von Stoffen aus dem Blut, kommt bei Filtrationsverfahren der Konvektion die entscheidende Bedeutung zu. Dieses Kapitel beschränkt sich auf den Vergleich der Diffusion und Konvektion. Das Thema der „Adsorption“ von Stoffen an spezielle extrakorporale Oberflächen und spezieller Membranen wird nicht behandelt, da es sich nicht um ein primäres Nierenersatzverfahren handelt.

#### 6.5/Empfehlung/NEU (Stand 2025)

- Empfehlungsgrad: Schwach
- Wir schlagen vor, bei Patienten mit einer Sepsis und Indikation zur Nierenersatztherapie diffusive Nierenersatzverfahren oder konvektive Verfahren bzw. eine Kombination aus diffusiven und konvektiven Verfahren gleichwertig einzusetzen.
- Adaptation der S3-Leitlinie Nierenersatztherapie/Erstellung von Evidenzprofilen (GRADE)
- Qualität der Evidenz:
- Mortalität: Sehr niedrig ⊕⊝⊝⊝
- Renale Erholung: Sehr niedrig ⊕⊝⊝⊝
- SAE: Sehr niedrig ⊕⊝⊝⊝
- Literatur: Côté et al. 2022 [395]
- Konsensstärke: 100 %

Die vorliegende Literatur erlaubt es nicht, Diffusion oder Konvektion als ein überlegenes Verfahren auf der Intensivstation einzuordnen. Alle Verfahren ermöglichen eine adäquate metabolische Kontrolle. Die meisten Studien untersuchten hierbei Patienten mit Sepsis oder septischem Schock. Nur für den Outcome-Parameter „Filterstandzeiten“ konnten zwei kleinere RCTs (Xu et al. 2022 [396], *n* = 60; und Mann et al. 2023 [397], *n* = 161) einen signifikanten Vorteil für die Diffusion gegenüber reiner Konvektion mit entsprechend längeren Filterstandzeiten aufzeigen.

*Systematic Review: *Die systematische Übersichtsarbeit von Côté et al. 2022 [395] mit insgesamt 615 AKI-Patienten, die ausschließlich mit intermittierenden Verfahren behandelt wurden, konnte keinen Unterschied zwischen diffusiven und konvektiven intermittierenden Verfahren für die Endpunkte Mortalität, renale Erholung oder hämodynamische Instabilität nachweisen.

*Anmerkungen: *Rein konvektive Verfahren können aufgrund einer Hämokonzentration im Filter zu einer höheren Rate an Filterclotting und damit zu kürzeren Filterstandzeiten führen.

*Sepsis und Clearance von Mittelmolekülen: *Bei Patienten mit einer Sepsis haben konvektive Verfahren theoretisch Vorteile durch eine höhere Mittelmolekülclearance, die zur Elimination proinflammatorischer Mediatoren beitragen könnte. In mehreren Studien wurde jedoch gezeigt, dass die Mittelmolekülclearance für die konventionelle Hämofiltration, die „High-Volume-“ und „High-Cut-off-Hämofiltration“ bei Patienten mit Sepsis bzw. kritisch Kranken mit AKI gleich ist.

### 6.4 Kontinuierliche versus intermittierende Nierenersatzverfahren

Eine Nierenersatztherapie kann bei kritisch kranken Patienten als kontinuierliche Nierenersatztherapie (CRRT), intermittierende Nierenersatztherapie/Hämodialyse (IHD) oder als prolongierte intermittierende Nierenersatztherapie (Prolonged Intermittent Renal Replacement Therapy, PIRRT) durchgeführt werden. Hinsichtlich des prolongierten Verfahrens ist in Deutschland der Begriff Sustained Low Efficient Daily Dialysis (SLEDD) anstelle PIRRT gebräuchlicher und wird daher in dieser Leitlinie verwendet. Die Verfahren unterscheiden sich durch die Dauer der Behandlung pro Tag und die Intensität der Blutreinigung pro Zeiteinheit. Eine CRRT soll unter idealen Bedingungen 24 h pro Tag unterbrechungsfrei laufen. Eine IHD wird in der Regel für 4–6 h pro Tag durchgeführt. Die Durchführung von SLEDD-Verfahren zeigt in Studien eine erhebliche Variabilität. Es wurden Behandlungszeiten von 6 bis 12 h – in einigen Fällen auch darüber hinaus – beschrieben.

#### 6.6/Empfehlung/Modifiziert (Stand 2025)

- Empfehlungsgrad: Schwach
- Wir schlagen vor, dass zur Nierensatztherapie der AKI bei Patienten mit Sepsis kontinuierliche oder intermittierende Verfahren gleichwertig eingesetzt werden können.
- Bemerkung: Bei der Auswahl des Verfahrens soll jedoch die individuelle klinische Situation des Patienten berücksichtigt werden, die zu einer Bevorzugung eines kontinuierlichen oder intermittierenden Verfahrens führen kann.
- Adaptation der S3-Leitlinie Nierenersatztherapie/Erstellung von Evidenzprofilen (GRADE)
- Qualität der Evidenz:
- CRRT vs. IHD
- Mortalität: Niedrig ⊕⊕⊝⊝
- Literatur: Ye et al. 2021 [398]
- Konsensstärke: 100 %
- a Bei den individuellen klinischen Situationen sind insbesondere klinische Besonderheiten, wie Hirndruck, Leberversagen, Flüssigkeitsüberschuss, Thrombopenie und die hämodynamische Stabilität zu berücksichtigen.

Der Zeitpunkt der Erhebung der Mortalität variierte zwischen den Studien. Der kürzeste Erfassungszeitraum enthielt nur die Intensivstationsmortalität, in der Mehrzahl der Studien wird die Krankenhaussterblichkeit erfasst, der längste Erfassungszeitraum betrug 90 Tage. In der Cochrane-Analyse (Rabindranath et al. 2007 [399]) von 15 RCTs mit 1550 Patienten fand sich beim Vergleich CRRT zu IHD eine RR von 1,01 (95 % CI 0,92 bis 1,12), in der letzten publizierten systematischen Übersichtsarbeit (Ye et al. 2021 [398]) von 30 RCTs mit 3774 Patienten fand sich eine RR von 1,04 (95 % CI 0,93 bis 1,18). Die systematischen Übersichtsarbeiten, die sich auf die *Auswertung von RCTs *beschränkten*,* konnten keinen Unterschied in der Mortalität zwischen CRRT und IHD nachweisen. Nur in der Analyse retrospektiver und observationaler Studien zeigte sich ein moderater Mortalitätsvorteil für die CRRT.

Sechs systematische Übersichtsarbeiten untersuchten die Mortalität unter Verwendung von einer SLEDD im Vergleich zu einer CRRT. Hier ergibt sich ein heterogenes Bild. Vier systematische Übersichtsarbeiten zeigten keine Unterschiede, hingegen zeigten zwei systematische Übersichtsarbeiten einen leichten Überlebensvorteil für die SLEDD (Ye et al. 2021 [398]: RR 1,06; 95 % CI 0,85 bis 1,33; Zhang et al. 2015 [400]: RR 0,86; 95 % CI 0,74 bis 1,00; Kovacs et al. 2017 [401]: RR 1,21; 95 % CI 1,02 bis 1,43; I^2^ 47 %, *p* = 0,03). Die Datenlage kann insgesamt als nicht hinreichend und so heterogen eingeschätzt werden, dass eine sichere Schlussfolgerung nicht möglich ist.

#### 6.7/Empfehlung/NEU (Stand 2025)

- Empfehlungsgrad: Schwach
- Wir schlagen vor, bei hämodynamisch instabilen Patienten mit Sepsis und Nierenersatztherapie bei AKI ein kontinuierliches oder verlängert intermittierendes Verfahren einzusetzen.
- Adaptation der S3-Leitlinie Nierenersatztherapie/Erstellung von Evidenzprofilen (GRADE)
- Qualität der Evidenz:
- Hämodynamische Instabilität: Sehr niedrig ⊕⊕⊝⊝
- Hypotonie: Niedrig ⊕⊕⊝⊝
- Literatur: Rabindranath et al. 2007 [399]
- Konsensstärke: 100 %

In den systematischen Übersichtsarbeiten wurde die Frage einer hämodynamischen Instabilität unter der jeweiligen Nierenersatzmodalität untersucht (hämodynamische Instabilität als SAE/Outcome Parameter). In einer Cochrane-Analyse von Rabindranath et al. 2007 [399] wurden 15 RCTs mit 1550 Patienten analysiert. Es konnte kein Unterschied in der Anzahl der Patienten mit (allerdings schlecht definierter) hämodynamischer Instabilität (RR 0,48; 95 % CI 0,10 bis 2,28; *n* = 205) oder mit (variabel definierter) Hypotonie (RR 0,92; 95 % CI 0,72 bis 1,16; *n* = 514) zwischen CRRT und IHD gefunden werden. Allerdings war der mittlere arterielle Blutdruck am Ende der Behandlung unter CRRT signifikant höher als unter IHD. Die Anzahl der Patienten, die eine Eskalation der Vasopressor-Therapie benötigten, war bei der CRRT im Vergleich zur IHD niedriger. Von den 15 eingeschlossenen Studien lieferten nur zwei Daten zur hämodynamischen Instabilität (*n* = 205), drei Studien Daten zur Häufigkeit von Hypotension (*n* = 514) und drei Studien Daten zur Eskalation einer Vasopressor-Therapie (*n* = 149). Unterschiede in der Noradrenalin-Dosierung fanden sich nicht (2 Studien, *n* = 69).

Aktuelle Evidenzen deuten darauf hin, dass eine IHD im individuellen Falle mit mehr Hypotonien und mehr Vasopressor-Einsatz verbunden sein kann (hämodynamische Instabilität als SAE). Eine hämodynamische Instabilität war entsprechend retrospektiven Untersuchungen der häufigste Grund für das Scheitern eines intermittierenden Verfahrens. Aus Gründen der einfacheren Steuerbarkeit, der höheren Gefahr eines Disäquilibriums eines hocheffizienten intermittierenden Verfahrens und tradierter klinischer Erfahrung empfehlen wir trotz geringer Evidenz in der Subgruppe der hämodynamisch deutlich instabilen Patienten die Durchführung eines verlängerten oder kontinuierlichen Verfahrens (hämodynamische Instabilität als Indikationskriterium für eine CRRT).

### 6.5 Regionale und systemische Antikoagulation

Bei einer extrakorporalen Nierenersatztherapie wird das Patientenblut einer Vielzahl von Fremdoberflächen (Schlauchsystem, Luftkontakte, Kapillaren) ausgesetzt mit der Konsequenz, dass die Hämostase aktiviert sowie eine Inflammation induziert wird. Voraussetzung für eine erfolgreiche extrakorporale Nierenersatztherapie ohne frühzeitige und vor allem ungeplante Therapieunterbrechung ist daher eine effektive Antikoagulation des extrakorporalen Kreislaufes.

Grundsätzlich unterscheidet man eine systemische von einer regionalen Antikoagulation. Die systemische Antikoagulation wird in der Regel durch unfraktioniertes Heparin (aktueller Standard bei der intermittierenden Hämodialyse) oder fraktioniertes Heparin erzielt, alternativ – bei Vorliegen einer Heparin-induzierten Thrombozytopenie Typ II – durch den direkten Thrombin-Inhibitor Argatroban oder das Heparinoid Danaparoid.

Eine regionale Antikoagulation wird aktuell im Wesentlichen durch Citrat, dem Salz der Zitronensäure, erreicht. Ziel der regionalen Antikoagulation ist eine erfolgreiche extrakorporale Therapie, ohne die systemische Hämostase des Patienten zu beeinflussen.

Beim Vergleich systemische versus regionale Antikoagulation sind wichtige Outcome-Parameter neben den Filterstandzeiten die Mortalität der Patienten, die Blutungsraten sowie die Transfusionsfrequenz.

#### 6.8/Empfehlung/NEU (Stand 2025)

- Empfehlungsgrad: Stark
- Wir empfehlen, bei Patienten mit Sepsis oder septischem Schock eine regionale Citrat-Antikoagulation oder eine systemische Heparin-Antikoagulation des extrakorporalen Blutkreislaufs gleichwertig einzusetzen.
- Adaptation der S3-Leitlinie Nierenersatztherapie/Erstellung von Evidenzprofilen (GRADE)
- Qualität der Evidenz:
- Mortalität [402]: Moderat ⊕⊕⊕⊝
- Filterstandzeiten [402]: Moderat ⊕⊕⊕⊝
- Blutungsrate [403]: Moderat ⊕⊕⊕⊝
- Transfusionen [403]: Niedrig ⊕⊕⊝⊝
- Literatur: Jacobs et al. 2023 [403], Tsujimoto et al. 2020 [402]
- Konsensstärke: 100 %

Eine Reduktion der Mortalität konnte in den einzelnen RCTs nicht nachgewiesen werden. Eine Ausnahme stellte die Untersuchung von (Oudemans-van Straaten et al. 2009 [404]) dar, in welcher unter Citrat im Vergleich zu Nadroparin eine reduzierte Mortalität gefunden wurde (45 % vs. 62 %; *p* = 0,03). Abweichend von anderen RCTs unterschied sich in dieser Studie die Filterlaufzeit nicht signifikant. Einschränkend ist zu sagen, dass die Mortalität kein primärer Endpunkt dieser Studie war.

*Systematic-Review-Analyse: *Sieben systematischen Übersichtsarbeiten seit 2015 wurden ausgewertet, darunter ein Cochrane-Report (Tsujimoto et al. 2020 [402]) sowie eine aktuelle systematische Übersichtsarbeit von Jacobs et al. 2023 [403]. Übereinstimmend kamen aktuelle systematische Übersichtsarbeiten (Bai et al. 2015 [405]; Liu et al. 2016 [406]; Jacobs et al. 2023 [403]; Qi et al. 2023 [407]) und der Cochrane-Report (Tsujimoto et al. 2020 [402]) zum Ergebnis, dass unter regionaler Citrat-Antikoagulation kein Mortalitätsvorteil festzustellen ist. Tsujimoto et al. 2020 [402] und Jacobs et al. 2023 [403] sahen in ihren Auswertungen ebenfalls keinen signifikanten Effekt auf die Erholung der Nierenfunktion unter regionaler Citrat-Antikoagulation.

Die Filterlaufzeiten sind in allen aktuellen systematischen Übersichtsarbeiten mit regionaler Citrat-Antikoagulation signifikant verlängert, daher ist eine gute Evidenz für höhere Filterlaufzeiten unter regionaler Citrat-Antikoagulation im Vergleich zu systemischer Heparinisierung anzunehmen.

In den aktuellen systematischen Übersichtsarbeiten traten konsistent unter regionaler Citrat-Antikoagulation signifikant weniger Blutungskomplikationen auf (siehe Evidenzreport). Nur in der systematischen Übersichtsarbeit von Jacobs et al. 2023 [403] wurde neben der Blutungs- auch die Transfusionshäufigkeit ausgewertet, die sich zwischen regionaler Citrat-Antikoagulation und systemischer Heparin-Gabe nicht unterschied (RR 1,02; 95 % CI 0,93 bis 1,12; *p* = 0,644). Warum der eindeutige Vorteil hinsichtlich Blutungen nicht auch in einer verminderten Transfusionshäufigkeit resultiert, ist nicht klar.

Der Einsatz von regionaler Citrat-Antikoagulation ist bei Patienten, bei denen der Einsatz von Heparin aufgrund von Kontraindikationen (z. B. HIT) nicht in Frage kommt, und bei Patienten mit erhöhtem Blutungsrisiko anzuwenden.

Durchgeführte Studien konnten zeigen, dass es trotz einer eingeschränkten hepatischen Citrat-Metabolisierung nicht signifikant häufiger zu einer toxischen Citrat-Akkumulation und einer begleitenden Azidose kam. Sehr hohe Laktatspiegel (> 4 mmol/l) zu Beginn einer CRRT oder eine verzögerte Laktat*clearance* bei Leberversagen wurden jedoch als Variablen beschrieben, die mit einer höheren Citrat-Akkumulation einhergingen (Khadzhynov et al. 2017 [408]). Damit spricht die aktuelle Studienlage nicht dafür, dass eine regionale Citrat-Antikoagulation unter Leberversagen oder Schock mit Laktatazidose als Kontraindikation per se zu betrachten ist. Jedoch sollte bei einer ausgeprägten, progredienten Laktatazidose im Rahmen eines Schocks oder schwerem Leberversagen, aufgrund einer möglichen Citrat-Akkumulation, keine regionale Citrat-Antikoagulation angewendet werden. Aufgrund der komplexen metabolischen Situation wird ein enges Monitoring der Citrat-Akkumulation und eine enggefasste Indikationsstellung empfohlen (nicht graduiert).

### 6.6 Adäquate Dosis eines Nierenersatzverfahrens

Die Intensität eines Nierenersatzverfahrens wird über die applizierte Dialyse- oder Filtrations-Dosis gesteuert.

*Dosisbegriff bei kontinuierlichen Nierenersatzverfahren:* Bei dem Dosisbegriff für *kontinuierliche Verfahren* wird die Dosis primär über die Menge des Effluats definiert. Hier müssen die unterschiedlichen Möglichkeiten der CRRT differenziert betrachtet werden.

1. *Kontinuierliche venovenöse Hämofiltration (CVVH)*: Hier ist die Konvektion der zugrunde liegende Mechanismus für die Elimination der gelösten Substanzen. Es wird eine Substituatlösung verwendet, um das filtrierte Volumen zu ersetzen. Das Substituat kann vor dem Filter (*Prädilution*) sowie nach dem Filter (*Postdilution*) gegeben werden.
  *Dosis (ml/kg/h)* *=* *(Substituatmenge* *+* *ggf. Citratflüssigkeit)* *+* *Volumenentzug/kgKG*
2. *Kontinuierliche venovenöse Hämodialyse (CVVHD):* Hier ist die Diffusion der zugrunde liegende Mechanismus. Hier wird keine Substituat-Lösung verwendet.
  *Dosis (ml/kg/h)* *=* *(Dialysatfluss* *+* *Volumenentzug)/kgKG*
3. *Kontinuierliche venovenöse Hämodiafiltration (CVVHDF):* Hier besteht der zugrunde liegende Mechanismus sowohl aus Konvektion als auch aus Diffusion, sodass sich die Dosis folgendermaßen berechnet.
  *Dosis (ml/kg/h)* *=* *(Substituatmenge* *+* *ggf. Citratflüssigkeit* *+* *Dialysatfluss* *+* *Volumenentzug)/kgKG*

Zu unterscheiden ist jedoch die *verordnete und tatsächliche Behandlungszeit*, die aufgrund von Unterbrechungen oder Fortsetzungen der Behandlungen sich unterscheiden können. Für den Dosisbegriff bei kontinuierlichen Verfahren wird üblicherweise verwendet:

- *Standarddosis*: 20–30 ml/kg/h Filtrat/Dialysatmenge plus Ultrafiltratmenge
- *Hohe Dosis*: 30–50 ml/kg/h Filtrat/Dialysatmenge plus Ultrafiltratmenge
- *Hochvolumen-Dosis* (in der Literatur als High-Volume-CRRT beschrieben): > 50 ml/kg/h Filtratmenge/Dialysatmenge plus Ultrafiltratmenge.

*Dosisbegriff bei intermittierenden Nierenersatzverfahren:* Zur Definition einer Dosissteigerung bei den intermittierenden Verfahren in der Intensivmedizin wird in den RCTs meist primär durch eine höhere, kumulative Dialysezeit angegeben. Dies kann durch eine verlängerte Dialysezeit eines einzelnen Verfahrens oder durch eine erhöhte Dialysefrequenz erreicht werden. In den intensivmedizinischen RCTs wurde meist die Frequenz der Dialysen erhöht, um eine höhere *Dosis* zu erreichen.

Physikalisch betrachtet führt auch ein erhöhter Dialysat- oder Blutfluss zu einer erhöhten Clearance der einzelnen Substanzen. Durchschnittliche Werte bei den intermittierenden Verfahren in der Intensivmedizin sind Blutflüsse von 200–300 ml/min und Dialysatflüsse von ca. 300–500 ml/min. Diese sind damit bereits so hoch, dass eine weitere Steigerung nur geringfügig zu einer höheren Clearance kleinmolekularer Substanzen beiträgt. Entsprechend sind folgende Dosisbegriffe zu unterscheiden:

- *Dialysen/Woche*: täglich, 3 Dialysen/Woche
- *Kt/V*: Harnstoffclearance (K) *Behandlungszeit (t)/Verteilungsvolumen Harnstoff (V)
- *Urea Reduction Rate (URR)*: Abnahme des Harnstoffs über die Dialysesitzung

Im intensivmedizinischen Setting wird eine intensive Dosis meist als tägliche Dialysen im Vergleich zu 3 Dialysen/Woche definiert. Im Vergleich hierzu werden bei chronisch dialysepflichtigen Patienten primär die Harnstoffclearance, das kt/V und die Urea Reduction Rate (URR) zur Messung der Effektivität eines Dialyseverfahrens und als Qualitätsmerkmal verwendet. Diese Parameter können rechnerisch prinzipiell auch für AKI-Patienten verwendet werden, sind jedoch primär für klinisch stabile Patienten mit gleichbleibender Organfunktion evaluiert. Bei den sich rasch verändernden Organfunktionen des kritisch kranken Patienten mit der erhöhten Gefahr eines Disäquilibriums und unterschiedlichen Anteilen einer endogenen und maschinellen Clearance empfehlen wir daher die Adäquatheit der Dialysedosis mittels labordiagnostischer Parameter (Kreatinin, Harnstoff, Phosphat, Säure-Basen-Status u. a. m) zu überprüfen.

#### 6.9/Empfehlung/NEU (Stand 2025)

- Empfehlungsgrad: Stark
- Wir empfehlen, bei Patienten mit Sepsis oder septischem Schock für ein kontinuierliches Nierenersatzverfahren eine mittlere Dosis von 20–25 ml/kg/h über die geplante Behandlungsdauer zu verabreichen.
- Adaptation der S3-Leitlinie Nierenersatztherapie/Erstellung von Evidenzprofilen (GRADE)
- Qualität der Evidenz:
- Mortalität (30 Tage): Niedrig ⊕⊕⊝⊝
- Renale Erholung: Moderat ⊕⊕⊕⊝
- Literatur: Fayad et al. 2016 [409]
- Konsensstärke: 100 %

Die hier empfohlene Dosis richtet sich nach aktueller bester Evidenz. Grundsätzlich ist zu beachten, dass die verschriebene Dosis („*prescribed dose*“) zumeist nicht der tatsächlich erzielten Dosis („*delivered dose*“) entspricht, da die Dosis von der effektiven Laufzeit abhängig ist und dementsprechend durch Unterbrechungen der Verfahren (z. B. durch Transport, Clotting, Rüstzeiten usw.) beeinflusst wird. Im klinischen Alltag wird daher die verschriebene Dosis etwas höher gewählt (z. B. 25–30 ml/h/kg), um eine bestimmte Zieldosis (z. B. 20–25 ml/h/kg) zu erreichen.

*Systematic-Review-Analyse: *Insgesamt wurden 7 systematische Übersichtsarbeiten identifiziert, von denen 6 in der AMSTAR-Bewertung als kritisch niedrig und ein Cochrane-Review als hoch graduiert wurden. Die Cochrane-Analyse von Fayad et al. 2016 [409] sowie alle systematischen Übersichtsarbeiten kamen übereinstimmend zu dem Ergebnis, dass eine höhere Dialysedosis (35 ml/kg/h) im Vergleich zu einer niedrigeren Dosis (20 ml/kg/h) die 30-Tage-Mortalität nicht verbesserte. Alle systematischen Übersichtsarbeiten zeigten eine hohe Gewichtung durch den Einschluss der ATN- (Palevsky et al. 2008 [410]) und RENAL- (Bellomo et al. 2009 [411]) Studien mit hoher Patientenzahl. Die Evidenz kann dennoch aufgrund der beiden aufwendigen hochqualitativen Studien, die beide zum gleichen Ergebnis kamen, als gut gelten: Mortalität 30 Tage nach Randomisierung: OR 0,92 (95 % CI 0,80 bis 1,06; *P* = 0,26).

#### 6.10/Empfehlung/NEU (Stand 2025)

- *Empfehlungsgrad: Schwach*
- *Wir schlagen vor, bei Patienten mit Sepsis oder septischem Schock eine kontinuierliche hochvolumige („high-volume“) Hämofiltration NICHT anzuwenden.*
- *Adaptation der S3-Leitlinie Nierenersatztherapie/Erstellung von Evidenzprofilen (GRADE)*
- *Qualität der Evidenz:*
- *Mortalität (ICU): Niedrig ⊕⊕⊝⊝*
- *Mortalität (28-Tage): Niedrig ⊕⊕⊝⊝*
- Literatur: *Borthwick* et al. 2017 [412]
- *Konsensstärke*: 100 %

Die Literaturrecherche zum Thema hochvolumige Hämofiltration bei septischen Patienten ergab 6 systematische Übersichtsarbeiten, 7 RCTs und 11 sonstige Studien. Die Leitliniengruppe hat dabei entschieden, die Grenze für hochvolumige Hämofiltration bei einer Dosis von ≥ 50 ml/kg/h zu setzen.

*Systematic-Review-Analyse:* Von den fünf identifizierten systematischen Übersichtsarbeiten wurden drei mit AMSTAR als kritisch niedrig, eine niedrig und eine als hoch bewertet (Cochrane-Analyse: Borthwick et al. 2017 [412]). Die vorliegenden systematischen Übersichtsarbeiten boten kein einheitliches Bild. Einerseits kamen Junhai et al. 2019 [413] und Huang et al. 2021 [414] zu dem Ergebnis, dass die Mortalität mit einer RR von 0,88 (95 % CI 0,81 bis 0,96; *P* = 0,004) sank (Junhai et al. 2019 [413]) bzw. sich das Überleben durch Anwendung einer hochvolumigen Hämofiltration in der Sepsis mit einer OR 1,66 (95 % CI 1,36 bis 2,01; *p* < 0,001) verbesserte (Huang et al. 2021 [414]). In beiden systematischen Übersichtsarbeiten waren jedoch keine Effekte auf die Multiorganversagen-Scores wie MODS und APACHE-II festzustellen. Alle anderen systematischen Übersichtsarbeiten zeigten keinen Überlebensvorteil für eine hochvolumige Therapie bei Sepsis-Patienten.

Die Angaben zu SAEs waren in den einzelnen Studien heterogen und unvollständig. Huang et al. 2021 [414] berechnete zwar einen Trend zu mehr Nebenwirkungen unter hochvolumigen Verfahren, das Ergebnis war jedoch mit einer OR 1,62 (95 % CI 1,2 bis 2,11; *P* = 0,24) nicht signifikant.

Die vorliegende Evidenz macht angesichts der heterogenen, eher kleinen Studien mit regionalem Bias eine fundierte Einordnung einer hochvolumigen Hämofiltration unmöglich. Dass eine erhöhte Elimination von Zytokinen und anderen Mittelmolekülen erfolgt, ist physikalisch plausibel und wird durch die Datenlage gestützt. Eine klinische Outcome-Verbesserung konnte für keine der untersuchten Populationen eindeutig nachgewiesen werden.

## 7. Ernährungstherapie

### 7.1/Empfehlung/Modifiziert (Stand 2025)

- Empfehlungsgrad: Schwach
- Wir schlagen vor, bei Patienten mit Sepsis oder septischem Schock nach hämodynamischer Stabilisierung eine frühe enterale oder parenterale Ernährung unter Monitoring der gastrointestinalen und metabolischen Toleranz durchzuführen.
- SSC-Leitlinienadaptation/Aktualisierung der Evidenz und Änderung des Empfehlungstextes
- Qualität der Evidenz:
- Mortalität: Niedrig ⊕⊕⊝⊝
- Literatur: [415]
- Konsensstärke: 100 %

Insgesamt lagen der Arbeitsgruppe im betrachteten Recherche-Zeitraum nur 2 Metaanalysen zur PICO-Frage vor. Grillo-Ardila et al. [415] aggregierten 5 RCTs mit 442 Patienten mit Sepsis oder septischem Schock. Der Effekt von früher enteraler Ernährung (definiert als Beginn innerhalb der ersten 48 h) verglichen mit keiner oder verzögerter enteralen Ernährung war unsicher und von sehr niedriger Evidenzqualität. Eine frühe enterale Ernährung war lediglich mit einem Trend zu geringerer Beatmungsdauer, bei gleichzeitig höherer Rate an gastrointestinalen Komplikationen (Diarrhö). Keiner der untersuchten Endpunkte wie Notwendigkeit einer Nierenersatztherapie im Krankenhaus oder Mortalität zeigten signifikante Unterschiede.

In der Metaanalyse von Moon et al. [416] wurden 11 Studien (3 RCTs, 3 Kohorten und 5 Fall-Kontroll-Studien) mit 3820 Patienten zum Einfluss einer frühen vs. verzögerten enteralen Ernährung bei Sepsis untersucht. Dabei hatte der Beginn einer enteralen Ernährung innerhalb von 24–96 h nach Aufnahme auf die Intensivstation (mit oder ohne begleitende parenterale Ernährung) keine Vorteile im Hinblick auf die definierten klinischen Endpunkte (Kurz- und Langzeit-Mortalität, Verweildauer und beatmungsfreie Tage). In einer der 11 inkludierten randomisierten kontrollierten Studien [417] mit der höchsten Fallzahl von 2410 Patienten (58 % mit Sepsis als Ursache für den Schock, definiert als Noradrenalindosis > 0,5 µg/kg/min und/oder Laktatkonzentration > 3,8 mmol/l), zeigte sich bei früher enteraler verglichen mit parenteraler Ernährung und vergleichbarer Kalorien- und Proteinzufuhr eine signifikant höhere Rate an Erbrechen, Diarrhö und intestinaler Ischämie. Allerdings blieb die gepoolte Risikobewertung für gastrointestinale Nebenwirkungen inkonklusiv [416].

Die einzige aktuell gültige Leitlinie zum Thema Ernährungstherapie in der Intensivmedizin liegt von der „American Society of Parenteral and Enteral Nutrition (ASPEN)“ vor [418]. Die Zielpopulation dieser Leitlinie ist definiert als kritisch kranke Patienten, die in chirurgischen oder internistischen Intensivstationen behandelt werden und bei denen eine orale Ernährung nicht möglich bzw. unzureichend ist. Die ASPEN-Leitlinie geht somit nicht speziell auf Patienten mit Sepsis oder septischem Schock ein. Die ASPEN-Leitlinienautoren fassen zusammen, dass eine enterale einer parenteralen Ernährung innerhalb der ersten Woche nach Beginn der kritischen Erkrankung bei vergleichbarer Makronährstoffzufuhr nicht überlegen ist und keine Unterschiede in Bezug auf adverse Effekte festgestellt wurden. Die ASPEN empfiehlt daher, dass sowohl der enterale als auch parenterale Zufuhrweg akzeptabel sind, sofern keine allgemeinen Kontraindikationen für den enteralen Zugangsweg vorliegen wie unkontrollierte gastrointestinale Blutung oder mechanischer Ileus [418]. Wesentlich für die Wahl zwischen früher enteraler oder parenteraler Ernährung ist hier aus Sicht der Leitliniengruppe auch die hämodynamische Stabilisierung (s. Abschn. 4.1 und 2.2) und die daraus resultierende metabolische und gastrointestinale Toleranz. Die metabolische Toleranz wird dabei insbesondere über die Glukosekonzentration und den resultierenden Insulinbedarf definiert [419] (s. auch Empfehlung 8.13), die gastrointestinale Toleranz u. a. anhand von klinisch-abdomineller und/oder bildgebender Diagnostik [420]. Anhand des Monitorings der metabolischen und gastrointestinalen Toleranz wird die enterale und/oder parenterale Kalorien- und Proteinzufuhr im weiteren Verlauf gesteuert.

## 8. Weitere Maßnahmen

### 8.1 Kortikosteroide

#### 8.1/Empfehlung/Modifiziert (Stand 2025)

- Empfehlungsgrad: Schwach
- Wir schlagen vor, Hydrocortison mit oder ohne Fludrocortison bei Patienten mit septischem Schock mit anhaltendem Vasopressorbedarf trotz ausreichender Volumengabe einzusetzen.
- Bemerkungen:
- Die empfohlene Dosierung für Hydrocortison beträgt 200 mg/Tag i.v., für Fludrocortison 1 × 50 µg/Tag enteral.
- Als „anhaltender Vasopressorbedarf“ wird die Gabe von Noradrenalin ≥ 0,25 µg ∙ kg−1 min−1 über mindestens vier Stunden bezeichnet.
- SSC-Leitlinienadaptation/Aktualisierung der Evidenz und Änderung des Empfehlungstextes
- Qualität der Evidenz:
- Kurzzeit-Mortalität (28–30 Tage): Moderat ⊕⊕⊕⊝
- Langzeit-Mortalität (90–180 Tage): Niedrig ⊕⊕⊝⊝
- Literatur: [421]
- Konsensstärke: 100 %

Die SSC-Leitlinie 2021 [2] schlägt die Verwendung von Hydrocortison vor, falls sich die Hämodynamik nicht innerhalb von 4 h durch Noradrenalin in einer Dosis von > 0,25 µg kg^−1^min^−1^ stabilisieren lässt. Der Einsatz von Fludrocortison ist in der SSC-Leitlinie bisher nicht berücksichtigt. Auch die S3-Sepsis-Leitlinie 2018 [1] schlug bisher eine Gabe von Hydrocortison für den Fall vor, dass eine adäquate Flüssigkeits- und hochdosierte Vasopressor-Therapie nicht in der Lage sind, eine hämodynamische Stabilität wiederherzustellen. Das „2024 Focused Update: Guidelines on Use of Corticosteroids in Sepsis (…)“ [422] schlägt ebenfalls die Gabe von Kortikosteroiden bei erwachsenen Patienten mit septischem Schock vor. Die Dosierungsempfehlungen sind hier Hydrocortison 200 mg i.v. pro Tag (kontinuierlich oder alle 6 h) mit oder ohne eine Kombination mit Fludrocortison (50 µg enteral täglich) für 7 Tage bzw. bis zu Entlassung von der Intensivstation [422].

Die Qualität der Evidenz, insbesondere was die Kombination mit Fludrocortison betrifft, ist jedoch mäßig bis gering, sodass weitere randomisierte Studien erforderlich sind.

Aktuell publizierte Metaanalysen legen einen potenziellen Vorteil einer Kombination von Hydrocortison mit Fludrocortison nahe. Kürzlich wurde eine hochwertige, auf den Daten von 8528 individuellen Patienten beruhende Metanalyse (Individual Patient Level-Meta-Analysis – IPMA) aus 24 randomisierten placebokontrollierten Studien publiziert [423]. Diese zeigte auf, dass die Kombination von Fludrocortison mit Hydrocortison im Vergleich zu einer Behandlung mit Hydrocortison alleine mit einer Reduktion der 90-Tage-Mortalität assoziiert ist (RR 0,86; 95 % CI 0,79 bis 0,92 für Hydrocortison plus Fludrocortison und RR 0,96; 95 % CI 0,82 bis 1,12 ohne Fludrocortison) [423]. Allerdings sind bisher nur zwei randomisierte Studien der gleichen französischen Studiengruppe bei Patienten mit sehr hoher 90-Tage-Sterblichkeit in der Placebogruppe (308/627 bzw. 49,1 % und 73/149 bzw. 63 %) publiziert, die dieses nachgewiesen haben [424, 425].

Eine Metaanalyse aus dem Jahr 2024, die auf den gepoolten Studienergebnissen von 9563 Patienten aus 45 randomisierten placebokontrollierten Studien beruhte, kam zu dem Ergebnis, dass Kortikosteroide unabhängig von deren Stoffklasse wahrscheinlich die Kurzzeit-Mortalität reduzieren (RR 0,93; 95 % CI 0,88 bis 0,99). Kortikosteroide haben mit hoher Sicherheit günstige Effekte auf die Verbesserung der Hämodynamik („shock reversal“) innerhalb der ersten 7 Tage (RR 1,24; 95 % CI 1,11 bis 1,38). Die Studie berechnete eine optimale Hydrocortison-Äquivalenz-Dosis, welche bei 260 mg/Tag lag, d. h., bei höheren Dosierungen sind keine verbesserten Effekte zu erwarten [421].

In einer retrospektiven Analyse von administrativen Daten eines US-amerikanischen Krankenhausunternehmens aus den Jahren 2016–2020 war die Krankenhaussterblichkeit von Patienten mit septischem Schock, welche Hydrocortison erhielten, höher als bei Patienten, die mit einer Kombination aus Hydrocortison/Fludrocortison behandelt wurden (43.669/85.995 bzw. 50,8 % vs. 1076/2280 bzw. 47,2 %). Diese Observationsstudie ist limitiert durch den retrospektiven Ansatz und das Fehlen einer Kontrollgruppe [426].

Die in den beiden französischen Studien verwendete Fludrocortison-Dosierung betrug 50 µg täglich über 7 Tage, verabreicht über die Magensonde [424, 425].

In einer aktuellen Arbeit wurden 153 Patienten mit septischem Schock unterschiedliche Tagesdosierungen von Fludrocortison (1 × 50 µg, 2 × 50 µg und 4 × 50 µg) enteral verabreicht. Bei 97 % der Patienten fanden sich nachweisbare Plasma-Konzentrationen von Fludrocortison, die sich zwischen den einzelnen Gruppen nicht signifikant unterschieden [427].

Hinsichtlich der Dauer und des Ausschleichens der Kortikosteroide gibt es unterschiedliche Konzepte. Laut einer Expertenkommission verwendeten die meisten Studien eine Infusionsdauer von 5 bis 7 Tagen [422]. Nicht in allen Studien wurden Ausschleichschemata verwendet. Beispielsweise wurde in der VANISH-Studie [261] Hydrocortison (HC) 4 × 50 mg i.v. für 5 Tage gegeben, dann HC 2 × 50 mg für 3 Tage und HC 1 × 50 mg für 3 weitere Tage [261]. In der HYPRESS-Studie [428] wurde HC 200 mg/Tag i.v. in den ersten fünf Tagen verabreicht, danach HC 100 mg/Tag an den Tagen 6 und 7, HC 50 mg/Tag an den Tage 8 und 9 und schließlich 25 mg/Tag an den Tagen 10 und 11 [428]. Für Fludrocortison finden sich bisher keine Ausschleichschemata in der Literatur. Ein narratives Review rät von einem Ausschleichen („tapering“) von Fludrocortison aus biochemischer Perspektive vorläufig ab [429].

### 8.2 Blutprodukte

#### 8.2.1 Erythrozyten

##### 8.2/Empfehlung/NEU (Stand 2025)

- Empfehlungsgrad: Stark
- Wir empfehlen, bei Patienten mit Sepsis oder septischem Schock die Indikation zur Gabe von Erythrozytenkonzentraten bei einem Hb-Wert von < 4,34 mmol/L (< 7,0 g/dl) oder beim Vorliegen physiologischer Transfusionskriterien zu stellen.
- SSC-Leitlinienadaptation/Änderung des Empfehlungstextes
- Qualität der Evidenz:
- Mortalität (30 Tage): Moderat ⊕⊕⊕⊝
- Mortalität (90 Tage): Niedrig ⊕⊕⊝⊝
- Literatur: [430]
- Konsensstärke: 100 %

Grundlage für diese Empfehlung ist eine Metaanalyse [430] der drei RCT-Studien [431–433]. Die Metaanalyse zeigt keinen Unterschied in der 28-Tage-Sterblichkeit (OR 0,99; 95 % CI 0,67 bis 1,46, moderate Qualität der Evidenz) in den beiden Vergleichsgruppen zwischen einer liberalen (Hämoglobinschwelle < 9 g/dl) oder restriktiven Strategie (Hämoglobinschwelle < 7 g/dl) der Erythrozytentransfusion [2]. Seit der S3-Sepsis-Leitlinie 2018 [1] und der SSC-Leitlinie 2021 [2] wurden zwei relevante neue Studien identifiziert.

In einer prospektiven, nichtrandomisierten Studie von Mustahsin et al. [434] wurden die Ergebnisse des systematischen Reviews mit Metanalyse von Hirano et al. [430] bezüglich der 28-Tage-Mortalität bestätigt. Allerdings zeigte sich eine bessere 90-Tage-Überlebensrate, wenn die Patienten oberhalb des Transfusions-Triggers (6,9 g/dl) transfundiert wurden. Nachteil dieser prospektiven Studie ist eine fehlende Randomisierung, die geringe PatientInnen-Zahl und der Einschluss der PatientInnen bei schon erniedrigtem Hb-Wert (Median 9,5 g/dl) [434].

In einer weiteren retrospektiv angelegten Studie wurden PatientInnen mit einer Sepsis untersucht, die ≥ 65 Jahre waren. Grundlage waren die Daten des MIMIC-IV-Datensets. Insgesamt wiesen 5390 Patienten einen Hb-Wert zwischen 7–11 g/dl auf. Davon wurden 1206 Patienten transfundiert und 4184 Patienten nicht. Die Sterblichkeitsrate der Patienten in der transfundierten Gruppe war niedriger als die der nicht transfundierten Gruppe (10,61 % gegenüber 16,92 %, *p* < 0,001). Die 28-Tage-Sterblichkeitsrate der Patienten in der transfundierten Gruppe mit 7–8 g/dl (10,81 % vs. 16,69 %, *p* = 0,004) und 8–9 g/dl (9,34 % vs. 19,32 %, *p* < 0,001) war signifikant niedriger als in der nicht transfundierten Gruppe. Gleichzeitig kann eine Bluttransfusion das Sterberisiko erhöhen, wenn der Hb-Wert über 10 g/dl liegt [435]. Das retrospektive Studiendesign und die fehlende Unterscheidung einer chronischen Anämie von einer akuten Anämie limitieren eine Bewertung in diesem speziellen Kollektiv von Patienten ≥ 65 Jahre. Zusammenfassend ist die Qualität der Evidenz zu der Fragestellung einer restriktiven versus einer liberalen Transfusionsstrategie bei Patienten mit Sepsis niedrig, eine restriktive Transfusionsstrategie ist jedoch ressourcenschonend und kosteneffektiv.

#### 8.2.2 Erythropoetin

Es gibt bisher keine Studien zur Erythropoetin-Nutzung speziell bei septischen Patienten. Eine Metaanalyse von randomisierten Studien bei kritisch erkrankten Patienten untersuchte als primärem Outcome die Sterblichkeit, unabhängig von den primären Endpunkten der einbezogenen Studien. Zu den sekundären Endpunkten gehörten die Dauer des Krankenhausaufenthalts sowie der Aufenthaltsdauer auf der Intensivstation, die Dauer der mechanischen Beatmung und unerwünschte Ereignisse im Zusammenhang mit der Verwendung von Erythropoetin-Rezeptor-Agonisten, wie arterielle und venöse Thrombosen sowie Bluthochdruck. Zusätzlich wurden transfusionsbezogene Ergebnisse, einschließlich der Transfusionsunabhängigkeit – also der Fähigkeit der Erythropoetin-Rezeptor-Agonisten, die Notwendigkeit für Bluttransfusionen zu reduzieren –, die Anzahl der pro Patient transfundierten Bluteinheiten und Ereignisse, die im Zusammenhang mit Transfusionen auftraten, erfasst [436]. Die Auswertungen zeigen zwar einen geringen Rückgang des Bedarfs an Erythrozyten-Transfusionen, jedoch ohne Einfluss auf die Mortalität. Ein Vorteil bei Sepsis und septischem Schock ist daher unwahrscheinlich. Erythropoetin könnte zudem das Risiko für thrombotische Ereignisse erhöhen [437–440].

#### 8.2.3 Plasmapräparate

##### 8.3/Empfehlung/Modifiziert (Stand 2025)

- EK
- Wir empfehlen, bei Patienten mit Sepsis oder septischem Schock therapeutisches Plasma NICHT zur Behandlung eines Volumenmangels, sondern ausschließlich entsprechend den Indikationen der „Querschnitts-Leitlinien zur Therapie mit Blutkomponenten und Plasmaderivaten“ der BÄK [441] einzusetzen.
- Zusätzliche DSG-Leitlinienempfehlung
- Konsensstärke: 100 %

In Deutschland sind vier Arten therapeutischen Plasmas verfügbar: gefrorenes Frischplasma (GFP), Solvent-Detergent-behandeltes Plasma (SDP), Methylenblau-Licht-behandeltes Plasma (MLP) und lyophilisiertes Humanplasma (LHP). Es gibt keine randomisierten Studien zu verschiedenen Plasmasubstitutionsregimen oder Präparaten bei kritisch kranken Patienten. Empfehlungen basieren auf Expertenmeinungen und besagen, dass Plasma bei dokumentierter Gerinnungsstörung (Quick < 50 %, APTT > 45 s und/oder Fibrinogen < 1 g/l) sowie aktiver Blutung oder geplanten Eingriffen gegeben werden kann. 1 ml Plasma/kg Körpergewicht erhöht dabei die Spiegel der Gerinnungsfaktoren und Inhibitoren oder den Quickwert um 1 IE/dl bzw. um 1 % bei fehlender Umsatzsteigerung bzw. um 0,5 bis 1,0 IE/dl oder um 0,5 bis 1,0 % bei Umsatzsteigerung (Fibrinogenspiegel: um 0,02 bis 0,03 g/l bzw. 2 bis 3 mg/dl) [441].

#### 8.2.4 Thrombozyten

##### 8.4/Empfehlung/Modifiziert (Stand 2025)

- EK
- Wir schlagen vor, bei Patienten mit Sepsis oder septischem Schock KEINE Thrombozytentransfusion durchzuführen, es sei denn, es liegt eine relevante Blutung oder eine schwere Thrombozytopenie vor (< 10.000/µl). Vor operativen Eingriffen, zentralvenösen Punktionen bzw. Zugängen oder Liquorpunktion soll die Gabe nach Vorgaben der „Querschnitts-Leitlinien zur Therapie mit Blutkomponenten und Plasmaderivaten“ der BÄK [441] erfolgen.
- Zusätzliche DSG-Leitlinienempfehlung
- Konsensstärke: 100 %

Es gibt keine RCTs zur prophylaktischen Thrombozytentransfusion bei septischen oder kritisch erkrankten Patienten. Eine neuere Propensity-Score-Matching-Analyse von Wu et al. [442] zeigte, dass die Thrombozytentransfusion bei septischen Patienten mit schwerer Thrombozytopenie (Thrombozytenzahl ≤ 50/nl) mit einer erhöhten Sterblichkeit im Krankenhaus verbunden war (OR-Bereich 1,340 bis 1,525; *p* < 0,05). Sie war jedoch nicht mit der 90-Tage-Mortalität oder der Dauer des Aufenthalts auf der Intensivstation verbunden [442]. Eine sehr ähnliche Arbeit von He et al. [443] der gleichen Datenbasis (MIMIC) untersuchte eine Thrombozytentransfusion bei einer Schwelle von < 150.000/µl. In dieser Studie wurde die Thrombozytentransfusion mit einer höheren 28- (36,9 % vs. 30,4 %; OR 1,21; 95 % CI 1,01 bis 1,46; *p* = 0,039) und 90-Tage-Gesamtmortalität (50,8 % vs. 44,6 %; OR 1,13; 95 % CI 1,00 bis 1,31; *p* = 0,048) in Verbindung gebracht [443]. Diese Ergebnisse weisen auf die potenziellen Gefahren der Thrombozytentransfusion bei Intensivpatienten mit Sepsis und Thrombozytopenie hin. Für die Gabe von Thrombozyten bei den in der Empfehlung erwähnten Indikationen wird auf das Abschn. 2.5.2 der Querschnitts-Leitlinien der BÄK [441] verwiesen.

### 8.3 Immunglobuline

#### 8.5/Empfehlung/Bestätigt (Stand 2025)

- Empfehlungsgrad: Schwach
- Wir schlagen vor, bei Patienten mit Sepsis oder septischem Schock intravenöse Immunglobuline NICHT anzuwenden.
- SSC-Leitlinienadaptation/Aktualisierung der Evidenz
- Qualität der Evidenz:
- Mortalität: Sehr niedrig ⊕⊝⊝⊝
- Literatur: [444]
- Konsensstärke: 100 %

Nach der SSC-Leitlinie 2021 [2] und der S3-Leitlinie der DSG konnten in der Literatursuche nur zwei relevante Publikationen neu in die Bewertung mit aufgenommen werden.

In der Arbeit von Perrella et al. [445] wurde der Einsatz von Pentaglobin bei septischen Patienten nach einem großen abdominellen chirurgischen Eingriff untersucht. Die Auswertung der Studie zeigte, dass die Verwendung von pentamerem IgM keine klinischen Vorteile bei der Vorbeugung von Sepsis nach größeren abdominalen Operationen zu bieten scheint [445]. In einer neuen Metaanalyse [444] von 31 RCT konnte aufgezeigt werden, dass der Einsatz von IVIG die Sepsis-Sterblichkeit verringert [445]. IgM-angereichertes IVIG ist sowohl bei Sepsis bei Erwachsenen als auch bei Neugeborenen wirksam, während Standard-IVIG nur bei Sepsis bei Erwachsenen wirksam ist. In einer Netzwerk-Metaanalyse von Yang et al. [446] konnte ebenfalls aufgezeigt werden, dass eine IVIG-Behandlung wahrscheinlich die Gesamtmortalität von Patienten mit Sepsis verringern kann. Aufgrund dieser neuen Metaanalysen wurde eine neue Analyse der Evidenz und GRADE-Bewertung für den Level of Evidence für Immunoglobuline verglichen mit Standardtherapie bei Patienten mit Sepsis oder septischem Schock durchgeführt. Es zeigte sich hier eine nur schwache Evidenz aufgrund von Risiko für Bias, Indirektheit (unterschiedliche Vergleichstherapien) und hohem Verdacht auf Publikationsbias. Zusammenfassend ist die Qualität der Evidenz basierend auf den vorhandenen Daten sehr niedrig. Es wird eine schwache Empfehlung gegen den Einsatz von Immunglobulinen ausgesprochen.

### 8.4 Blutreinigung

#### 8.4.1 Unselektive Adsorptionsverfahren

##### 8.6/Empfehlung/Modifiziert (Stand 2025)

- Empfehlungsgrad: Stark
- Wir empfehlen, dass bei Patienten mit Sepsis oder septischem Schock unselektive Adsorptionsverfahren NICHT eingesetzt werden.
- Zusätzliche DSG-Leitlinienempfehlung/Aktualisierung der Evidenz
- Qualität der Evidenz:
- Mortalität oXiris®: Sehr niedrig ⊕⊝⊝⊝
- Mortalität CytoSorb®: Moderat ⊕⊕⊕⊝
- Mortalität CPFA: Sehr niedrig ⊕⊝⊝⊝
- Mortalität HA 330: Sehr niedrig ⊕⊝⊝⊝
- Literatur: [447]
- Konsensstärke: 100 %

Mit unselektiven Adsorptionsverfahren soll im Rahmen der Sepsis die Entfernung von Inflammationsmediatoren wie beispielsweise Zytokinen aus dem Blut mittels Adsorption gelingen [448]. Diese Verfahren umfassen eine heterogene Gruppe, die sowohl reine Adsorptionsverfahren aus dem Vollblut (z. B. CytoSorb®) als auch kombinierte Verfahren aus Nierenersatz und Adsorption des zuvor separierten Plasmas einschließen (z. B. CPFA, „coupled plasma filtration adsorption“) [449]. Für viele zugelassene Adsorptionsverfahren sind keine oder nur wenige randomisierte Studien verfügbar, sodass deren Nutzen im Rahmen der Sepsis nicht sicher bewertet werden kann und die Durchführung weiterer klinischer Studien sinnvoll erscheint.

Die randomisierte, multizentrische COMPACT-2-Studie zum Einsatz einer hochdosierten kombinierten Plasmafiltration und Adsorption (CPFA) wurde im Rahmen einer Zwischenauswertung bei potenziellen negativen Auswirkungen gestoppt. Insbesondere in der Frühphase des septischen Schocks zeigte sich bei Patienten, die nicht dialysepflichtig waren, eine dosisabhängige Übersterblichkeit [450]. Eine Metaanalyse von Chen et al. aus dem Jahre 2023 [447] zeigte keinen signifikant günstigen Effekt von CPFA auf die Mortalität auf (RR 1,20; 95 % CI 0,82 bis 1,76) [447].

Zum Einsatz der Hämoperfusion mit dem unselektiven Adsorber CytoSorb® wurden seit der letzten Leitlinie mehrere Metaanalysen veröffentlicht, wobei in keiner ein günstiger Effekt hinsichtlich der Mortalität berichtet wird. In der Metaanalyse von Chen et al. fand sich ein tendenziell ungünstiger Effekt auf die Mortalität (RR 1,39; 95 % CI 0,97 bis 1,98) [447]. Eine aktuell in Deutschland rekrutierende multizentrische randomisierte kontrollierte Studie zum Einsatz von CytoSorb® im septischen Schock (PROCYSS, www.clinicaltrials.gov/study/NCT04963920) wird wahrscheinlich weitere Erkenntnisse zeigen.

HA330 ist ebenfalls ein unselektiver Hämoperfusionsadsorber, welcher in den letzten Jahren vermehrt im deutschsprachigen Raum zur Blutreinigung in der Sepsis eingesetzt wird. Auch hierfür zeigte sich in der Metaanalyse von Chen et al. [447] kein günstiger Effekt auf die Sterblichkeit septischer Patienten (RR 0,61; 95 % CI 0,35 bis 1,09) [447].

Die Anzahl der durchgeführten Studien und Anwendungsbeobachtungen für den ebenfalls unselektiven Adsorber oXiris® (eine Dialysemembran mit zusätzlichen Adsorber-Eigenschaften) ist deutlich geringer als z. B. für CytoSorb®. Die Metaanalyse von Chen et al. [447] zeigte aber ebenso bei diesem Verfahren keinen Überlebensvorteil (RR 0,72; 95 % CI 0,29 bis 1,78) [447].

#### 8.4.2 Polymyxin-B-Hämoperfusion

##### 8.7/Empfehlung/NEU (Stand 2025)

- EK
- Wir können bei Patienten mit Sepsis oder septischem Schock weder für noch gegen den Einsatz einer Polymyxin-B-Hämoperfusion eine Empfehlung aussprechen.
- Zusätzliche DSG-Leitlinienempfehlung
- Konsensstärke: 100 %

Ziel des Verfahrens ist die Entfernung von Endotoxinen im Rahmen der gramnegativen Sepsis durch die Anwendung von Polymyxin-B-immobilisierten auf Polystyren basierten Fasern. Die Leitlinie der SSC riet im Jahr 2021 von der Anwendung einer Polymyxin-B-Hämoperfusion ab. Dies wird durch eine Metaanalyse aus dem Jahr 2013 begründet, die auf den ersten Blick einen Überlebensvorteil durch die Anwendung zeigt, der sich aber durch die Betrachtung der randomisierten Studien nicht bestätigen ließ [451]. Zudem ist das Verfahren sehr kostspielig.

Die größte im Jahr 2018 veröffentlichte multizentrisch randomisierte Studie, die EUPHRATES-Studie [452], konnte keinen signifikanten Unterschied im Überleben der Patienten mit und ohne die Anwendung des Verfahrens zeigen [452], sodass sich die vorherige S3-Sepsis-Leitlinie der DSG nur für die Anwendung in klinischen Studien aussprach.

In den letzten Jahren wurden einige inkonklusive Metaanalysen veröffentlicht. Putzu et al. [453] inkludierten 37 randomisierte Studien und stellten keinen signifikanten Vorteil auf das Überleben fest, wenn nur die Studien mit niedrigen Bias-Risiko betrachtet wurden. Studien nach 2010 zeigten zudem eine signifikant höhere Sterblichkeit durch die Anwendung des Verfahrens. Positive Effekte wurden zudem nur in Studien in Asien und im nichteuropäischen bzw. US-amerikanischen Raum beobachtet [453]. Chen et al. [447] verglichen 60 randomisierte Studien, bei denen insgesamt 16 Verfahren zur Blutreinigung im Rahmen einer Sepsis Anwendung fanden. Bei der Betrachtung der Polymyxin-B-Hämoperfusion zeigt sich eine signifikant geringe Sterblichkeit durch die Anwendung [447]. Die Daten sind allerdings heterogen und mit einem Publikationsbias assoziiert. In einer weiteren aktuellen Metaanalyse wird ebenso von einem Überlebensvorteil durch die Anwendung berichtet [454]. Der Effekt war hier besonders deutlich bei Patienten unter 70 Jahren und bei mehreren Infektionsorten. Limitierend ist, dass viele Beobachtungsstudien in die Auswertung inkludiert wurden.

Eine Post-hoc-Analyse der EUPHRATES-Studie zeigte einen Überlebensvorteil bei Patienten mit moderat erhöhter Endotoxinaktivität (EAA) von 0,6–0,89 [455]. Der fehlende Effekt in der Gesamtpopulation könnte auf einen Sättigungseffekt des Adsorbers in der Gruppe mit sehr hoher EAA (> 0,9) oder auf einen überschrittenen „point of no return“ hindeuten. Aktuell wird in einer multizentrischen randomisierten Folgestudie (TIGRIS, https://clinicaltrials.gov/study/NCT03901807) in den USA genau diese Subgruppe (EAA 0,6–0,89) rekrutiert, sodass zukünftig neue Daten zu erwarten sind.

#### 8.4.3 Therapeutischer Plasmaaustausch/Plasmapherese

##### 8.8/Empfehlung/NEU (Stand 2025)

- EK
- Wir können bei Patienten mit Sepsis oder septischem Schock weder für noch gegen den Einsatz einer Plasmapherese eine Empfehlung aussprechen.
- Zusätzliche DSG-Leitlinienempfehlung
- Konsensstärke: 100 %

Weder die deutsche S3-Sepsis-Leitlinie der DSG noch die 2021er-Leitlinie der SSC nahmen zur Anwendung der Plasmapherese bei Patienten mit Sepsis oder septischem Schock bisher Stellung, da die verfügbaren Daten bis dato nicht ausreichend waren. Ziel der Plasmapherese im Rahmen der Sepsis ist die Entfernung des Plasmas mit den darin enthaltenden Mediatoren der Wirtsreaktion und der Ersatz durch Plasma von gesunden Spendern, welches verbrauchte protektive Plasmaproteine enthält [456].

Die Aufnahme dieses Verfahrens in die Leitlinie beruht auf einer deutlichen Zunahme an Studien seit der letzten Leitlinienversion. Eine Metaanalyse von Chen et al. aus dem Jahre 2023 [447] zeigt, dass der Einsatz der Plasmapherese mit einem signifikanten Überlebensvorteil assoziiert ist [447]. Eine weitere Metaanalyse inkludierte sowohl Beobachtungsstudien, randomisierte Studien als auch andere Metaanalysen, die zwischen 2019 und 2023 publiziert wurden. Auch hier zeigt sich eine signifikant geringere Sterblichkeit bei der Anwendung der Plasmapherese bei Patienten mit septischem Schock [457]. Diese Ergebnisse sind aber bei geringen Fallzahlen und hoher Heterogenität kritisch zu bewerten.

Auch in der 2024 veröffentlichten Metaanalyse von Kuklin et al. [458] wurde ein signifikanter Überlebensvorteil nachgewiesen. Allerdings wurden Studien in einem Zeitraum zwischen 1966 und 2022 inkludiert, die sich in Design und Patientenkollektiv erheblich unterscheiden (randomisiert, observierend, gematchte Population, auch COVID-19-Patienten). Werden nur die randomisierten Studien betrachtet, dann bleibt der Überlebensvorteil zwar bestehen, das betrachtete Kollektiv wird mit 331 Patienten aber sehr klein (RR 0,62; 95 % CI 0,46 bis 0,83). Eine weitere Metaanalyse inkludierte sowohl pädiatrische als auch erwachsene Patienten mit Sepsis oder septischem Schock. Bei der Betrachtung der Erwachsenen zeigt sich, dass die Plasmapherese mit einem signifikanten Überlebensvorteil assoziiert ist, aber nur dann, wenn gefrorenes Frischplasma anstelle von Frischplasma und Humanalbumin (HA) oder nur HA verwendet wird [459]. Dies deutet eher auf einen therapeutischen Effekt des additiven Ersatzes verbrauchter protektiver Proteine hin. In der Summe deutet die Studienlage auf einen möglichen Nutzen der Plasmapherese hin, die Datenqualität der Originalarbeiten ist aber zum Aussprechen einer Empfehlung für den routinemäßigen Einsatz noch nicht ausreichend.

Die randomisierte kontrollierte EXCHANGE-Pilot-Studie aus Deutschland zeigte an einem kleinen Kollektiv einen signifikanten Effekt auf den primären Endpunkt, d. h. Hämodynamik beim Einsatz der Plasmapherese [460]. Diese Studie demonstrierte die Machbarkeit einer großen multizentrischen randomisierten kontrollierten Studie (EXCHANGE‑2, https://clinicaltrials.gov/study/NCT05726825), welche aktuell in Deutschland Patienten rekrutiert. Die Ergebnisse werden in den kommenden Jahren erwartet.

### 8.5 Antikoagulation und Thromboseprophylaxe

#### 8.9/Empfehlung/Modifiziert (Stand 2025)

- Empfehlungsgrad: Stark
- Wir empfehlen bei Patienten mit Sepsis oder septischem Schock eine pharmakologische Prophylaxe einer venösen Thromboembolie (VTE) mittels niedermolekularen Heparins (NMH).
- SSC-Leitlinienadaptation/Aktualisierung der Evidenz und Änderung des Empfehlungstextes
- Qualität der Evidenz:
- Tiefe BVT: Moderat ⊕⊕⊕⊝
- LAE: Niedrig ⊕⊕⊝⊝
- Literatur: [461]
- Konsensstärke: 100 %

#### 8.10/Empfehlung/NEU (Stand 2025)

- EK
- Wir schlagen vor, bei hochgradig eingeschränkter Nierenfunktion und/oder septischem Schock mit hohem Vasopressorbedarf entweder niedermolekulares Heparin (NMH) unter engmaschigen Anti-Xa-Kontrollen oder unfraktioniertes Heparin (UFH) einzusetzen.
- Zusätzliche DSG-Leitlinienempfehlung
- Konsensstärke: 100 %

Die Inzidenz venöser Thromboembolien (VTE) bei Patienten auf Intensivstationen (ITS) variiert je nach Studie und verwendetem Diagnosekriterium (klinischer Verdacht, Ultraschallbefunde, Schnittbildgebung etc.) erheblich. Unabhängig von diesen Unschärfen muss mit einer deutlich erhöhten Inzidenz von VTE bei ITS-Patienten gerechnet und dieser dementsprechend auch therapeutisch Rechnung getragen werden. Die erhöhte Rate an VTE wird auf verschiedene Faktoren, wie z. B. Sedierung, Immobilisation, invasive Katheter und eine oftmals stark ausgeprägte Gerinnungsaktivierung im Rahmen der Grunderkrankung (Sepsis, Malignome, etc.), zurückgeführt [461–463]. Es können grundsätzlich verschiedene Antikoagulanzien zur Thromboseprophylaxe bei Patienten auf der Intensivstation eingesetzt werden, wobei niedermolekulares (NMH) und unfraktioniertes Heparin (UFH) die häufigsten Vertreter darstellen. Eine aktuelle Metaanalyse berichtete über eine geringere Inzidenz von tiefen Venenthrombosen (TVT) bei Patienten, die NMH (*n* = 3293) erhielten, im Vergleich zu jenen, die UFH (*n* = 2834) erhielten (OR 0,72; 95 % CI 0,46 bis 0,98). Dieses ist möglicherweise auf eine bessere Bioverfügbarkeit und eine längere Plasmahalbwertszeit zurückzuführen, bei einer ähnlich hohen Inzidenz schwerer Blutungen (OR 1,71, 95 % CI 0,49 bis 5,95) [461]. Der Einsatz von NMH bei eingeschränkter Nierenfunktion sollte immer engmaschig evaluiert werden, insbesondere bei einer Kreatinin-Clearance (CrCl) < 30 ml/min, da es hier vermehrt zu Akkumulationen kommen kann. Das Therapiemonitoring sollte in diesen Fällen mittels Anti-Xa-Spiegelbestimmungen erfolgen, um überschießende Wirkspiegel frühzeitig zu detektieren. Es existieren zumindest aus einzelnen Kliniken Daten [464], dass NMH auch bei Patienten mit hochgradig eingeschränkter Nierenfunktion sicher eingesetzt werden können. Alternativ ist hier aber auch der Einsatz von UFH möglich.

Bzgl. der anzustrebenden Dosierung schlussfolgerten Eck et al. [465] in einer 2022 erschienenen systematischen Übersichtsarbeit und Metaanalyse, dass eine „intermediate dose“ von NMH (z. B. Enoxaparin 40–60 mg/24 h) (*n* = 22.482) möglicherweise einer niedrigen Dosis (z. B. Enoxaparin 20 mg/24 h) (*n* = 5612) vorzuziehen ist, da sie ein besseres Gleichgewicht zwischen Nutzen und Schaden für die Prävention von VTE bietet, wenngleich mit niedriger bis mittlerer Evidenzqualität [465]. Konträr zu dem 2024 publizierten „First Update der European Guidelines on Perioperative Venous Thromboembolism Prophylaxis“ [466], welches sich dieser Empfehlung anschloss, geben die Autoren der S3-Leitlinie hier bewusst keine Empfehlung ab. Grund dafür ist, dass die in der Metaanalyse inkludierten Primärdaten zeigen, dass bei den meisten Patienten in der „intermediate group“ vornehmlich Dosierungen an NMH genutzt wurden, welche in Deutschland, Österreich und der Schweiz bei Intensiv-Patienten ohnehin als „Low-Dose-Dosierung“ gelten (z. B. Enoxaparin 40 mg/24 h oder Tinzaparin 4500 IE/24 h) und diese mit noch niedrigeren Dosen verglichen wurden. Daher könnte man die Ergebnisse eher als eine Bestätigung des bereits etablierten „good clinical practice“ ansehen, wobei nicht auszuschließen ist, ob manche Patientengruppen nicht auch von noch höheren Dosierungen profitieren könnten. Hierfür liegen aktuell aber keine Daten vor.

#### 8.11/Empfehlung/NEU (Stand 2025)

- Empfehlungsgrad: Schwach
- Wir schlagen vor, bei Patienten mit Sepsis oder septischem Schock eine intermittierende pneumatische Kompression als VTE-Prophylaxe einzusetzen, wenn eine pharmakologische VTE-Prophylaxe kontraindiziert ist.
- Zusätzliche DSG-Leitlinienempfehlung
- Qualität der Evidenz:
- Inzidenz venöser Thromboembolien: Sehr niedrig ⊕⊕⊝⊝
- Inzidenz tiefer Venenthrombosen: Niedrig ⊕⊕⊝⊝
- Inzidenz von Lungenembolien: Niedrig ⊕⊕⊝⊝
- Literatur: [467]
- Konsensstärke: 100 %

Drei kürzlich veröffentlichte systematische Übersichtsarbeiten mit Metaanalysen [461, 467, 468] untersuchten die Wirksamkeit der mechanischen Thromboseprophylaxe bei kritisch kranken Patienten. In einer systematischen Übersichtsarbeit und Metaanalyse, die 10 Studien (6 randomisierte klinische Studien [RCT] und 4 Beobachtungsstudien) mit 4759 kritisch kranken Patienten umfasste, reduzierte eine intermittierende pneumatische Kompression (IPC) die Inzidenz von jeglicher VTE (4 Studien; RR 0,35; 95 % CI 0,18 bis 0,68; *p* = 0,002; I^2^ = 51 %), TVT (3 Studien; RR 0,34; 95 % CI 0,19 bis 0,60; *p* = 0,002; I^2^ = 0 %) und Lungenarterienembolien (LAE) (2 Studien; RR 0,17; 95 % CI 0,06 bis 0,50; *p* = 0,001; I^2^ = 0 %), ohne Unterschied in der Gesamtsterblichkeit (4 Studien; RR 0,95; 95 % CI 0,82 bis 1,09; *p* = 0,46; I^2^ = 0 %), im Vergleich zu keiner Thromboseprophylaxe [467]. Eine zweite Metaanalyse [468] umfasste 5 RCTs mit 3133 internistischen und chirurgischen kritisch kranken Patienten. IPC reduzierte hier die Inzidenz von VTE (OR 0,36; 95 % CI 0,18 bis 0,71) und TVT (OR 0,45; 95 % CI 0,21 bis 0,90) im Vergleich zu keiner Behandlung, ohne Unterschied in der Gesamtsterblichkeit (OR 0,87; 95 % CI 0,49 bis 1,42) [468]. Eine dritte Metaanalyse [461] umfasste 13 RCTs mit gemischten Populationen (9619 kritisch kranke Erwachsene – darunter medizinische, chirurgische und Traumapatienten). Kompressionsgeräte (IPC oder sequenzielle Kompressionsgeräte – SCD) reduzierten im Vergleich zur Kontrollgruppe weder das Risiko für venöse Thromboembolien (VTE) (OR 0,82; 95 % CI 0,27 bis 2,84) noch für TVT (OR 0,85; 95 % CI 0,50 bis 1,50). Nur 5 Studien untersuchten das Auftreten einer LAE bei kritisch kranken Erwachsenen, wobei keine Wirksamkeit von IPC gezeigt werden konnte (OR 0,20; 95 % CI 0,01 bis 2,87) [461]. Auch wenn insgesamt die verfügbaren Daten widersprüchliche Ergebnisse aufweisen, zeigt sich dennoch ein positiver Trend für den Einsatz der IPC bei Patienten ohne pharmakologische VTE-Prophylaxe. Für einen generellen Einsatz von IPC in Kombination mit einer pharmakologischen VTE-Prophylaxe gibt es aus Sicht der Leitlinien-Autoren aktuell keine Datengrundlage. Wobei eine Metaanalyse zumindest einen positiven Trend beim Einsatz einer Kombinationstherapie für weniger TVT, DVT und LAE zeigen konnte [467]. Ob ausgewählte Hochrisikopatienten von einer solchen Kombination profitieren, lässt sich aufgrund der aktuellen Datenlage nicht abschließend beurteilen und muss daher individuell vom jeweiligen Behandlungsteam entschieden werden.

### 8.6 Delirmanagement, Analgesie, Sedierung

#### 8.12 /Empfehlung/NEU (Stand 2025)

- EK
- Wir empfehlen, bei Patienten mit Sepsis oder septischem Schock bezüglich der Analgesie und Sedierung den Empfehlungen der DAS-Leitlinie [362] zu folgen.
- Literatur: [362]
- Konsensstärke: 100 %

Zur Diagnostik und Behandlung von Agitation, Schmerz und Delir sowie zum Einsatz von Sedativa wurde die im Jahre 2020 aktualisierte S3-Leitlinie „Analgesie, Sedierung und Delirmanagement in der Intensivmedizin (DAS-Leitlinie 2020)“ (AWMF Nr. 001/012, Stand vom 31.03.2021, gültig bis 30.03.2026) [362] veröffentlicht. Sie ist erneut unter der Federführung der Deutschen Gesellschaft für Anästhesiologie und Intensivmedizin (DGAI) und der Deutschen Interdisziplinären Vereinigung für Intensiv und Notfallmedizin (DIVI) sowie unter Beteiligung weiterer medizinischer Fachgesellschaften entwickelt und verabschiedet worden. Die Empfehlungen dieser nationalen Leitlinie gelten für Deutschland. Die DAS-Leitlinie 2020 empfiehlt, die Sedierungstiefe und Analgesie patientenorientiert vorzunehmen bzw. anzupassen, mit dem Ziel, eine unnötig tiefe Sedierung zu vermeiden. Ein regelmäßiges Monitoring (in der Regel 8‑stündlich) mit validierten Scoring-Systemen ist hierfür Voraussetzung.

Eine weitere Empfehlung gilt der Steuerung der Sedierungstiefe: Das Sedierungsziel soll für die Patienten klar definiert sein und bedarf einer regelmäßigen Adaptation an die veränderliche klinische Situation. Generell wird empfohlen, bei Fehlen von Kontraindikationen eine möglichst flache Sedierung zu ermöglichen. Dies entspricht einem Zielwert von 0/−1 der Richmond Agitation-Sedation Scale (RASS). Ist eine tiefe Sedierung indiziert, wie z. B. bei therapeutischer Sedierung im Rahmen eines erhöhten intrakraniellen Drucks oder Bauchlagerungstherapie im Rahmen eines ARDS, so wird vorgeschlagen, dass bei sehr tief sedierten Patienten ein EEG-basiertes Messverfahren zum Vermeiden von ‚Burst-Suppression-EEG‘ zum Einsatz kommen [469].

Die US-amerikanische Leitlinie „Prevention and management of pain, agitation/sedation, delirium, immobility and sleep disruption in the ICU“ [470] (PADIS-Guideline) betont ebenfalls die Bedeutung flacher Sedierung, aber auch die klinische Bedeutung der Wahl des Sedativums. So zeigte die MENDS-3-Studie, eine multizentrische Studie mit mehr als 4000 Patienten, bei denen mehr als die Hälfte das Vorliegen einer Sepsis wahrscheinlich war, dass die Verwendung von Dexmedetomidin bei Patienten, die jünger als 65 Jahre waren und nichtchirurgisch behandelt wurden, mit einer erhöhten Mortalität assoziiert sein könnte [471]. Dies führte zu einem Rote-Hand-Brief des Bundesamtes für Arzneimittel und Medizinprodukte (BfArM). Zwei daraufhin durchgeführte Metaanalysen zeigten allerdings, dass die Verwendung von Dexmedetomidin im Vergleich zu allen anderen untersuchten Sedativa und auch Placebo zwar mit Hypotonie und Bradykardie, aber mit einem niedrigeren Delir-Risiko sowie einer kürzeren Beatmungszeit und Intensivaufenthaltsdauer assoziiert war [472] – nicht aber mit einer erhöhten Mortalität [473].

Die PADIS-Guideline [470] betont darüber hinaus die klinische Bedeutung regelmäßiger Spontanatmungsversuche, des Delir-Managements, der Frühmobilisation und Einbeziehung der Familie bzw. Angehöriger [470]. Zusammengefasst zum sog. ABCDEF- bzw. A2F-Bundle („A: assess, prevent and manage pain; B: both spontaneous awakening and breathing trials, C: choice of sedation strategies; D: delirium assessment, prevention and management; E: early mobility and exercise; F: family engagement“) wurde es mittlerweile hinsichtlich der Wirkung auf einzelne Outcomes überprüft. In einer multizentrischen Kohortenstudie mit 68 Zentren und insgesamt mehr als 15.000 Patienten zeigte sich, dass die Adhärenz mit den einzelnen Komponenten des Bündels mit einer niedrigeren 7‑Tage-Mortalität und einer geringeren Wahrscheinlichkeit einer invasiven Beatmung, Koma, Delir, Notwendigkeit von Fixierungen, Wiederaufnahmen auf Intensivstation sowie Verlegung in eine Pflegeeinrichtung assoziiert war [474].

### 8.7 Glukosemanagement

#### 8.13/Empfehlung/Modifiziert (Stand 2025)

- Empfehlungsgrad: Stark
- Wir empfehlen bei Patienten mit Sepsis oder septischem Schock den Beginn einer Insulintherapie bei einem oberen Blutzuckerspiegel von ≥ 180 mg/dl (≥ 10 mmol/l).
- SSC-Leitlinienadoption
- Qualität der Evidenz:
- Hypoglykämie (110–144 mg/dl vgl. 144–180 mg/dl): Moderat ⊕⊕⊕⊝
- Literatur: [475]
- Konsensstärke: 100 %

Im Hinblick auf das Blutzuckermanagement lag der SSC-Empfehlung eine Netzwerk-Metaanalyse [475] von 35 RCTs zugrunde, in der vier verschiedene Blutzuckerziele miteinander verglichen wurden (< 110, 110–144, 144–180 und > 180 mg/dl). Zwischen den vier Blutzuckerbereichen wurde kein signifikanter Unterschied in Bezug auf die Krankenhausmortalität festgestellt. Zielkonzentrationen von < 110 und ein Zielbereich von 110–144 mg/dl waren mit einem erhöhten Hypoglykämierisiko assoziiert, verglichen mit einem Zielbereich von 144–180 mg/dl und > 180 mg/dl. Es wurde kein signifikanter Unterschied im Hypoglykämierisiko zwischen einem Zielwert von 144–180 mg/dl und > 180 mg/dl nachgewiesen (OR 1,72; 95 % CI 0,79 bis 3,7) [475].

Die Society of Critical Care Medicine (SCCM) hat ihre Leitlinie „Guidelines on Glycemic Control for Critically Ill Children and Adults 2024“ [476] aktualisiert. In der SCCM-Leitlinie wird als „Good Practice Statement“ empfohlen, dass protokollbasiert ein Glukosemanagement durchgeführt werden sollte, um eine anhaltende Hyperglykämie ≥ 180 mg/dl (≥ 10 mmol/l) zu therapieren. Die SCCM-Leitlinie berücksichtigt dabei bereits die Daten des bislang größten multizentrischen RCT [477] zum Blutzuckermanagement bei insgesamt 9230 kritisch kranken Patienten (28,4 % mit Sepsis) [477]. Hier war der primäre Endpunkt Intensivliegedauer mit Sterblichkeit als „competing risk“ zwischen einer liberalen (> 215 mg pro Deziliter bzw. > 11,9 mmol pro Liter) und einer strikten Glukosekontrolle (80 bis 110 mg/dl bzw. 4,4–6,1 mmol/l) nicht signifikant unterschiedlich. Alle Patienten erhielten in der ersten Woche des Intensivaufenthaltes eine rein enterale Ernährung und erst ab Beginn der zweiten Woche eine (zusätzliche) parenterale Ernährung. Die Autoren des RCT [477] wiesen darauf hin, dass eine Hyperglykämie nicht alleine durch die metabolischen Veränderungen im Rahmen der kritischen Erkrankung, sondern auch iatrogen durch die Höhe der Makronährstoffzufuhr im Rahmen der Ernährungstherapie verursacht werden kann. In der individuellen Patientendaten-Metaanalyse von Adigbli et al. 2024 [478] wurden aus 38 Studien 20 (*N* = 14.171 Patienten) individuelle Daten zur intensivierten versus konventionellen Glukosekontrolle analysiert. Der Effekt auf die Mortalität war nicht signifikant unterschiedlich, das Risiko einer schweren Hypoglykämie bei intensivierter Glukosekontrolle jedoch signifikant höher (Risikoverhältnis 3,38; 95 % CI 2,99 bis 3,83; *P* < 0,0001) [478].

### 8.8 Stressulkusprophylaxe

#### 8.14/Empfehlung/Modifiziert (Stand 2025)

- Empfehlungsgrad: Stark
- Wir empfehlen, dass Patienten mit Sepsis oder septischem Schock nur bei vorliegenden Risikofaktoren für gastrointestinale (GI-)Blutungen eine Stressulkusprophylaxe erhalten.
- SSC-Leitlinienadaptation/Aktualisierung der Evidenz und Änderung des Empfehlungsgrades
- Qualität der Evidenz:
- Klinisch relevante obere GI-Blutungen: Hoch ⊕⊕⊕⊕
- Literatur: [479]
- Konsensstärke: 100 %

#### 8.15/Empfehlung/Modifiziert (Stand 2025)

- Empfehlungsgrad: Stark
- Wir empfehlen, dass Patienten mit Sepsis oder septischem Schock mit vorliegenden Risikofaktoren für gastrointestinale (GI-)Blutungen eine Stressulkusprophylaxe mit Protonenpumpeninhibitoren erhalten.
- Zusätzliche DSG-Leitlinienempfehlung/Aktualisierung der Evidenz
- Qualität der Evidenz:
- Klinisch relevante obere GI-Blutungen: Moderat ⊕⊕⊕⊝
- Literatur: [480]
- Konsensstärke: 100 %

Patienten mit Sepsis oder septischem Schock weisen häufig einen oder mehrere der 3 unabhängigen Risikofaktoren (respiratorische Insuffizienz, definiert als maschinelle Beatmung > 48 h; Gerinnungsstörung, definiert als INR > 1,5 oder Thrombozytenzahl < 50.000/mm [3]; akutes Nierenversagen) für das Auftreten einer klinisch signifikanten stressbedingten Blutung auf, die mit erhöhter Morbidität und Mortalität assoziiert sein kann. Gleichzeitig kann unter dem Einsatz einer medikamentösen Stressulkusprophylaxe das Risiko einer *Clostridioides-difficile*-Kolitis oder einer Pneumonie erhöht sein [481, 482].

Erstens wurde der Frage nachgegangen, ob eine Stressulkusprophylaxe vs. keine Prophylaxe die klinische Prognose der Zielpopulation beeinflusst, und zweitens, welche Medikation bei Indikation für eine Stressulkusprophylaxe eingesetzt werden sollte. Zu (1) wurde die SSC-Leitlinienempfehlung auf Basis der Cochrane-Metaanalyse von Toews et al. [480] adaptiert. Im Vergleich zu Placebo war in dieser Analyse von 106 Studien mit insgesamt 15.027 Patienten eine Stressulkusprophylaxe vs. keine Prophylaxe/Placebo mit einer signifikanten Reduktion des Blutungsrisikos (RR 0,47; CI 0,39 bis 0,57) assoziiert. Ein signifikanter Einfluss auf die Mortalität wurde nicht gefunden [480].

In dem sog. REVISE-RCT von Cook et al. [483] wurden 4821 Patienten mit mindestens einem der o. g. Risikofaktoren in 68 Intensivstationen randomisiert und der Einsatz von 40 mg Pantoprazol intravenös mit Placebo verglichen. Klinisch bedeutsame obere gastrointestinale Blutungen traten bei 25 von 2385 Patienten (1,0 %) unter Pantoprazol und bei 84 von 2377 Patienten (3,5 %) unter Placebo auf (HR 0,30; 95 % CI 0,19 bis 0,47; *P* < 0,001). Die 90-Tage-Mortalität lag in der Pantoprazol-Gruppe bei 29,1 %, in der Placebo-Gruppe bei 30,9 % (HR 0,94; 95 % CI 0,85 bis 1,04; *P* = 0,25). Alle anderen sekundären Endpunkte inklusive Pneumonierate und Rate der *Clostridioides-difficile*-Infektionen waren in beiden Gruppen nicht signifikant unterschiedlich [483].

Die Empfehlung zur Wahl der Medikation bei Indikation für eine Stressulkusprophylaxe wurde auf Basis der Metaanalyse von Wang et al. [479] gegeben. Zwölf Studien mit 9533 Patienten wurden in die Metaanalyse inkludiert. Protonenpumpeninhibitoren waren, verglichen mit Histamin-H2-Rezeptorantagonisten, mit einer geringeren Inzidenz klinisch bedeutsamer oberer gastrointestinaler Blutungen assoziiert (RR 0,51; 95 % CI 0,34 bis 0,76). Ein signifikanter Einfluss auf die Sterblichkeit wurde nicht gefunden (RR 0,99; 95 % CI 0,93 bis 1,05). Eine Subgruppenanalyse zeigte jedoch, dass Protonenpumpeninhibitoren mit einer niedrigeren 90-Tage-Sterblichkeit bei weniger schwer erkrankten Patienten assoziiert war (RR 0,89; 95 % CI 0,80 bis 0,98). Unterschiede zwischen Protonenpumpeninhibitoren und Histamin-H2-Rezeptorantagonisten in Bezug auf die Pneumonie- und *Clostridioides-difficile*-Infektionsraten zeigten sich nicht [479].

In der zuvor publizierten Metaanalyse von Deliwala et al. [484] wurden insgesamt 14 RCTs mit 28.526 Patienten berücksichtigt. In der gepoolten Analyse waren Protonenpumpeninhibitoren mit einer geringeren Rate an klinisch signifikanten gastrointestinalen Blutungen, verglichen mit Histamin-H2-Rezeptorantagonisten (RR 0,68; 95 % CI 0,57 bis 0,82), assoziiert. Es wurden keine Unterschiede zwischen den Gruppen hinsichtlich der Gesamtmortalität (RR 1,05; 95 % CI 1,00 bis 1,10) oder der Pneumonierate (RR 1,11; 95 % CI 0,82 bis 1,51) gefunden [484].

In dem sog. PEPTIC-RCT [485] von Young und Koautoren wurden 26.828 Patienten hinsichtlich des Effektes einer Stressulkusprophylaxe mit Protonenpumpeninhibitoren versus Histamin-H2-Rezeptorantagonisten analysiert. Klinisch bedeutsame obere gastrointestinale Blutungen traten bei 1,3 % der PPI-Gruppe und 1,8 % der H2-RA-Gruppe auf (RR 0,73; 95 % CI 0,57 bis 0,92). Die Rate an *Clostridioides-difficile-*Infektionen und die Aufenthaltsdauer auf der Intensivstation und im Krankenhaus unterschieden sich zwischen den Behandlungsgruppen nicht signifikant [485].

### 8.9 Vitamine

#### 8.16/Empfehlung/NEU (Stand 2025)

- Empfehlungsgrad: Stark
- Wir empfehlen, bei Patienten mit Sepsis oder septischem Schock hochdosiertes Vitamin C – weder als Monotherapie noch als Kombinationstherapie mit Hydrocortison und hochdosiertem Vitamin B1 (Thiamin) – NICHT zu verwenden.
- SSC-Leitlinienadaptation/Aktualisierung der Evidenz und Änderung des Empfehlungstextes
- Qualität der Evidenz:
- HAT [486]:
- 90 d-Mortalität: Sehr niedrig ⊕⊝⊝⊝
- Monotherapie Vitamin C [487]:
- 30 d-Mortalität: Sehr niedrig ⊕⊝⊝⊝
- Literatur: [486, 487]
- Konsensstärke: 100 %

In der SSC-Leitlinie 2021 wird eine schwache Empfehlung gegen den Einsatz einer hochdosierten intravenösen Vitamin-C-Monotherapie ausgesprochen. In dem Hintergrundtext werden Metaanalysen zugrunde gelegt, die Studien sowohl zur Kombinationstherapie von hochdosiertem Vitamin C mit Hydrocortison und Vitamin B1 (Thiamin) – die sog. HAT-Therapie – als auch Studien, die hochdosiertes Vitamin C als Monotherapie einsetzten, inkludiert.

Im betrachteten Recherchezeitraum lagen der Arbeitsgruppe nun 17 Metaanalysen zur HAT-Therapie und insgesamt 25 Metaanalysen zur hochdosierten Vitamin-C-Monotherapie vor. Die intravenös applizierte Vitamin-C-Dosierung war in den inkludierten Studien unterschiedlich, mit Dosierungsstrategien wie 25–50 mg/kg alle 6 h für 96 h oder 1,5 g alle 6 h über 96 h oder 1 g als Loading-Dosis und weitere 250 mg/h kontinuierlich über 96 h nach Aufnahme auf die Intensivstation. Insbesondere in den Metaanalysen zur HAT-Therapie waren die Arme Intervention und Kontrolle sehr heterogen, u. a. HAT-Therapien bzw. unterschiedliche Vitamin-Kombinationstherapien. Die Kontrollarme erhielten entweder Placebo, eine Hydrocortison-Monotherapie, eine sog. Standardbehandlung oder andere. Zur HAT-Therapie wurde die Netzwerk-Metaanalyse von Fujii et al. 2022 [486] zugrunde gelegt. Insgesamt wurden die Ergebnisse von 43 RCTs mit 10.257 Patienten gepoolt, der primäre Endpunkt war Langzeitmortalität (90 Tage bis 1 Jahr), sekundäre Endpunkte Schweregrad der Organdysfunktion über 72 h, Zeit bis zum Absetzen der Vasopressor-Therapie und die Dauer des Aufenthalts auf der Intensivstation. Es gab keine signifikanten Unterschiede in der Langzeit-Mortalität zwischen der HAT-Therapie und Placebo bzw. Standardtherapie, auch zeigten sich keine Unterschiede zwischen den Therapiearmen (10 RCTs, 7096 Patienten). Auch bei den sekundären Endpunkten zeigten sich keine signifikanten Unterschiede, außer dass die Zugabe von Hydrocortison zu Vitamin C und Vitamin B1 mit einer kürzeren Dauer der Vasopressor-Therapie assoziiert war [486].

Die einzige Metaanalyse, die nur Studien mit einer hochdosierten Vitamin-C-Monotherapie gegen Placebo untersuchte, war die Metaanalyse von Zeng et al. 2023 [487] und wurde für die Empfehlung zugrunde gelegt. Diese Metaanalyse beinhaltet auch das bislang größte RCT von Lamontagne et al. 2022 [488] zur hochdosierten Vitamin-C-Monotherapie bei Patienten mit Sepsis oder septischem Schock. Die gepoolten Ergebnisse von 8 RCTs mit 1394 Patienten zeigten, dass hochdosiertes Vitamin C mit einer Reduktion der Mortalität innerhalb von 30 Tagen bei Patienten mit Sepsis, nicht jedoch bei Patienten mit septischem Schock assoziiert ist. Darüber hinaus war hochdosiertes Vitamin C bei Patienten mit Sepsis, nicht aber bei Patienten mit septischem Schock, mit einer kürzeren Therapiedauer vasoaktiver Medikamente verbunden, jedoch ohne signifikanten Einfluss auf die Intensivliegedauer. Effektunterschiede zwischen hochdosiertem Vitamin C und Placebo zeigten sich weder im SOFA-Score 96 h nach Therapiebeginn noch in der 90-Tage-Mortalität im Vergleich, sowohl bei Patienten mit Sepsis als auch septischem Schock. [487]

Es sei an dieser Stelle darauf verwiesen, dass sich diese Empfehlung auf die hochdosierte intravenöse Vitamin-C-Therapie bezieht. Die Substitution des empfohlenen Tagesbedarfs von Vitaminen (und Spurenelementen) für gesunde Erwachsene bei nichtbilanzierter enteraler oder (rein) parenteraler Ernährung, im Rahmen der medizinischen Ernährungstherapie kritisch kranker Patienten mit Sepsis oder septischem Schock, bleibt davon unberührt.

### 8.10 Bikarbonat

#### 8.17/Empfehlung/Modifiziert (Stand 2025)

- EK
- Wir schlagen vor, bei Patienten mit septischem Schock Natriumbicarbonat alleinig zum Zwecke einer Verbesserung der Hämodynamik NICHT zu verwenden.
- SSC-Leitlinienadaption/Änderung des Empfehlungsgrades und Empfehlungstextes
- Konsensstärke: 100 %

Die Empfehlung der S3-Sepsis-Leitlinie 2018, dass Natriumbicarbonat grundsätzlich nicht bei Patienten mit septischem Schock eingesetzt werden soll, beruhte auf zwei kleinen randomisierten kontrollierten Studien, die keinen Vorteil hinsichtlich einer hämodynamischen Stabilisierung bzw. des Vasopressorbedarfs zeigen konnten [489, 490].

In einer multizentrischen randomisierten kontrollierten Studie aus dem Jahr 2018 wurden 400 Patienten mit einem pH ≤ 7,2 stratifiziert randomisiert und erhielten entweder Natriumbicarbonat mit einem Ziel-pH von 7,3 oder keine Therapie [491]. Im kombinierten Endpunkt aus 28-Tage-Mortalität und Organversagen an Tag 7 zeigte sich kein signifikanter Unterschied. Die Interventionsgruppe mit Natriumbicarbonat-Therapie erlitt allerdings häufiger eine Hypernatriämie, Hypokalzämie und metabolische Alkalose. Insgesamt hatten 61 % der eingeschlossenen Patienten einen septischen Schock, wobei die Anzahl an Patienten mit septischem Schock in den beiden Gruppen gleich verteilt war.

Eine Subgruppenanalyse der genannten Studienergebnisse zeigte bei Patienten mit akutem Nierenversagen (AKI Grad 2 und 3) bei Randomisierung (*n* = 182, 47 %) eine signifikant niedrigere 28-Tage.Sterblichkeit in der Interventionsgruppe (46 %) im Vergleich zur Kontrollgruppe (63 %) (RR 17,7 %; 95 % CI −33,0 bis −2,3; *p* = 0,016). Zudem wurde signifikant seltener ein Nierenersatzverfahren während des Intensivaufenthalts in der Gruppe mit Natriumbicarbonat-Therapie eingesetzt (35 vs. 52 %, *p* = 0,0009). Es zeigte sich eine signifikante Differenz zwischen Patienten mit einem AKIN-Score von 2 oder 3 im Vergleich zu denen mit einem Score von 0–1 (*p* = 0,023) [491].

Zusammenfassend ist die Evidenz basierend auf den vorhandenen Daten zum Einsatz bei AKI gering, und wir sprechen daher keine generelle Empfehlung für den Einsatz von Natriumbicarbonat in dieser Subgruppe aus. Basierend auf den genannten Daten sollte bei Patienten mit septischen Schock, metabolischer Azidose mit einem pH ≤ 7,2, und einem AKI (Grad 2 oder 3) die Gabe individuell mit Abwägung von Nebenwirkungen und potenziellem Nutzen erfolgen.

## 9. Behandlungsziele und Spätfolgen

Patienten, die eine Sepsis überlebt haben und längere Zeit intensivmedizinisch behandelt werden mussten, sind in der Regel mit einem langen und komplizierten Genesungsprozess konfrontiert. Es sind nicht nur Herausforderungen bei der körperlichen Rehabilitation zu bewältigen, sondern es besteht auch Verunsicherung darüber, wie die weitere Versorgung organisiert und koordiniert werden kann, um sowohl die Genesung zu fördern, aber auch Komplikationen zu vermeiden und sicherzustellen, dass die Versorgung auf die individuellen Präferenzen des Patienten und der Familie abgestimmt ist.

Es besteht ein breiter Konsens darüber, dass das derzeitige Gesundheitssystem erhebliche Defizite hinsichtlich der optimalen Versorgung dieser Patienten während der Genesungsphase aufweist. Es hat sich jedoch als außerordentlich schwierig erwiesen, eine solide Evidenzbasis zu generieren, auf der konkrete Empfehlungen zu Verbesserungen im Versorgungsparadigma abgegeben werden können.

Es gibt jedoch einige übergreifende konzeptionelle Merkmale einer „besten Praxis“, wobei zu berücksichtigen ist, dass die Art, der Zeitpunkt und die Kombination dieser breiten Aspekte der Versorgung variieren können und es bisher wenige evidenzbasierte Belege dafür gibt.

### 9.1 Behandlungsziele und Palliativversorgung

#### 9.1/Empfehlung/Bestätigt (Stand 2025)

- EK
- Wir empfehlen, dass die Behandlungsziele und die Prognose mit den Patienten bzw. deren Angehörigen/Betreuern besprochen werden.
- SSC-Leitlinienadoption
- Konsensstärke: 100 %

#### 9.2/Empfehlung/NEU (Stand 2025)

- EK
- Wir schlagen vor, bei Patienten mit Sepsis oder septischem Schock die Behandlungsziele möglichst früh (innerhalb von 72 h) festzulegen.
- SSC-Leitlinienadaptation/Änderung des Empfehlungsgrades
- Konsensstärke: 100 %

Die Sterblichkeitsrate von Sepsis-Patienten mit multiplem Organversagen ist hoch, und die Langzeitfolgen nach überlebter Sepsis sind häufig schwerwiegend. Das Ergebnis einer intensivmedizinischen Behandlung bei kritisch erkrankten Patienten exakt vorherzusagen, ist nicht möglich. Dennoch ist die Aufstellung realistischer Therapieziele von außerordentlicher Wichtigkeit [492]. Insbesondere, weil falsche Erwartungen bezüglich der Prognose bei den Angehörigen häufig sind. Eine vollumfängliche lebensverlängernde Intensivbehandlung steht nicht im Einklang mit dem Setzen von erstrebenswerten Behandlungszielen [493, 494]. Die Nutzung von Behandlungskonferenzen zur Identifizierung von Patientenverfügungen und von Behandlungszielen innerhalb von 72 h ab dem Zeitpunkt der Aufnahme auf der Intensivstation fördert nachweislich die Kommunikation und die Verständigung zwischen der Familie des Patienten und dem Behandlungsteam, verbessert die Angehörigenzufriedenheit, verringert Stress, Ängste und Depressionen bei den Angehörigen, erleichtert die Entscheidungsfindung zur Sterbebegleitung und verkürzt die Aufenthaltsdauer auf der Intensivstation bei Patienten, die versterben [495, 496]. Die Förderung einer gemeinsamen Entscheidungsfindung mit dem Patienten und dessen Angehörigen ist bei der Sicherstellung einer angemessenen Versorgung auf der Intensivstation und der Gewährleistung, dass überflüssige Behandlungsmaßnahmen vermieden werden, hilfreich [497, 498].

Die Palliativversorgung wird zunehmend als wesentlicher Bestandteil einer umfassenden Versorgung von kritisch kranken Patienten anerkannt, und zwar unabhängig von der Diagnose oder der Prognose [499]. Die Nutzung der Palliativmedizin auf der Intensivstation verbessert die Möglichkeiten zur Erkennung von Schmerzen und Ängsten, die Feststellung der Wünsche, Glaubensgrundsätze und Werte des Patienten und deren Einfluss auf die Entscheidungsfindung. Palliativmedizinische Konzepte fördern die Entwicklung von flexiblen Kommunikationsstrategien, die Festlegung von Behandlungszielen, die Bereitschaft zur Sterbebegleitung durch Angehörige, die Unterstützung bei der Lösung von Konflikten innerhalb des Teams und die Aufstellung von angemessenen Zielen für lebenserhaltende Maßnahmen [500].

Ein systematisches Review von palliativen Maßnahmen aus dem Jahre 2015 hat ergeben, dass Patienten, bei denen vollumfängliche Intensivbehandlungspläne und palliative Maßnahmen einbezogen wurden – trotz großer Abweichungen in Bezug auf den Studientyp und die Qualität – in 9 randomisierten kontrollierten Studien und 13 nichtrandomisierten kontrollierten Studien durchgängig ein Muster aufwiesen, welches auf einen Rückgang der Wiederaufnahmen und einen kürzeren Aufenthalt auf der Intensivstation hindeutet [501]. Zudem konnte 2022 in der randomisierten IMPACTS-Studie gezeigt werden, dass die Implementierung eines Maßnahmen-Bündels zur Optimierung des Entlassmanagements der Sepsis-Patienten vom Akutkrankenhaus in andere Versorgungseinrichtungen, welches auch palliativmedizinische Aspekte beinhaltete, sogar zu einem besseren Überleben führen kann [502, 503].

2024 hat die Europäische Gesellschaft für Intensivmedizin (ESICM) eine Leitlinie [504] zur Intensivtherapie am Lebensende und zur Palliativmedizin auf der Intensivstation veröffentlicht. Die Autoren empfehlen auf niedriger Evidenzstufe, dass zur Beurteilung von Symptomen bei Patienten mit fortgeschrittenen Erkrankungen zur besseren Behandlung validierte Instrumente eingesetzt werden sollten [504].

Die signifikanten Unterschiede zwischen einzelnen Krankenhäusern hinsichtlich der Bereitstellung von palliativen Maßnahmen stehen im Einklang mit früheren Studien, die Abweichungen bei der Versorgungsintensität am Lebensende nachgewiesen haben [505]. Trotz der Unterschiede in Bezug auf die jeweilige geografische Herkunft, die nationalen Rechtssysteme, die Religion und die kulturellen Präferenzen besteht ein weltweiter Konsens im Hinblick auf die wesentlichen intensivmedizinischen Praktiken am Lebensende [506].

### 9.2 Entlassmanagement und Nachsorge von Spätfolgen

#### 9.2.1 Post-Intensive-Care-Syndrom (PICS)

Das Post-Intensive-Care-Syndrom (PICS) beschreibt Beeinträchtigungen der physischen, kognitiven oder psychischen Funktionen, die nach einem Intensivaufenthalt erstmals oder verstärkt auftreten [507]. Es ist deshalb sinnvoll, Patienten nach Sepsis auf diese Folgeerkrankungen hin zu untersuchen, um ggf. ergo-, physio- und psychotherapeutische Behandlungen einleiten zu können [508]. Einige der häufigsten Beeinträchtigungen werden im Folgenden beschrieben.

#### 9.2.2 Delir

Der Begriff der septischen Enzephalopathie beschreibt eine diffuse organische Funktionsstörung des Gehirns bedingt durch die Sepsis. Dabei ist das Sepsis-assoziierte Delir das klinische Korrelat im Sinne eines hirnorganischen Psychosyndroms, das durch gleichzeitig bestehende Störungen des Bewusstseins, der Aufmerksamkeit, der Wahrnehmung, des Denkens, des Gedächtnisses, der Psychomotorik, der Emotionalität und des Schlaf-Wach-Rhythmus charakterisiert ist [362].

Zur Behandlung des Delirs auf der Intensivstation sei auf die „S3-Leitlinie Analgesie, Sedierung und Delirmanagement in der Intensivmedizin (DAS-Leitlinie)“ [362] verwiesen.

#### 9.2.3 Kognitive Dysfunktion

Die schwer verlaufende Sepsis ist mit einem substanziellen und persistierenden kognitiven Abbau und funktioneller Einschränkung vergesellschaftet [509]. Eine Studie von 821 Intensivpatienten mit respiratorischem Versagen und Schock konnte nachweisen, dass nach 12 Monaten 34 % der Patienten kognitive Defizite vergleichbar zu Patienten mit leichtem Schädel-Hirn-Trauma und sogar 24 % der Patienten kognitive Einbußen vergleichbar mit Patienten mit leichter Alzheimer-Erkrankung aufweisen [510].

Für das Erkennen der kognitiven Dysfunktion nach septischer Enzephalopathie eignet sich z. B. das Montreal Cognitive Assessment (MoCA) Instrument [511].

#### 9.2.4 Posttraumatische Belastungsstörung

Die posttraumatische Belastungsstörung (PTBS) ist eine spezifische Form von psychischen Traumafolgeerkrankungen, die nach einem schweren Verlauf einer Sepsis und in Zusammenhang mit einer intensivmedizinischen Behandlung auftreten können [512].

Die Punkt-Prävalenz von PTBS nach intensivmedizinischer Behandlung wird basierend auf Daten einer Metaanalyse [513] auf 4 % bis 62 % geschätzt und variiert dabei in Abhängigkeit von der Zeit nach ITS-Behandlung sowie dem Diagnoseinstrument. Für ein bis sechs Monate nach ITS-Behandlung wird die mittlere Prävalenz für klinisch relevante PTBS-Symptome mit 25 % (95 % CI 18 %–34 %) angegeben, für sieben bis 12 Monate werden entsprechend 17 % (95 % CI 10 %–26 %) berichtet. Die Komorbidität mit anderen psychischen Störungen wie Depression, Schlaf- oder substanzinduzierten Störungen sowie kognitiven Einschränkungen ist hoch [514–516]. Bei einem Teil der Patienten (18–26 %) kommt es zu einem protrahierten Verlauf (bzw. einem verzögerten Beginn) der PTBS [517, 518]. Psychische Vorerkrankungen und negative ITS-Erfahrungen erhöhen das PTBS-Risiko nach kritischen Erkrankungen [519]. Während der intensivmedizinischen Behandlung wird daher eine traumasensible Kommunikation („trauma-informed care“) durch das behandelnde Personal empfohlen [362]. Diese zielt auf die Reduktion von Anspannung und auf emotionale Unterstützung der Patienten (und Angehörigen) [520].

Zur Behandlung der PTBS sei auf die „S3-Leitlinie Posttraumatische Belastungsstörung“ [521] verwiesen. Spezifische Behandlungsansätze wie das Lesen von Intensivtagebüchern durch die Patienten nach ITS-Entlassung führt laut einer aktuellen Metaanalyse [522] zu einer signifikanten Reduktion der PTBS-Inzidenz im Zeitraum von 1 bis 3 Monaten nach ITS-Behandlung (7 RCTs; 1015 Patienten, OR 0,63; 95 % CI 0,45 bis 0,87; indirekte Evidenz, ITS-Population) [522]. Durch eine internetbasierte kognitiv-behaviorale Schreibtherapie konnte keine Reduktion der PTBS-Symptomatik bei Post-Sepsis-Patienten erreicht werden [523].

#### 9.2.5 Critical-Illness-Polyneuropathie/Critical-Illness-Myopathie (CIP/CIM)

Die neuromuskuläre Schwäche ist eine signifikante Komplikation längerer intensivmedizinischer Behandlung insbesondere bei kritischer Erkrankung, Sepsis und Multiorganversagen. Das klinische Hauptmerkmal ist die muskuläre Schwäche, die „ICU-acquired weakness“ (ICU-AW). Zum einen kommt es zu einem axonalen Schaden peripherer Nerven, der CIP (Critical-Illness-Polyneuropathie). Zum anderen kommt es zu einem Verlust dicker Myosinfilamente im Muskel, der CIM (Critical-Illness-Myopathie). Schließlich entwickeln sich Muskelatrophie und Kachexie. Häufig entwickelt sich eine Kombination aus all diesen Manifestationen. Auch die Entwicklung einer Schluckstörung ist im Verlauf zu bedenken [524].

Zwischen 50 und 100 % der Patienten mit Sepsis, septischem Schock und langer künstlicher Beatmung entwickeln eine CIP. Mindestens ein Viertel der Patienten mit Beatmung über sieben Tage oder mehr haben bereits klinische Zeichen einer CIP. Es wird angenommen, dass CIP, CIM und die Kombination aus beiden in etwa gleichen Anteilen vorkommen [524].

Zur Behandlung von Patienten auf der Intensivstation sei auf die „S3-Leitlinie Lagerungstherapie und Mobilisation von kritisch Erkrankten auf Intensivstationen“ [6] verwiesen.

#### 9.2.6 Entlassmanagement

##### 9.3/Empfehlung/NEU (Stand 2025)

- EK
- Wir empfehlen, dass Sepsis-Überlebende und deren Angehörige über das Beratungsangebot von Selbsthilfe-Gruppen informiert werden.
- SSC-Leitlinienadaptation/Änderung des Empfehlungsgrades
- Konsensstärke: 100 %

In Deutschland sind Krankenhäuser nach § 39 Absatz 1a des Fünften Buches Sozialgesetzbuch (SGB V) verpflichtet, ein effektives Entlassmanagement zur Unterstützung des Übergangs in die Anschlussversorgung zu gewährleisten. Dazu gehört auch der Verweis auf die Teilnahme in Selbsthilfe-Gruppen (siehe: Patienteninformation zum Entlassmanagement, https://www.gkv-spitzenverband.de/*).*

Das Potenzial der Selbsthilfe zur Bewältigung einer Erkrankung liegt im gegenseitigen Austausch von Betroffenen. Das Teilen von Erfahrungswissen ist ein Alleinstellungsmerkmal der der individualisierten und gruppenbasierten Selbsthilfe, das nicht durch ein professionelles medizinisches oder therapeutisches Angebot ersetzt werden kann. Teilnehmende in Selbsthilfegruppen (engl. „peer support groups“) erwerben im Vergleich zu Nichtnutzern erhöhte Selbstmanagementfähigkeiten und ein besseres krankheitsspezifisches Wissen [525].

Die Charakteristika einer mit einer intensivmedizinischen Behandlung verbundenen Langzeitmorbidität bei Sepsis-Überlebenden und deren Angehörigen wurde erstmals 2012 unter dem Begriff Post-Intensive-Care-Syndrome (PICS) zusammengefasst und näher definiert [507]. Mit der zunehmenden Akzeptanz des PICS in der Intensivmedizin wurde auch die potenzielle Bedeutung von Selbsthilfe-Gruppen als möglichem patientenzentriertem Ansatz zur Verbesserung der Beeinträchtigung kognitiver, psychischer und physischer Funktionen erkannt [526]. Wesentliche Faktoren des Einflusses von Selbsthilfe-Gruppen auf eine verbesserte Lebensqualität sind der Nutzen durch (1) das Wissen, dass andere ähnliche Erfahrungen teilen, und (2) die gemeinsame Bewältigung mit anderen Betroffenen [527].

Während Selbsthilfe-Gruppen in anderen Bereichen, vor allem bei onkologischen Erkrankungen, ein integraler Bestandteil einer professionellen medizinischen und psychosozialen Behandlung sind [528, 529], ist die Selbsthilfe bei Sepsis-Überlebenden und deren Angehörigen überwiegend noch unzureichend etabliert [526].

In Deutschland steht seit 2005 mit der Deutschen Sepsis-Hilfe e. V. (DSH) eine Patientenorganisation zur Verfügung, die Sepsis-Überlebende und Angehörige über eine kostenlose Hotline telefonisch (+49-700-73774-700) oder per E‑Mail (info@sepsis-hilfe.org) individuell berät. Informationsmaterialien, die in barrierearmer Sprache verfasst sind, stehen auf der DSH-Website https://sepsis-hilfe.org/de/ zur Verfügung und können den Akutkrankenhäusern auch als Informationsbroschüren zur Verfügung gestellt werden.

##### 9.4/Empfehlung/NEU (Stand 2025)

- EK
- Wir empfehlen, dass bei Sepsis-Überlebenden im Entlassbericht auf die Möglichkeit der Entwicklung eines Post-Intensive-Care-Syndroms (PICS) hingewiesen wird.
- SSC-Leitlinienadaptation/Änderung des Empfehlungsgrades und Empfehlungstextes
- Konsensstärke: 100 %

Der Übergang von der stationären Krankenhausversorgung in die hausärztliche, rehabilitative oder pflegerische Versorgung stellt eine besonders kritische Phase der Behandlungs- und Versorgungskette für Patienten mit überlebter Sepsis dar. Dieses gilt insbesondere für Spätfolgen, die aus dem Symptomkomplex des PICS auftreten können [530].

Die häufigsten PICS-assoziierten Erkrankungen sind neu aufgetretene neurokognitive Einschränkungen, eine Critical-Illness-Polyneuropathie und -myopathie (CIP/CIM) bzw. eine posttraumatische Belastungsstörung (PTBS) [514, 530]. Hierfür gibt es jedoch zumeist keine adäquate Diagnose zum Zeitpunkt der Krankenhausentlassung, weil die körperlich und mental kompromittierten Patienten zu diesem Zeitpunkt noch nicht für die aufwendigen neuropsychologischen Testungen empfänglich sind bzw. eine neurophysiologische Diagnostik (ENG/EMG) nicht erfolgte. Auch nach Entlassung aus dem Akutkrankenhaus erhalten die Patienten zumeist keine entsprechende Diagnostik, weil die nachbehandelnden Hausärzte in der Regel nicht über das erforderliche Wissen zum PICS-Komplex verfügen [531]. In einer qualitativen Studie hatten 63 % bzw. 81 % der Hausärzte keine Kenntnisse über ein PICS bei Patienten bzw. deren Angehörigen [532].

Intensivmediziner verfügen zwar über diese Kenntnisse, allerdings sind Informationen über bereits bestehende Symptome oder ein erhöhtes Risiko für ein PICS nur bei jedem dritten Sepsis-Überlebenden in den Entlassungsberichten der Akutkrankenhäuser enthalten [533, 534].

In der S3-Leitlinie „Sepsis – Prävention, Diagnose, Therapie und Nachsorge 2018“ [1] wurde bereits darauf hingewiesen, „die nachbehandelnden Ärzte im Postakut- und ambulanten Bereich auf diese Spätfolgen im Entlassungsbericht aus dem Krankenhaus aufmerksam zu machen“ [1]. Eine Diagnoseklassifikation des PICS ist in der Internationalen statistischen Klassifikation der Krankheiten und verwandter Gesundheitsprobleme (ICD) bisher nicht enthalten [535], sodass gegenwärtig nur Symptome klassifikatorisch benannt werden können (Tab. 7).

**Tab. 7:** Anwendungsbeispiel für einen standardisierten Textblock von Entlassungsberichten bei Sepsis-Überlebenden

*„Hinweis für die nachbehandelnden Ärztinnen und Ärzte: Ihr Patient litt an einer Sepsis mit schwerem Verlauf, der eine Intensivtherapie erforderlich machte. Obwohl die Akutphase der Erkrankung überstanden ist, besteht das Risiko der Entwicklung eines sogenanntes Post-Intensive-Care-Syndroms (PICS), oft auch mit großer Verzögerung. Ein PICS kann vielfältige Probleme auslösen: physisch (Anhaltende Schwäche, Müdigkeit, Schmerzen), kognitiv (Gedächtnis- und Konzentrationsprobleme) und psychisch (posttraumatische Belastungsstörung. Angststörungen. Depression). Bitte weisen sie Ihren Patienten auf die Möglichkeit der Kontaktaufnahme mit Selbsthilfegruppen hin (Deutsche Sepsis-Hilfe e.* *V.: *https://sepsis-hilfe.org*).“*	
Erkrankung	ICD-10-GM-2024
Kognitive Dysfunktion	U51.–
Posttraumatische Belastungsstörung	F43.1
Polymyopathie/Polyneuropathie	G72.80, G62.80
Energie- und Eiweißmangelernährung	E43

#### 9.2.7 Nachsorgeprogramme und Rehabilitation

Die Surviving Sepsis Campaign 2021 [2] hat erstmals eine Empfehlung für ambulante Nachsorgeprogramme nach Intensivaufenthalt mit einer schwachen Empfehlung und sehr niedrigen Qualität der Evidenz ausgesprochen. In Deutschland werden diese Programme als Ambulanz für Intensivmedizinische Nachsorge oder PICS-Ambulanz bezeichnet und zunehmend angeboten. Ein Cochrane-Review aus dem Jahr 2018 konnte auf der Grundlage von 5 Studien keine Evidenz für den Nutzen von derartigen intensivstationären Nachsorgeprogrammen nachweisen [536]. Seither wurde eine randomisierte kontrollierte Studie von Sharshar et al. [537] veröffentlicht, bei der ein multidisziplinäres Beratungsangebot mit einer signifikant höheren Mortalität und Verschlechterung der Lebensqualität, insbesondere von Angst und Depression im Vergleich zur Standardnachsorge, verbunden war [537]. Auf der anderen Seite haben Taylor et al. [502] in einer randomisierten kontrollierten Studie nachgewiesen, dass ein 30-Tage-Beratungsprogramm durch eine Pflegekraft zu einer niedrigeren Mortalität und Rehospitalisierungsrate führen kann [502]. Aufgrund dieser unterschiedlichen Ergebnisse ist es aktuell nicht möglich, eine Empfehlung für oder gegen ein Nachsorgeprogramm nach Intensivaufenthalt für Sepsis-Patienten auszusprechen. Die Leitliniengruppe möchte allerdings betonen, dass in generellen Kohorten von Intensivüberlebenden mehrfach deutliche Einschränkungen in den kognitiven, psychologischen und funktionellen Domänen nachgewiesen wurden, die unter dem Begriff Post-Intensive-Care-Syndrom (PICS) zusammengefasst werden [507, 538]. Die Häufigkeit der Einschränkungen wird dabei mit mind. 50 % angegeben [510, 539–543].

In mehreren randomisierten Studien wurde der Stellenwert von Rehabilitationsprogrammen für Überlebende einer kritischen Erkrankung untersucht [544–552]. Diese Studien konzentrierten sich auf kritisch kranke Patienten mit mehrtägiger ITS-Liege- bzw. Beatmungsdauer. Dabei zeigten sich geringfügige Verbesserungen der Lebensqualität und der depressiven Symptome, aber kein Unterschied in der Mortalität, der körperlichen Funktion oder der Angst. Dennoch können aufgrund des überzeugenden Nutzens von stationären Rehabilitationsprogrammen bei anderen Erkrankungen [553] (z. B. ältere Patienten mit kognitiven Beeinträchtigungen, Patienten nach Schlaganfall oder traumatischer Hirnverletzung) Überlebende einer Sepsis von der Verlegung in Rehabilitationseinrichtungen profitieren. Dieser Vorschlag steht im Einklang mit den Leitlinien mehrerer Expertengremien [554–556]. Zukünftige Forschung ist erforderlich, um einen optimalen Ansatz für die funktionelle Rehabilitation (Zeitpunkt, Dosierung, Intensität, Dauer) und die Patientenauswahl zu bestimmen [557].

Das Literaturupdate der Leitliniengruppe hat lediglich eine weitere relevante, jedoch retrospektive Studie basierend auf Daten einer deutschen Krankenkassen-Daten identifiziert, die jedoch nicht nur Intensivpatienten, sondern generell Krankenhauspatienten untersuchte. In diesen Daten von ca. 42.000 Sepsis-Patienten zeigt sich ein Hinweis auf ein besseres Langzeitüberleben in der Rehabilitationsgruppe [558]. Dieses spiegelt sich auch in einer weiteren retrospektiven Analyse von Daten einer anderen deutschen Krankenkasse wider, in die allerdings nicht nur Patienten mit Sepsis und septischem Schock, sondern auch schweren Infektionen eingeschlossen wurden [559].

## 10. Präventionsmaßnahmen und Impfungen

### 10.1 Maßnahmen zur Infektionsprävention

Die Leitlinienkommission hat einvernehmlich entschieden, der Prävention nosokomialer Infektionen wegen ihrer großen Bedeutung ein eigenes Kapitel zu widmen. In den Empfehlungen der SSC-Leitlinie 2021 werden Maßnahmen zur Prävention von nosokomialen Infektionen nicht separat aufgegriffen.

Zur Prävention von nosokomialen Infektionen, die zu einer Sepsis führen können, wie auch zur gefäßkatheterassoziierten Sepsis existieren diverse spezifische Leitlinien und Empfehlungen. Für Deutschland sind hier insbesondere die Empfehlungen der Kommission für Infektionsprävention in medizinischen Einrichtungen und in Einrichtungen und Unternehmen der Pflege und Eingliederungshilfe (KRINKO) zu erwähnen.

Zudem veröffentlichte die Weltgesundheitsorganisation (World Health Organisation, WHO) 2016 Leitlinien zu den Kernkomponenten der Infektionsprävention, welche später von Storr und Kollegen aktualisiert wurden [560, 561]. Diese Leitlinien wurden nach den Anforderungen des WHO-Handbuches für die Leitlinienentwicklung erarbeitet [562] und waren eine Weiterentwicklung der evidenzbasierten Leitlinien der SIGHT-Gruppe, die Fachliteratur mit Publikationsdatum von 1996 bis 2012 berücksichtigte [563]. Durch die WHO-Arbeitsgruppe wurden entsprechende PICO-Fragen formuliert, und es wurde ein weiteres systematisches Review durchgeführt, das Studien bis 2015 hinzuzog [564]. Zur Bewertung der Studien wurden die für die Cochrane-EPOC-Reviews entwickelten Kriterien angewendet [565]. Ein weiteres systematisches Review aus dem Jahr 2023 ergänzte die Literatur aus den Jahren 2017 bis 2021 [566].

Die deutsche Sepsis-Leitlinienkommission bewertete die WHO-Kernkomponenten erneut in Bezug auf deren Adaptation. Dabei wurde insbesondere geprüft, ob sich Aussagen zu den Empfehlungen der WHO-Kernkomponenten bereits in anderen nationalen Regelungen mit hohem Umsetzungsgebot (KRINKO-Empfehlungen, Länderhygieneverordnungen, Infektionsschutzgesetz) finden. In diesen Fällen wurde entschieden, auf die Darstellung der WHO-Kernkomponenten in der Sepsis-Leitlinie zu verzichten. Es wird daher an dieser Stelle ausdrücklich darauf hingewiesen, dass die Infektionsprävention in Deutschland in hohem Maß auch durch Gesetze reguliert ist und die Kenntnis und Beachtung dieser Vorgaben die Grundlage der Infektionsprävention und damit auch der Sepsis-Prävention darstellen. Eine weitere Empfehlung zur Implementierung eines Antibiotic-Stewardship(ABS)- bzw. Antimicrobial-Stewardship(AMS)-Programms wurde in die Sepsis-Leitlinie integriert.

#### 10.1/Empfehlung/Bestätigt (Stand 2025)

- EK
- Wir empfehlen die Implementierung eines Antimicrobial-Stewardship(AMS)-Programms zur Sicherung einer rationalen Antiinfektiva-Anwendung im Krankenhaus.
- Zusätzliche DSG-Leitlinienempfehlung
- Konsensstärke: 100 %

Ein ABS-Programm wird auch in einem Positionspapier [567] der Kommission Antiinfektiva, Resistenz und Therapie Kommission (ART) empfohlen.

#### 10.2/Empfehlung/Modifiziert (Stand 2025)

- EK
- Wir empfehlen, multimodale Strategien zur Implementierung von Infektionspräventionsmaßnahmen (z. B. bei intravasalen Kathetern) einzusetzen.
- WHO-Leitlinienadaptation/Änderung des Empfehlungsgrades
- Konsensstärke: 100 %

Innerhalb von multimodalen Strategien werden verschiedene Implementierungsansätze (üblicherweise 5: Bereitstellung einer geeigneten Infrastruktur, Aus- und Weiterbildung, Monitoring von Infrastruktur, Prozessen und Ergebnisqualität, Kommunikation und Kulturwandel) zu einem Gesamtkonzept verbunden und beinhalten häufig Bundles oder Checklisten, die durch multidisziplinäre Teams entsprechend den lokalen Bedingungen entwickelt werden. Ziel hierbei ist das Verhalten der Mitarbeitenden im Hinblick auf notwendige Verbesserungen zu beeinflussen und zu einer Veränderung der Organisationskultur beizutragen. Solche multimodalen Strategien sind insbesondere bei der Verbesserung der Händehygiene-Compliance, der Reduktion von mit zentralen Venenkathetern (ZVK-)assoziierten Blutstrominfektionen und Beatmungs-assoziierten Pneumonien relevant, aber auch bei der Senkung der Zahl von Infektionen mit Methicillin-resistentem *Staphylococcus aureus* (MRSA) und *Clostridioides difficile* (*C. difficile*) [561].

Die optimale Anzahl oder Art der in multimodalen Strategien eingesetzten Interventionen ist bis heute jedoch nicht bekannt [564].

In Deutschland kann die Evidenz für verschiedene Einzelmaßnahmen, welche Gegenstand solcher multimodalen Strategien sein können, den jeweiligen KRINKO-Empfehlungen entnommen werden.

### 10.2 Impfungen

#### 10.3/Empfehlung/Modifiziert (Stand 2025)

- EK
- Wir empfehlen die Umsetzung der Empfehlungen der Ständigen Impfkommission (STIKO) am Robert Koch-Institut, insbesondere die Impfungen gegen Influenza, COVID-19, RSV (Respiratory-Syncytial-Virus), Pneumokokken, Meningokokken und Haemophilus influenzae B.
- Wir empfehlen für das medizinische Personal die jährliche Impfung gegen Influenza und COVID-19, um Patienten vor nosokomialen respiratorischen Infektionen und konsekutiver Sepsis zu schützen.
- Zusätzliche DSG-Leitlinienempfehlung
- Literatur: [568]
- Konsensstärke: 100 %

#### 10.4/Empfehlung/NEU (Stand 2025)

- EK
- Wir empfehlen, dass Patienten mit Asplenie gemäß STIKO-Empfehlungen geimpft, mit einem entsprechenden Notfallpass und mit einer Antibiotika-Standby-Prophylaxe ausgestattet werden.
- Zusätzliche DSG-Leitlinienempfehlung
- Literatur: [568]
- Konsensstärke: 100 %

In den Empfehlungen der SSC-Leitlinie 2021 werden Impfungen nicht separat erwähnt. Da Impfungen jedoch eine wesentliche Präventionsmaßnahme zur Reduktion von Mortalität und Morbidität schwerer Infektionen und damit der Sepsis darstellen, ist aus Sicht der Leitlinienkommission ein Verweis erforderlich.

Um vulnerable Patienten vor nosokomialer Influenza und einer COVID-19-Infektion, die mit einer relevanten Mortalität assoziiert ist, zu schützen, wird dem medizinischen Personal jährlich zu Beginn der Saison eine Impfung mit den jeweils empfohlenen Vakzinen empfohlen [568].

Die ambulant erworbene und die nosokomial erworbene Pneumonie sind der häufigste Fokus bei Patienten mit Sepsis oder septischem Schock [569]. Die häufigsten viralen Erreger und der häufigste bakterielle Erreger der ambulant erworbenen Pneumonie, Influenzavirus, COVID-19 und RSV bzw. *Streptococcus pneumoniae* (Pneumokokken), sind impfpräventabel. Viren und Bakterien können gemeinsam als Mischinfektion bzw. als bakterielle Superinfektion auftreten – zunehmend werden auch Mischinfektionen mit Pneumokokken und COVID-19 gefunden [570]. Bei diesen Infektionen steigt die Mortalität gegenüber der Monoinfektion deutlich an [571]. Die genannten impfpräventablen viralen und bakteriellen Erreger verursachen auch einen wesentlichen Teil der nosokomialen Pneumonie. Die zunehmende Erkenntnis, dass nosokomiale Influenza, COVID-19 und RSV ein relevantes Problem darstellt, findet sich auch in der aktuellen Leitlinie zur nosokomialen Pneumonie [210]. Eine Erhebung an einem deutschen Universitätsklinikum in der Saison 2014/15 zeigte, dass 35 % aller Nachweise nosokomialen Ursprungs waren; die Mortalität betrug 10 % [572] Daher unterstützt die Leitlinienkommission nachdrücklich die Empfehlung zur jährlichen Impfung des medizinischen Personals.

Ein besonders hohes Risiko für die Entwicklung einer Sepsis haben Patienten mit einer anatomischen oder funktionellen Asplenie. Sie sind gefährdet, eine rapid verlaufende Sepsis („overwhelming post-splenectomy infection syndrome“, OPSI) durch bekapselte Bakterien zu erleiden – v. a. Pneumokokken, Meningokokken und bekapselte *H.-influenzae-*Stämme. Diese sollten mit einem Asplenie-Notfallpass (erhältlich unter *www*.asplenie-net.org*)* ausgestattet werden, in dem auch eine Dauerprophylaxe mit Penicillin oder eine Standby-Antibiotikaprophylaxe Amoxicillin/Clavulansäure (bessere Wirksamkeit bei *H. influenzae*) empfohlen wird. Der Vorteil einer Prophylaxe mit Penicillin bei Kindern mit Asplenie wurde auch in einem Cochrane-Review bestätigt [573].

Ein relevanter Anteil der Sepsis-Patienten wurde bereits im vorhergehenden Jahr wegen einer Infektion hospitalisiert. Dennoch ist Großteil der Intensivüberlebenden unzureichend geimpft [574]. Daher sollte bei Patienten, die eine Sepsis oder einen septischen Schock überlebt haben, der Impfstatus im Rahmen der Nachsorge überprüft und komplettiert werden, dies gilt insbesondere für die jährliche Influenzaimpfung.

## 11. Wichtige Forschungsfragen

Angesichts fehlender Evidenz für zahlreiche Maßnahmen zur Versorgung von kritisch kranken Patienten mit Sepsis oder septischem Schock wurden alte und neue Wissenslücken offenbart. Von insgesamt 56 evidenzbasierten Empfehlungen war die zugrunde liegende „*certainty of evidence*“ nach GRADE (Evidenzqualität) nur bei 5 Empfehlungen hoch und bei 18 Empfehlungen moderat. Bei 17 Empfehlungen war die Evidenzqualität niedrig und bei 16 sehr niedrig.

Diese Evidenzlücken können nur durch zukünftige multizentrische nichtkommerzielle klinische Prüfungen (IIT, „investigator-initiated trials“) geschlossen werden. Zum überwiegenden Teil sind dieses Therapieoptimierungsstudien (TOS) zu zugelassenen Arzneimitteln bzw. Medizinprodukten, welche im Rahmen einer klinischen Prüfung miteinander verglichen werden. Hier sind zusätzliche Forschungsförderprogramme seitens öffentlicher Förderinstitutionen (BMBF, DFG) zwingend erforderlich, da die Beantwortung dieser Fragen genuin nicht im kommerziellen Interesse der Industrieforschung ist.

Besonders bei schwer erkrankten Patienten mit Sepsis oder septischem Schock ist die bisherige Forschung in Deutschland durch kleine klinische Studien mit unzureichender Aussagekraft gekennzeichnet, oft mit schwacher Methodik. Nur groß angelegte Phase-III-Studien können rasch qualitativ hochwertige Daten liefern, um die medizinische Praxis zu verändern. Dazu gehören große nationale und internationale Plattformstudien, die in vielen Ländern eine große Anzahl von Patienten mittels eines pragmatischen und adaptiven Designs rekrutieren können. Diese Plattformstudien werden als „investigator-initiated trials“ mit öffentlicher Förderung durchgeführt und erlauben aufgrund ihres response-adaptiven Designs rasche Rückschlüsse auf die Überlegenheit bzw. Unterlegenheit eines Studienarmes bzw. einer Intervention.

## 12. Zusammensetzung der Leitliniengruppe

### 12.1 Leitlinienkoordinator/Ansprechpartner

#### Leitlinienkoordinator

Prof. Dr. med. Frank M. Brunkhorst

Klinik für Anästhesiologie und Intensivtherapie

Universitätsklinikum Jena

07740 Jena

Deutsche Sepsis-Gesellschaft e. V. (DSG)

Waldenserstr. 2–4

10551 Berlin

Email: frank.brunkhorst@med.uni-jena.de

Leitliniensekretariat.

Stefanie Truschzinski

Deutsche Sepsis-Gesellschaft e. V. (DSG)

#### Geschäftsstelle

c/o Conventus Geschäftsstellenmanagement

Carl-Pulfrich-Str. 1

07745 Jena

Telefon: +493641-3116444

Email: geschaeftsstelle@sepsis-gesellschaft.de

### 12.2 Beteiligte Fachgesellschaften und Organisationen

Die Arbeitsabläufe und Verantwortlichkeiten im Leitlinienprojekt sind im Organigramm aufgeführt (vgl. Leitlinienreport unter https://register.awmf.org/de/leitlinien/detail/079-001). Die Steuerungsgruppe (LL-steering) koordinierte zusammen mit dem Leitlinienbüro (LL-office) alle Prozesse der Leitlinienarbeit. Die Leitliniengruppe wurde in 9 Arbeitsgruppen (AG-member) aufgeteilt, die jeweils von zwei Koordinatoren (AG-chair) geleitet wurden. Die Koordinatoren waren für einen termingerechten Arbeitsablauf innerhalb ihrer Arbeitsgruppe verantwortlich und bereiteten redaktionell die Leitlinientexte (Begründungen, Literatur) sowie einen Vorschlag für die Empfehlungen auf der Grundlage der neu bewerteten Evidenz durch die Leitlinienmethodiker (LL-Methodik) vor. LL-Methodiker waren Prof. Dr. med. Frank Brunkhorst, Dipl.-Ges.oec. Agnieszka Lajca und Dr. med. Vladimir Patchev (systematische Literaturrecherche, methodische Bewertung der Literatur, Evidenzsynthese, Evidenzprofile nach GRADE).

An der Leitlinienerstellung hat die Deutsche Gesellschaft für Fachkrankenpflege und Funktionsdienste e. V. (DGF) nicht teilgenommen. Diese wurde zu Beginn der Leitlinienerstellung mehrfach angefragt, konnte aber keine Mandatsträger zur Leitlinienerstellung entsenden.

Von der formalen Beteiligung der Deutschen Gesellschaft für Kardiologie – Herz- und Kreislaufforschung e. V. (DGK) und der Deutschen Gesellschaft für Innere Medizin e. V. (DGIM) wurde abgesehen, da Vertreter ihrer Schwerpunktgesellschaften, der Deutschen Gesellschaft für Internistische Intensivmedizin und Notfallmedizin (DGIIN), der Deutschen Gesellschaft für Infektiologie (DGI), der Deutschen Gesellschaft für Nephrologie e. V. (DGfN), der Deutschen Gesellschaft für Pneumologie und Beatmungsmedizin (DGP) und der Deutschen Gesellschaft für Hämatologie und. Medizinische Onkologie e. V (DGHO) im Leitlinienkomitee vertreten waren.

### 12.3 Patientenbeteiligung

Die Sicht von Patienten und Medizinern auf eine Erkrankung unterscheidet sich häufig. Während die Behandelnden das Überleben („survival rate“) zu Recht in den Mittelpunkt ihrer Bemühungen stellen, stellt für Patienten die gesundheitsbezogene Lebensqualität („survivorship“) im Fokus. Bei den meisten Sepsis-Patienten ist eine prolongierte Intensivtherapie erforderlich, die Langzeitfolgen verursacht, welche die Lebensqualität der Patienten einschränken und das Behandlungsergebnis verschlechtern kann. Dies bezeichnet man als Post-Intensive-Care-Syndrom (PICS) [507]. Einen besonderen Schwerpunkt dieser Leitlinie stellen daher die Einschränkungen der gesundheitsbezogenen Lebensqualität dar, welche im Kapitel „Nachsorge“ gemeinsam mit der Patienten- bzw. Angehörigenorganisation „Deutsche Sepsis-Hilfe“ adressiert werden.

Die Leitlinie wurde unter direkter Beteiligung von Patientenvertretern erstellt. Herr Prof. Frank Brunkhorst, stellvertr. Vorsitzender und Frau Babila Janusan, Geschäftsführerin der Deutschen Sepsis-Hilfe e. V. (https://sepsis-hilfe.org/de/), waren stimmberechtigt und vom 01.11.2023 bis 30.04.2025 an der Erstellung der Leitlinie beteiligt.

### 12.4 Methodische Begleitung

Bei der Aktualisierung wurde die Leitlinie durch Frau Dr. rer. biol. hum. Cathleen Muche-Borowski, AWMF-Leitlinienberaterin, methodisch begleitet.

## 13. Informationen zu dieser Leitlinie

### 13.1 Methodische Grundlagen

Die Methodik zur Erstellung dieser Leitlinie richtet sich nach dem AWMF-Regelwerk (Version 2.1 vom 05.09.2023) [575]. Eine ausführliche Beschreibung des methodischen Vorgehens befindet sich im Leitlinienreport auf der AWMF-Website der Leitlinie: https://register.awmf.org/de/leitlinien/detail/079-001.

Quelle: AWMF, Ständige Kommission Leitlinien (2023): Das AWMF-Regelwerk „Leitlinien“. Marburg: Arbeitsgemeinschaft der Wissenschaftlichen Medizinischen Fachgesellschaften, Ständige Kommission Leitlinien. URL: https://www.awmf.org/fileadmin/user_upload/dateien/downloads_regelwerk/20230905_AWMF-Regelwerk_2023_V2.1_final.pdf (Zugriffsdatum: 09.03.2025).

### 13.2 Systematische Recherche und Auswahl der Evidenz

Den Arbeitsgruppen oblag die Prüfung der Aktualität der Empfehlungen und der zugrunde liegenden Evidenz bzw. Formulierung zusätzlicher im Fachgebiet relevanter, neuer Fragestellungen nach dem PICO-Prinzip (Tab. 8).

**Tab. 8:** Beispiel PICO-Fragestellung

Should prolonged infusion of beta-lactams (maintenance) vs. bolus infusion of beta-lactams (maintenance) be used in adults with sepsis or septic shock?				
Population	Intervention	Comparator	Outcome(s)	Setting
Patient/Population bzw. Problem: Um welche Patienten, Population geht es, gibt es Subgruppen zu berücksichtigen?	Welche Intervention ziehe ich in Betracht und welche (Qualitäts‑)Kriterien muss sie erfüllen?	Vergleichsintervention: Welche Alternative(n) gibt es und welche Kriterien müssen diese erfüllen?	Ergebnisparameter: Welche Ziele möchte ich erreichen?	Setting, Studientyp, Zeitraum
Adult patients with sepsis or septic shock	*Prolonged* infusion of beta-lactams (maintenance)	*Bolus infusion* of beta-lactams (maintenance)	Mortality, clinical cure rate	ICU, hospital ward

Design: systematische Übersichtsarbeiten/Metaanalysen

Für ausgewählte Empfehlungen bzw. PICO-Fragen wurde eine iterative, hierarchische, systematische Recherche durchgeführt. Alle Recherchen wurden im Laufe des Jahres 2024 durchgeführt. Das jeweilige Datum der Suche wurde im Leitlinienreport dokumentiert.

Der Zeitraum der Suche ist von der Quelle der Empfehlung abhängig:

- Adaptierte SSC-2021-Empfehlungen: Juli 2019 (Recherchestichdatum der SSC 2021) bis Datum der Suche
- Eigene DSG-Empfehlungen: Dezember 2018 (Recherchestichdatum der DSG 2018) bis Datum der Suche
- Adaptierte Empfehlungen anderer Quellleitlinien: jeweiliger Recherchestichdatum bis Datum der Suche
- Neue Empfehlungen: Zeitraum der Suche wurde mit der Fachgruppe abgesprochen: z. B. letzte 20 Jahre bis Datum der Suche

Die Suche erfolgte in mindestens zwei elektronischen Datenbanken (Medline, Cochrane Library), LL-Datenbanken und Websites von Leitlinienorganisationen und themenbezogenen Fachgesellschaften sowie Websites der HTA- und IQWiG-Berichte.

Ziel des Suchverfahrens war es, die jeweils aktuellste und qualitativ hochwertigste aggregierte Evidenz zu ermitteln. Daher wurden im ersten Schritt kürzlich veröffentlichte und hochwertige evidenzbasierte Leitlinien und systematische Übersichtsarbeiten zu RCTs gesucht.

Die Strategien enthielten den Themenblock Population: „sepsis OR septic shock OR critical*“ und weitere Strategiemodule (unter Verwendung der Mesh Terms, Synonyme, Trunkierung und alternativen Schreibweisen) die mittels Boole’scher Operatoren miteinander verknüpft wurden.

Die Auswahl der Evidenz erfolgte in 2‑stufigem Screeningprozess auf (1) Titel/Abstract- und (2) Volltext-Ebene, unter Berücksichtigung der für jede PICO-Fragestellung vordefinierten Ein- und Ausschlusskriterien, inkl. der Dokumentation des Screeningprozesses im PRISMA-Diagramm.

Eine ausführliche Beschreibung der systematischen Recherche und Auswahl der Evidenz befindet sich im Leitlinienreport dieser Leitlinie.

### 13.3 Kritische Bewertung der Evidenz

Die kritische Bewertung der methodischen Qualität der eingeschlossenen Publikationen wurde mittels von Cochrane Deutschland und der AWMF empfohlenen validierten Instrumenten [576, 577] in Abhängigkeit von den jeweiligen Studiendesigns und von 2 unabhängigen Methodikern durchgeführt (Leitlinien: AGREE-II-Instrument, systematische Übersichtsarbeiten und Metaanalysen: AMSTAR-Checkliste).

Um das Vertrauen in die Schätzung des Effekts zusammenzufassen und damit eine Empfehlung zu stützen, verwendete das Leitlinien-Methodik-Team mit Input der Koordinatoren der Arbeitsgruppen den GRADE-Ansatz [578]. Die Qualität der Evidenz wurde dabei als hoch, moderat, niedrig oder sehr niedrig bewertet [579]. Zur Erstellung von Evidenzprofilen („summary of evidence“) wurde die Online-Software „Guideline Development Tool (GDT)“ (http://gdt.guidelinedevelopment.org) verwendet [580]. Die Qualität der Evidenz beeinflusste die Stärke der Empfehlungen [3]. Der GRADE-Ansatz basiert auf der Bewertung der Evidenz anhand von sechs Bereichen: (1) Risiko einer Verzerrung, (2) Inkonsistenz, (3) Indirektheit, (4) Ungenauigkeit, (5) Publikationsverzerrung und (6) andere Kriterien. Eine ausführliche Beschreibung zur Bewertung der Evidenz befindet sich im Leitlinienreport dieser Leitlinie.

### 13.4 Strukturierte Konsensfindung

Die strukturierte Konsensfindung erfolgte im Rahmen einer Konsensuskonferenz in Anlehnung an den NIH-Typ unter unabhängiger Moderation. Eine ausführliche Beschreibung der strukturierten Konsensfindung ist im Leitlinienreport dieser Leitlinie aufgeführt.

### 13.5 Empfehlungsgraduierung und Feststellung der Konsensstärke

#### Festlegung des Empfehlungsgrades.

Die evidenzbasierten Empfehlungen der Leitlinie sind mit Empfehlungsgrad und Evidenzqualität nach GRADE und Oxford 2011 gekennzeichnet.

Neben der methodisch aufbereiteten Evidenz wurden bei der Graduierung der Empfehlung die klinische Erfahrung und die Patientenpräferenz berücksichtigt. Zusätzlich wurden weitere Kriterien wie die Konsistenz der Studienergebnisse, die klinische Relevanz der Endpunkte und Effektstärken, das Nutzen-Schaden-Verhältnis, ethische, rechtliche, ökonomische Verpflichtungen, Patientenpräferenzen, die Anwendbarkeit auf die Patientenzielgruppe und das deutsche Gesundheitssystem sowie die Umsetzbarkeit im Alltag bzw. in den verschiedenen Versorgungsbereichen bei der Graduierung der Empfehlung berücksichtigt (Tab. 9).

**Tab. 9:** Zweistufiges Schema zur Graduierung von Empfehlungen nach GRADE

Empfehlungsgrad	Beschreibung	Ausdrucksweise	Symbol (fakultativ)
1	Starke Empfehlung	Wir empfehlen/empfehlen nicht	⇑⇑/⇓⇓
2	Schwache Empfehlung	Wir schlagen vor/schlagen nicht vor	⇑/⇓

Quelle: AWMF S3-Template (URL: https://www.awmf*.org/regelwerk/downloads*)

#### Feststellung der Konsensstärke.

Die Konsensstärke wurde gemäß Tab. 10 klassifiziert.

**Tab. 10:** Festlegung der Konsensstärke

Klassifikation der Konsensusstärke	
Starker Konsens	> 95 % der Stimmberechtigten
Konsens	> 75–95 % der Stimmberechtigten
Mehrheitliche Zustimmung	> 50–75 % der Stimmberechtigten
Keine mehrheitliche Zustimmung	< 50 % der Stimmberechtigten

Quelle: AWMF S3-Template (URL: https://www.awmf.org/regelwerk/downloads)

Ein Konsens mit Annahme der Empfehlung ist bei einer Zustimmung von > 75 % der abstimmenden Mandatstragenden erzielt. Enthaltungen aufgrund von Interessenkonflikten werden von der Grundgesamtheit der abstimmenden Mandatstragenden abgezogen.

## 14. Redaktionelle Unabhängigkeit

### 14.1 Finanzierung der Leitlinie

Die Leitlinie wurde gefördert aus Mitteln des Innovationsfonds zur Förderung von Versorgungsforschung (§ 92a Abs. 2 Satz 1 SGB V) für das Projekt: S3-Sepsis – Update S3-Leitlinie „Sepsis – Prävention, Diagnose, Therapie und Nachsorge“, Förderkennzeichen: 01VSF23012, Themenfeld 4: Intensivmedizinische Versorgung schwerstkranker Patientinnen und Patienten (https://innovationsfonds.g-ba.de/projekte/versorgungsforschung/s3-sepsis.588).

### 14.2 Darlegung von Interessen und Umgang mit Interessenkonflikten

Die Angaben zu den Interessen wurden mit dem AWMF-Formblatt von 2018 erhoben und von einem COI-Ausschuss, der sich aus den Mitgliedern der Steuerungsgruppe (Prof. Brunkhorst, Prof. Kaasch, Prof. Weigand, Prof. Adamzik, Prof. Pletz, Prof. Meybohm) zusammensetzte, auf einen thematischen Bezug zur Leitlinie bewertet. Als geringer Interessenkonflikt wurden hierbei Vortragstätigkeiten, als moderater Interessenskonflikt Tätigkeit in einem industriefinanzierten Advisory Board und/oder eine Funktion als Principle Investigator in einem industriefinanzierten Forschungsvorhaben und als hoher Interessenkonflikt Eigentümerinteressen und Aktienbesitz kategorisiert. Ein geringer IKE hatte keine Konsequenz zur Folge, da ein Zusammenhang mit dem Leitlinienthema nicht erkennbar. Ein moderater Interessenkonflikt hatte eine Stimmenthaltung zur Konsequenz. Ein hoher Interessenkonflikt führte zum Ausschluss von der Beratung und Abstimmung zum betreffenden Thema. Alle Offenlegungen wurden überprüft, bevor die Leitlinien-Panellisten ihre Arbeit aufnahmen, und beeinflussten die Zuweisung zu den einzelnen Panel-Arbeitsgruppen.

Als protektive Faktoren, die einer Verzerrung durch Interessenkonflikte entgegenwirken, können die pluralistische Zusammensetzung der Leitliniengruppe, die strukturierte Konsens-findung unter neutraler Moderation, die Diskussion zu den Interessen und Umgang mit Interessenkonflikten zu Beginn der Konsenskonferenz und eine öffentliche Konsultationsfassung gewertet werden.

## 15. Verabschiedung durch die Vorstände der herausgebenden Fachgesellschaften/Organisationen

Die Leitliniendokumente wurden am 10.04.2025 den beteiligten Fachgesellschaften/Organisationen übergeben. Die Verabschiedung durch die Vorstände der herausgebenden Fachgesellschaften/Organisationen erfolgte seitens der/des:

- DGHO: am 14.04.2025
- DIVI: am 29.04.2025
- PEG: am 11.04.2025
- NRZ: am 11.04.2025
- DGEM: am 13.04.2025
- DGP: am 29.04.2025
- DGCH: am 15.05.2025
- DGN: am 17.04.2025
- DSH: am 11.04.2025
- DSG: am 11.04.2025
- DGAI: am 20.05.2025 (Empfehlungen 4.2 und 4.4 abgelehnt. Eine wissenschaftlich-fachliche Begründung befindet sich im Leitlinienreport: Kap. 5, Verabschiedung durch die Vorstände der herausgebenden Fachgesellschaften/Organisationen).
- DGIIN: am 22.04.2025
- DGHM: am 30.04.2025
- DGfN: am 19.05.2025
- DGMP: am 13.04.2025

## 16. Gültigkeitsdauer und Aktualisierungsverfahren

Die Leitlinie ist vom 30.04.2025 bis 30.04.2030 gültig. Die Gültigkeitsdauer beträgt maximal 5 Jahre und ist abhängig vom eingeschätzten Aktualisierungsbedarf. Kommentare und Hinweise für den Aktualisierungsprozess sind ausdrücklich erwünscht und können an das Leitliniensekretariat gesendet werden.

### Prof. Dr. Frank M. Brunkhorst:

E‑Mail: Frank.Brunkhorst@med.uni-jena.de; Deutsche Sepsis-Gesellschaft e. V., Waldenserstr. 2–4, 10551 Berlin.

## Abkürzungen

ABS: Antibiotic Stewardship
ADH: Antidiuretisches Hormon
ADM: Adrenomedullin
AKI: Acute Kidney Injury, akute Nierenschädigung
AMS: Antimicrobial Stewardship
AMSTAR: Assessing the Methodological Quality of Systematic Reviews
APACHE-II-SCORE: Acute Physiology And Chronic Health Evaluation
APRV: Airway Pressure Release Ventilation
aPTT: Aktivierte partielle Thromboplastinzeit
ARDS: Acute Respiratory Distress Syndrome, akutes Atemnotsyndrom
ART: Kommission Antiinfektiva, Resistenz und Therapie
ASPEN: American Society of Parenteral and Enteral Nutrition
AUROC: Area Under the Receiver Operating Characteristic
AWMF: Arbeitsgemeinschaft der Wissenschaftlichen Medizinischen Fachgesellschaften
BÄK: Bundesärztekammer
BfARM: Bundesamt für Arzneimittel und Medizinprodukte
CI: Confidence Interval, Konfidenzintervall
CIM: Critical Illness Myopathy
CIP: Critical Illness Polyneuropathy
CPAP: Continuous Positive Airway Pressure
CPFA: Coupled Plasma Filtration Adsorption
CRP: C‑Reactive Protein, C‑reaktives Protein
CRRT: Continuous Renal Replacement Therapy, kontinuierliche Nierenersatzverfahren
CRS: Compliance des respiratorischen Systems
CVP: Central Venous Pressure, zentraler Venendruck
CVVH: Kontinuierliche venovenöse Hämofiltration
CVVHD: Kontinuierliche venovenöse Hämodialyse
CVVHDF: Kontinuierliche venovenöse Hämodiafiltration
DGAI: Deutsche Gesellschaft für Anästhesiologie und Intensivmedizin
DGHO: Deutsche Gesellschaft für Hämatologie und Medizinische Onkologie
DIVI: Deutsche Interdisziplinäre Vereinigung für Intensiv und Notfallmedizin
DSG: Deutsche Sepsis-Gesellschaft
DSH: Deutsche Sepsis-Hilfe
DTP: Differential Time to Positivity
DVT: Deep Vein Thrombosis, tiefe Venenthrombose
EAA: Endotoxin Activity Assay, Endotoxinaktivität
ECMO: Extracorporeal Membrane Oxygenation, extrakorporale Membranoxygenierung
EGDT: Early Goal-Directed Therapy
EIT: Elektrische Impedanztomographie
ENG/EMG: Elektroneurographie/Elektromyographie
ESCMID: European Society of Clinical Microbiology and Infectious Diseases
ESICM: European Society of Intensive Care Medicine
GFP: Gefrorenes Frischplasma
GFR: Glomeruläre Filtrationsrate
GI: Gastrointestinal
GRADE: Grading of Recommendations Assessment, Development and Evaluation
HA: Humanalbumin
HAT: Hydrocortison, Ascorbinsäure und Thiamin
HES: Hydroxyethylstärke
HFNO: High-Flow Nasal Oxygen
HIT: Heparin-induzierte Thrombozytopenie
HZV: Herzminutenvolumen
IBW: Ideal Body Weight, ideales Körpergewicht
ICD: International Statistical Classification of Diseases and Related Health Problems
ICU-AW: ICU-Acquired Weakness
IHD: Intermittierende Hämodialyse
IgM: Immunglobulin M
IPC: Intermittent Pneumatic Compression, intermittierende pneumatische Kompression
ITS: Intensivstation
IVIG: Intravenöse Immunglobuline
KDIGO: Kidney Disease: Improving Global Outcomes
KI: Künstliche Intelligenz
KRINKO: Kommission für Krankenhaushygiene und Infektionsprävention am Robert Koch-Institut
LAE: Lungenarterienembolie
MAP: Mean Arterial Pressure, mittlerer arterieller Blutdruck
MEWS: Modified Early Warning Score
MHK: Minimale Hemmkonzentration
MIQ: Mikrobiologisch-infektiologische Qualitätsstandards
MLP: Methylenblau-Licht-behandeltes Plasma
MODS: Multiorganversagen
MRE: Multiresistente Erreger
MRGN: Multiresistente gramnegative Bakterien
MRSA: Methicillin-resistenter Staphylococcus aureus
NaCl: Natriumchlorid
NET: Nierenersatztherapie
NEWS: National Early Warning Score
NIV: Nichtinvasive Beatmung
NMH: Niedermolekulares Heparin
OPSI: Overwhelming Post-Splenectomy Infection
OR: Odds Ratio
PCR: Polymerase Chain Reaction
PCT: Procalcitonin
PEEP: Positiver endexspiratorischer Druck
PEG: Paul-Ehrlich-Gesellschaft für Infektionstherapie
PICS: Post-Intensive-Care-Syndrom
PIRRT: Prolonged Intermittent Renal Replacement Therapy
PPV: Pulsdruckkurvenvariation
PTBS: Posttraumatische Belastungsstörung
PTPe: Endexspiratorischer transpulmonaler Druck
RAAS: Renin-Angiotensin-Aldosteron-System
RASS: Richmond Agitation-Sedation Scale
RCT: Randomized Controlled Trial
RR: Relatives Risiko
RRT: Renal Replacement Therapy
RVS: Humanes respiratorisches Synzytial-Virus
SAE: Serious Adverse Event
SCCM: Society of Critical Care Medicine
SCD: Sequential Compression Devices, sequenzielle Kompressionsgeräte
ScvO_2_: Central Venous Oxygen Saturation
SDP: Solvent-Detergent-behandeltes Plasma
sGC: Guanylatzyklase
SIRS: Systemic Inflammatory Response Syndrome
SLEDD: Sustained Low Efficient Daily Dialysis
SOFA: Sequential Organ Failure Assessment Score
SOP: Standard Operating Procedure, Standardarbeitsanweisungen
SSC: Surviving Sepsis Campaign
STIKO: Ständige Impfkommission
SVV: Schlagvolumenvariation
TDM: Therapeutisches Drugmonitoring
TEE: Transösophageale Echokardiographie
TTE: Transthorakale Echokardiographie
TVT: Tiefe Venenthrombose
UFH: Unfraktioniertes Heparin
URR: Urea Reduction Rate
VRE: Vancomycin-resistente Enterokokken
VV-ECMO: Venovenöse extrakorporale Membranoxygenierung
WHO: World Health Organisation
ZVK: Zentraler Venenkatheter

## Footnotes

1 Die Empfehlung wurde bei Vorlage der Konsultationsfassung durch die Deutsche Gesellschaft für Anästhesiologie und Intensivmedizin e.V. (DGAI) abgelehnt. Eine wissenschaftlich-fachliche Begründung befindet sich im Leitlinienreport (Kap. 5, Verabschiedung durch die Vorstände der herausgebenden Fachgesellschaften/Organisationen).

2 Die Leitlinie war zum Zeitpunkt der Veröffentlichung noch nicht publiziert.

## Notes

The supplement containing this article is not sponsored by industry.
